# Profiling the heterogeneity of colorectal cancer consensus molecular subtypes using spatial transcriptomics

**DOI:** 10.1038/s41698-023-00488-4

**Published:** 2024-01-10

**Authors:** Alberto Valdeolivas, Bettina Amberg, Nicolas Giroud, Marion Richardson, Eric J. C. Gálvez, Solveig Badillo, Alice Julien-Laferrière, Demeter Túrós, Lena Voith von Voithenberg, Isabelle Wells, Benedek Pesti, Amy A. Lo, Emilio Yángüez, Meghna Das Thakur, Michael Bscheider, Marc Sultan, Nadine Kumpesa, Björn Jacobsen, Tobias Bergauer, Julio Saez-Rodriguez, Sven Rottenberg, Petra C. Schwalie, Kerstin Hahn

**Affiliations:** 1 Roche Pharma Research and Early Development, Roche Innovation Center Basel, Basel, Switzerland; 2 Institute of Animal Pathology, Vetsuisse Faculty, University of Bern, Bern, Switzerland; 3 Roche Pharma Research and Early Development, Roche Innovation Center Zurich, Schlieren, Switzerland; 4 Genentech, Inc, San Francisco, CA USA; 5 Faculty of Medicine and Heidelberg University Hospital, Institute of Computational Biomedicine, Heidelberg University, Heidelberg, Germany; 6 Bern Center for Precision Medicine (BCPM), University of Bern, Bern, Switzerland

**Keywords:** Tumour heterogeneity, Cancer microenvironment, Colon cancer, Cancer imaging, Computational biology and bioinformatics

## Abstract

The consensus molecular subtypes (CMS) of colorectal cancer (CRC) is the most widely-used gene expression-based classification and has contributed to a better understanding of disease heterogeneity and prognosis. Nevertheless, CMS intratumoral heterogeneity restricts its clinical application, stressing the necessity of further characterizing the composition and architecture of CRC. Here, we used Spatial Transcriptomics (ST) in combination with single-cell RNA sequencing (scRNA-seq) to decipher the spatially resolved cellular and molecular composition of CRC. In addition to mapping the intratumoral heterogeneity of CMS and their microenvironment, we identified cell communication events in the tumor-stroma interface of CMS2 carcinomas. This includes tumor growth-inhibiting as well as -activating signals, such as the potential regulation of the ETV4 transcriptional activity by DCN or the PLAU-PLAUR ligand-receptor interaction. Our study illustrates the potential of ST to resolve CRC molecular heterogeneity and thereby help advance personalized therapy.

## Introduction

CRC is a leading cause of cancer-related death worldwide with over 1.85 million diagnosed cases and 850000 deaths annually^1^. Despite a decline in mortality rates due to personalized treatments in recent years^2^, the extensive inter-patient and intra-tumor heterogeneity of CRC still pose substantial treatment challenges^3^. This heterogeneity manifests at genomic, epigenomic and transcriptomic levels, and in the composition of the tumor microenvironment (TME)^4^.

In 2015, the CRC subtyping consortium proposed a classification of CRC into four CMS, derived from large-scale gene expression datasets^5^. Despite its widespread use, its clinical impact is still limited due to its reliance on bulk-sequencing, which cannot accurately categorize mixed or transitional CMS phenotypes, nor precisely define the cellular composition and microenvironment of tumors. Recently, scRNA-seq was applied to CRC samples, revealing CMS features at the cellular level and the coexistence of multiple CMS in individual patients^6–10^. However, the spatial distribution of the different CMS and their interactions with their respective TMEs remain poorly understood.

ST technologies can address these limitations by measuring gene expression levels throughout tissue space, integrating morphology, spatial localization and transcriptomic profile. In oncology, ST has been employed to study breast cancer^11^, prostate cancer^12^ and melanoma^13^, among others. To date, its application to CRC has been mostly to support results obtained from other technologies, without specifically addressing the CMS of CRC^14–17^.

Here, we applied ST to analyze 14 samples from seven CRC patients, aiming to deepen our understanding of the spatial properties and heterogeneity of CMS. By mapping cell type composition spatially, linking distinct molecular and morphological features to different CMS, and investigating predicted intercellular interactions in CMS2 carcinomas, we highlighted the capacity of ST to support the future development of personalized treatment strategies for CRC.

## Results

### ST and deconvolution reliably reveal the spatial cell type distribution in CRC

**Fig. 1:**
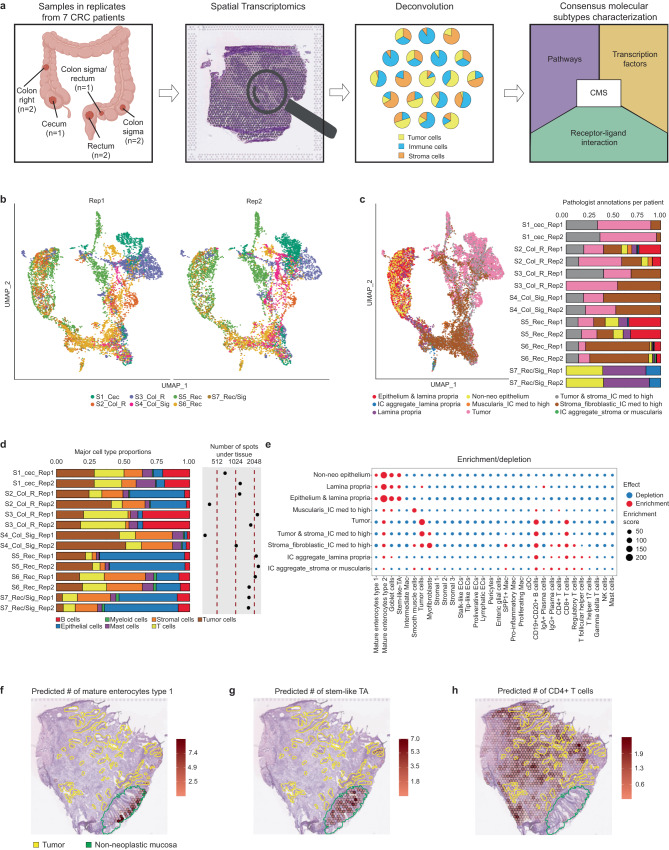
Study outline and deconvolution results matching histopathological annotations with high correlation between replicates. **a** Study outline displaying the anatomical localization of our set of CRC samples, their spatial transcriptomics processing and the deconvolution-based approach to characterize spatial features of CMS. Figures created with BioRender.com. **b** UMAP embedding of the gene expression measurements per spot split by technical replicates. Colors represent the different patients. **c** UMAP embedding of the gene expression measurements per spot colored by pathologist’s annotations. In addition, a bar plot displays the proportions of these annotations per sample. IC: immune cells. **d** Proportions of major cell classes per sample as estimated by the results of the deconvolution approach. The right hand side of the plot displays the number of analyzed spots per sample. **e** Enrichment/depletion plot describing the association between cell type abundance as predicted by the deconvolution (x-axis) and the different anatomical regions as annotated by the pathologist (y-axis). The dot size represents the enrichment score (Methods), while the color represents enrichment (red) or depletion (blue). IC immune cells. **f**–**h** Spatial mapping of the predicted number of mature enterocytes type I, stem-like transient amplifiers (TA) and CD4+ T cells per spot matching the pathologists’ tissue annotation and expected cell type localization as illustrated for sample S6_Rec_Rep2.

We used 10x Genomics VISIUM to process fresh-frozen resection samples from CRC tumors of seven individuals, obtained from different anatomical locations, and exhibiting varying metastatic status, growth patterns, and immune cell (IC) infiltration levels (Fig. 1a, Table 1). We considered two serial sections per patient to generate technical replicates, resulting in a total of 20,733 Visium spots, each of which contained an average of 3,738 unique genes (Supplementary Fig. S1a). Technical reproducibility among the replicates, along with inter-patient heterogeneity, were revealed via the UMAP projection of the transcriptomic profiles of the aforementioned spots (Fig. 1b). A pathologist independently examined the samples and assigned each spot to its corresponding anatomical compartment based on tissue type and cellular morphology (Fig. 1c, Methods).

**Table 1:** Selected clinical information for the samples included in this study.

Sample number	Localization	Diagnosis	Pre- treatment	Lymph node/liver metastasis	Mutation	Growth pattern and Immune cells
S1_Cec A551763	Cecum	Adenocarcinoma, mucinous, moderately differentiated	No	Yes/yes	BRAF V600E	Mucinous, IC low
S2_Col_R A595688	Colon (right)	Adenocarcinoma, moderately differentiated	No	Yes/yes	No KRAS mutations	Tubular to cribriform, IC low
S3_Col_R A416371	Colon (right)	Adenocarcinoma, areas with moderate and poor differentiation	No	Yes/no	–	2 tumor types: I) tubular, IC low; II) extended solid, IC high
S4_Col_Sig A120838	Colon (Sigma)	Adenocarcinoma, moderately differentiated	No	Yes/no	No KRAS mutations	Tubular to cribriform, IC low
S5_Rec A121573	Rectum	Adenocarcinoma, moderately differentiated^a^	No	Yes/yes	–	Tubular to cribriform, IC low
S6_Rec A938797	Rectum	Adenocarcinoma, moderately differentiated^a^	No	No/no	–	Tubular, IC medium
S7_Rec/Sig A798015^a^	Sigma/Rectum	non-neoplastic tissue	na	na	–	na

*na* not applicable, – mutation profile was not assessed, *IC* Immune cell content.

^a^Sample contains non neoplastic tissue.

To determine the cellular composition per spot, we used Cell2Location^18^ and a recently published CRC scRNA-seq dataset^6^ as reference (Supplementary Table S1, Methods). We found highly comparable proportions between replicates when considering major cell types (Fig. 1d). In contrast, proportions varied greatly across individuals: for instance, unlike all other patients, S7_Rec/Sig samples mainly contained non-neoplastic tissue (Table 1), and tumor cells only comprised around 5%. Upon assessing the deconvolution results by computing the spatial correlation of cell subtype abundance among technical replicates, we found high stability with Pearson’s correlation coefficients over 0.9, except for a low-quality sample (Supplementary Fig. S1b, Methods).

We next evaluated whether the deconvolution-predicted cell type abundances were located in their respective anatomical compartments using the pathologist’s annotations as reference (Supplementary Fig. S1c). As expected, non-neoplastic intestinal cells were the most abundant in non-neoplastic epithelium (89%), while T and B cells were the prevalent types in the immune cell aggregates (83% and 68%). In tumor-annotated spots, tumor cells (36%), T cells (26%), and B cells (25%) were the predominant types. At the cell subtype level, we observed significant enrichment of non-neoplastic mucosal cells, such as mature enterocytes type 1 and 2, goblet cells and stem-like transiently amplifying cells in spots labeled as non-neoplastic epithelium, lamina propria or mixed (Fig. 1e, Methods). Tumor cells, CD19^+^CD20^+^ B cells and CD8^+^ T cells were mainly enriched in spots classified as tumor or tumor-stroma mixed. CD4^+^ T-cells and other immune cells were mostly found in IC aggregates and stromal regions with high IC content. The agreement between the pathologist’s annotations and deconvolution results was also evident when visualizing the individual samples in more detail (Fig. 1f–h, Supplementary Figs. S2 and S3).

In summary, the estimated cell type abundances were consistent across technical replicates, and their spatial distribution aligned with the pathologist’s assessment for all samples.

### Spatially resolved consensus molecular subtyping of CRC

**Fig. 2:**
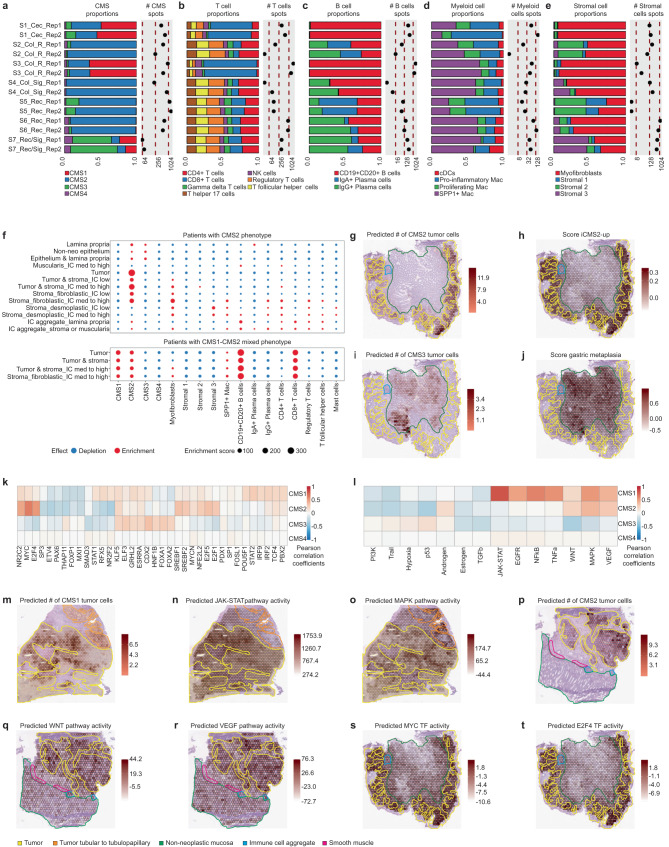
Consensus molecular subtyping of our set of CRC samples, characterization of their TME and spatially resolved mapping of their histological and molecular features. **a**–**e** Cell type proportions per sample as estimated by the results of the deconvolution. The number of spots containing an abundance of at least 20% of the specified cell types is also displayed. NK natural killers, Mac Macrophages, cDCs conventional dendritic cells. **f** Enrichment/depletion assessment of selected cell types (x-axis) in CMS2 and mixed CMS1-CMS2 tumors in the different tissue compartments defined by the pathologist’s spot classification (y-axis). IC immune cells. **g**–**j** Spatial mapping of the predicted abundance of CMS2 and CMS3 tumor cells and the module scores of the iCMS2-upregulated and the gastric metaplasia signatures overlaid with the pathologist’s tissue annotation in the S5_Rec_Rep1 sample. **k** Per spot Pearson’s cross-correlation across all the samples between TF activities and CMS cell abundances. For visualization purposes, the 10 most highly correlated TFs in absolute value per CMS are shown. **l** Per spot Pearson’s cross-correlation across all the samples between pathway activities and CMS cell abundances. **m**–**o** Spatial mapping of the predicted abundance of the CMS1 cells, the JAK-STAT pathway activity and the MAPK pathway activity overlaid with the pathologist’s tissue annotation in the S3_Col_R sample. **p**–**r** Spatial mapping of the predicted abundance of the CMS2 cells, the WNT pathway activity and the VEGF pathway activity overlaid with the pathologist’s tissue annotation in the S2_Col_R_Rep1 sample. **s**, **t** Spatial mapping of the predicted transcriptional activity of the MYC and E2F4 TFs overlaid with the pathologist’s tissue annotation in the S5_Rec_Rep1 sample. Note the colocalization with CMS2 tumor cell abundance (Fig. 2g).

We further utilized the deconvolution results and pathologist’s annotations to spatially characterize the TME and CMS (Supplementary Figs. S4 and S5). CMS2 tumor cell proportions were predominant in patient samples S2_Col_R (94%), S4_Col_Sig (98%), S5_Rec (81%), and S6_Rec (90%) (Fig. 2a). A mixed abundance of CMS1 and CMS2 tumor cells was identified in patients S1_Cec (49% and 41%) and S3_Col_R (65% and 29%). Additionally, CMS3 tumor cells were detected in the S1_Cec (10%) and S5_Rec (16%) patients. In the non-neoplastic S7_Rec/Sig sample, the few spots exhibiting a tumor cell signature were mainly classified as CMS3 (60%). The prevalence of CMS4 was low and showed a multifocal distribution that overlapped with anatomical regions presenting an invasive phenotype. To characterize the TME composition, we next computed immune and stromal cell proportions (Fig. 2b–e). Mixed CMS1-CMS2 tumors exhibited higher T and B cell proportions, particularly CD8^+^ T and CD19^+^CD20^+^ B cells, consistent with the immune-rich phenotype associated with CMS1^5^. Myofibroblasts were the dominant stromal cell type in mixed CMS1-CMS2 tumors, while the stromal cell types in CMS2 neoplasms were more heterogeneous. This is consistent with previous scRNA-seq studies reporting myofibroblast prevalence in CMS1 tumors^6,7^.

We next associated these results with histological and morphological features by computing cell subtype enrichment in the pathologist-defined tissue compartments (Fig. 2f, Methods). CMS1 and CMS2 signatures were associated with tumor-annotated spots, while CMS3 signatures were confined to non-neoplastic mucosa. In CMS2-dominant tumors, immune cells were mostly found in the stroma, whereas in mixed CMS1-CMS2 tumors, CD19^+^CD20^+^ B and CD8^+^ T cells were also present in the neoplastic tissue. Irrespective of the CMS phenotype, SPP1+ macrophages and myofibroblasts were enriched in stromal fibrotic regions, echoing recent findings showing that proportions of these populations influence prognosis beyond CMS classification^7^.

We also connected our deconvolution-based CMS classification with the recently introduced IMF classification, which integrates intrinsic epithelial subtypes, microsatellite instability status, and fibrosis^8^. The predicted CMS2 abundance correlated significantly with the intrinsic epithelial subtype CMS2 (iCMS2) signature score (Fig. 2g, h, Supplementary Fig. S6). CMS2 - iCMS2 correspondence was additionally supported by mutational profiles (Table 1), anatomical location (Table 1), microsatellite instability status (Supplementary Fig. S7), and tubular adenoma and crypt bottom marker associations (Supplementary Fig. S8). Further, CMS3 signals were associated with key molecular features of iCMS3, including gastric metaplasia (Fig. 2i, j, Supplementary Fig. S9), upper crypt signals, and sessile serrated lesion markers (Supplementary Fig. S10).

Interestingly, we demonstrated that ST can spatially resolve known CMS-associated molecular features (Fig. 2k, l, Methods), such as the correlation between CMS1 tumor cell abundance and activity of the immune-related pathways JAK-STAT^19^ (Fig. 2m, n), TNFα^20^ and NFkB. Additionally, activation of the MAPK pathway (Fig. 2o), which is characteristic of the hypermutated CMS1^21^, was observed. For CMS2 tumor cells, we identified their known association with the activation of the WNT and VEGF pathways^22^ (Fig. 2p–r) and higher expression of MYC- and E2F4-regulated genes^5^ (Fig. 2s, t).

Hence, our deconvolution-based approach spatially mapped the different CMS and TME cell types to their expected tissue compartments and associated them with key molecular and histological features.

### ST reveals inter-patient and intra-patient heterogeneity of CRC tumors

**Fig. 3:**
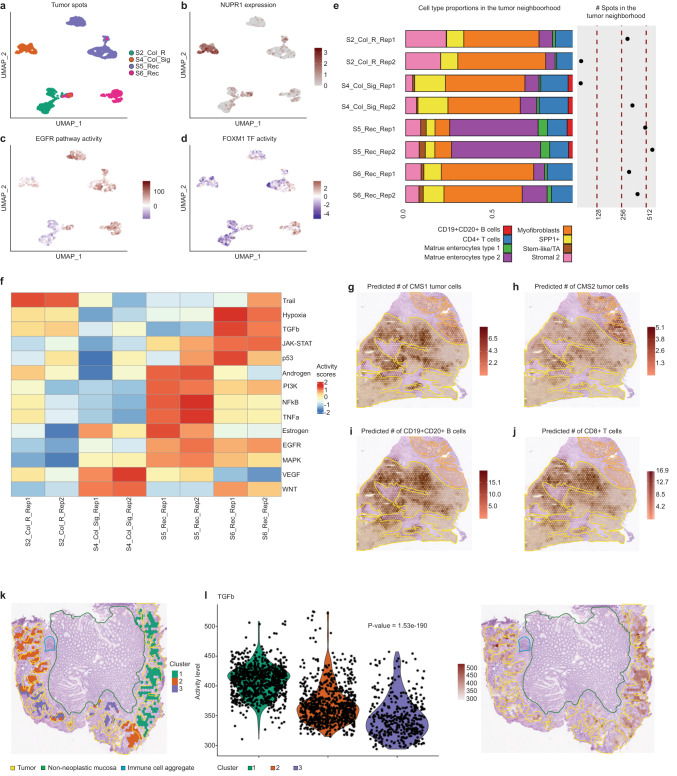
Inter- and intra-patient heterogeneity in CRC tumors and their TME in terms of cell composition and different molecular features. **a**–**d** UMAP embeddings of the gene expression measurements in tumor annotated spots which were colored by different criteria: **a** per patient, **b** per the expression of the NUPR1 gene, **c** per activity of the EGFR pathway and **d** per activity of the FOXM1 TF. **e** Cell type proportions in the tumor-surrounding spots per sample as estimated by the results of the deconvolution approach. The number of tumor-surrounding spots for the different samples is also displayed. TA transient amplifiers. **f** Differential pathway activity computed on pseudo-bulk RNA-seq generated from the tumor-surrounding spots for the different samples. **g**–**j** Spatial mapping of the predicted abundance of CMS1, CMS2, CD19^+^CD20^+^ B cells and CD8^+^ T cells overlaid with the pathologist’s tissue annotation in the S3_Col_R sample. **k** Overlay of the spatial mapping of the clustering at subspot enhanced resolution of the tumor-annotated spots with the pathologist’s tissue annotations in the S5_Rec_Rep1 sample. **l** Spatial mapping and violin plots per group of the TGFb pathway activity at the enhanced subspot resolution in the S5_Rec_Rep1 sample. A Kruskal–Wallis statistical test was performed to assess whether the pathway activities in the different subclusters originated from the same distribution (*p*-value).

To assess and further characterize the inter-patient heterogeneity among CMS2 tumors^7,23^, we extracted tumor-annotated spots from the CMS2-dominant carcinomas: S2_Col_R, S4_Col_Sig, S5_Rec, and S6_Rec (Supplementary Fig. S11). Although CMS2 cells dominated these spots with abundances ranging from 65% to 84% (Supplementary Fig. S12a, b), differential gene expression, pathway, and TF activity analyses (Fig. 3a–d, Supplementary Table S2) unveiled significant inter-patient differences. For instance, we found overrepresented mTORC1 signaling genes in tumors from the S4_Col_Sig and S5_Rec patients, but differentially expressed genes within this pathway suggested alternative signaling cascades (Supplementary Table S3). Notably, NUPR1, a promoter of metastasis through activation of the PTEN/AKT/mTOR pathway^24^, was highly expressed only in CMS2 tumor cells from the S4_Col_Sig patient (Fig. 3b). Tumor spots from the S2_Col_R and S4_Col_Sig patients showed lower EGFR signaling (Fig. 3c, Supplementary Fig. S12c), while FOXM1 displayed higher transcriptional activity in patient S6_Rec (Fig. 3d, Supplementary Fig. S12d).

Inter-patient transcriptomic differences in CMS2 tumors can arise from inherent heterogeneity, anatomical origin and the composition and architecture of the TME. The latter can be uniquely assessed using ST. By selecting the spots surrounding CMS2 tumors, we assessed differential pathway activity among patients (Fig. 3e, f, Methods). The S5_REC patient exhibited a depletion of myofibroblasts (Supplementary Fig. S12e), potentially explaining its lower TGFβ pathway activity^25^. In S4_Col_Sig, the higher proportion of SPP1^+^ macrophages (Supplementary Fig. S12g), may contribute to an immunosuppressive TME^26^, in line with its lower activities in immune response-associated pathways such as NFκB and TNFα. The proportions and spatial distributions of these specific cell types are crucial as they drive clinical outcomes, with higher proportions linked to poorer prognosis^7^.

The assessment of the CMS1/CMS2 mixed sample S3_Col_R highlights the power of ST to characterize the CMS heterogeneity within a patient’s tumor and its associated morphologic features. CMS1-dominated regions displayed a solid growth pattern and immune-rich profile, whereas CMS2-dominated regions were associated with a tubular growth pattern and were immune-deprived (Fig. 3g–j, Supplementary Fig. S4d), in accordance with previous studies on these molecular subtypes^27^.

We subsequently addressed the intra-tumor heterogeneity in tumors displaying a pronounced CMS2 phenotype. To illustrate this, we categorized tumor-annotated spots from the S2_Col_R_Rep1 sample into peripheral, intermediate, and central tumor areas (Methods). As expected, genes involved in epithelial-mesenchymal transition (EMT) and angiogenesis, such as *SPARC*^28^, were significantly upregulated in the tumor boundary (Supplementary Fig. S13a, c, Supplementary Table S3, Methods). In contrast, the central tumor area showed an increased activity in hypoxic response and cholesterol homeostasis pathways, putatively driven by the upregulation of genes like *SCD* (Supplementary Fig. S13b, d, Supplementary Table S3). *SCD* upregulation was previously associated with the metabolic reprogramming necessary to promote metastasis of CRC cancer cells^29^. We finally sub-clustered the tumor-annotated spots extracted from S5_Rec_Rep1 (Fig. 3k, Methods) and identified regions with differentially expressed genes, biological processes, and pathway activities (Supplementary Figs. S14 and S15, Supplementary Table S4). Notably, CMS2-associated WNT and VEGF pathways displayed a more consistent distribution of their activities across tumor regions as compared to the activity of EGFR and MAPK pathways. Similarly, subcluster 1 demonstrated increased TGFβ pathway activity, suggesting tumor regions with higher proliferation and metastatic potential^30^ (Fig. 3l).

Together, our results demonstrate how ST unveils inter- and intra-tumor heterogeneity, TME architecture and spatial patterns of key molecular processes in CRC.

### ST charts cell-to-cell communication processes involved in CMS2 tumor progression

The power of ST is that it reveals the cellular organization of the tissue at the molecular level, and thereby allows the study of cell communication events. We therefore explored these processes at the tumor-stroma interface and investigated their potential involvement in the tumor progression of the CMS2 subtype.

**Fig. 4:**
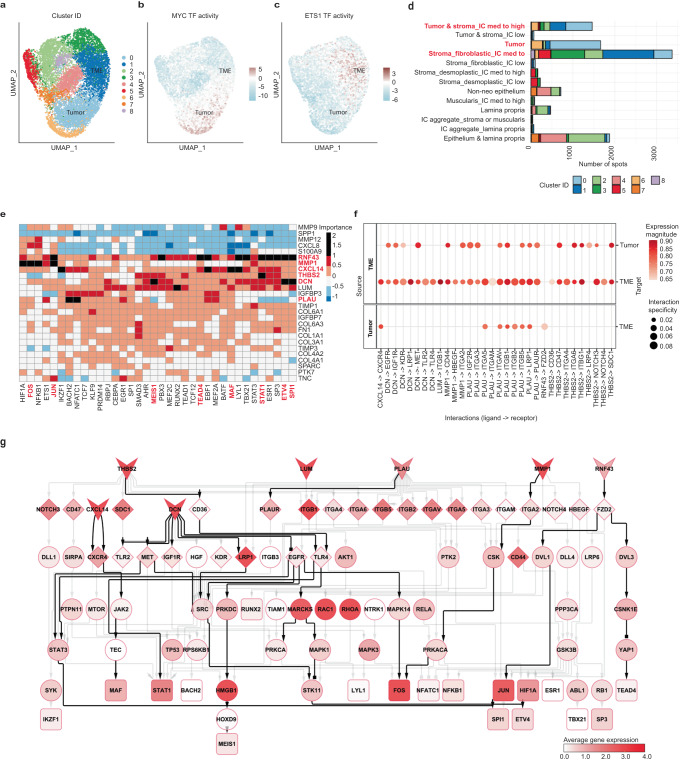
Clustering based on TF activities to study cell communication events at the tumor-stroma interface of CMS2 tumors. The signaling cascades triggered by those events and leading to transcriptional activities related to tumor progression were also investigated. **a**–**c** UMAP embedding of the TF activity profiles for our set of CMS2 samples. The spots were colored following different criteria: **a** per cluster group, **b** per activity of the MYC TF, and **c** per activity of the ETS1 TF. **d** Number of spots belonging to the different categories of pathologist’s annotations and clusters as inferred from the TF activity profiles. IC immune cells. **e** Misty results showing the potential importance of ligands (rows) expression on TF (columns) activity. The ligand-TFs relationships with an importance score over 1 are represented as black slots and were further investigated. Bold red characters were used to highlight the name of the ligands and TFs referred to in the main text. **f** Top predicted ligand-receptor interactions at the tumor stroma interface. The left panel shows the source of the interaction (ligands) and the right the target (receptors). **g** Signaling cascades potentially linking ligands (V shape) to their downstream TF targets (triangles) according to Misty predictions. The downstream signaling cascades go first through the top predicted receptors (diamonds) and then to intermediary signaling proteins (ellipses). The color of the nodes indicates the average expression of these genes in the TME cluster. Network edges can represent stimulatory (arrows) or inhibitory (squares) interactions. The edges representing interactions referred to in the main text are highlighted in black.

To study conserved biological processes across our CMS2 tumor samples (S2_Col_R; S4_Col_Sig; S5_Rec; S6_Rec), we merged and clustered their spots based on TF activity profiles (Fig. 4a–c, Supplementary Fig. S16, Methods). This approach revealed higher similarity as compared to gene expression-based clustering, and was hence used for our downstream analysis. Cluster 0, hereafter referred to as the tumor cluster, contained mainly spots annotated as tumor (49%) and tumor&stroma_IC med to high (26%) across replicates and patients (Fig. 4d, Supplementary FIg. S17a). Cluster 1, hereafter referred to as the TME cluster, predominantly included stromal annotated spots (63% as stroma_fibroblastic_IC med to high and 20% as tumor&stroma_IC med to high), neighboring the tumor in every sample (Fig. 4d, Supplementary Fig. S17a). As expected, MYC and E2F4 were highly activated TFs in the tumor cluster, while TFs such as JUN and ETS1, were identified in the TME cluster (Fig. 4b, c, Supplementary Fig. S17b).

**Fig. 5:**
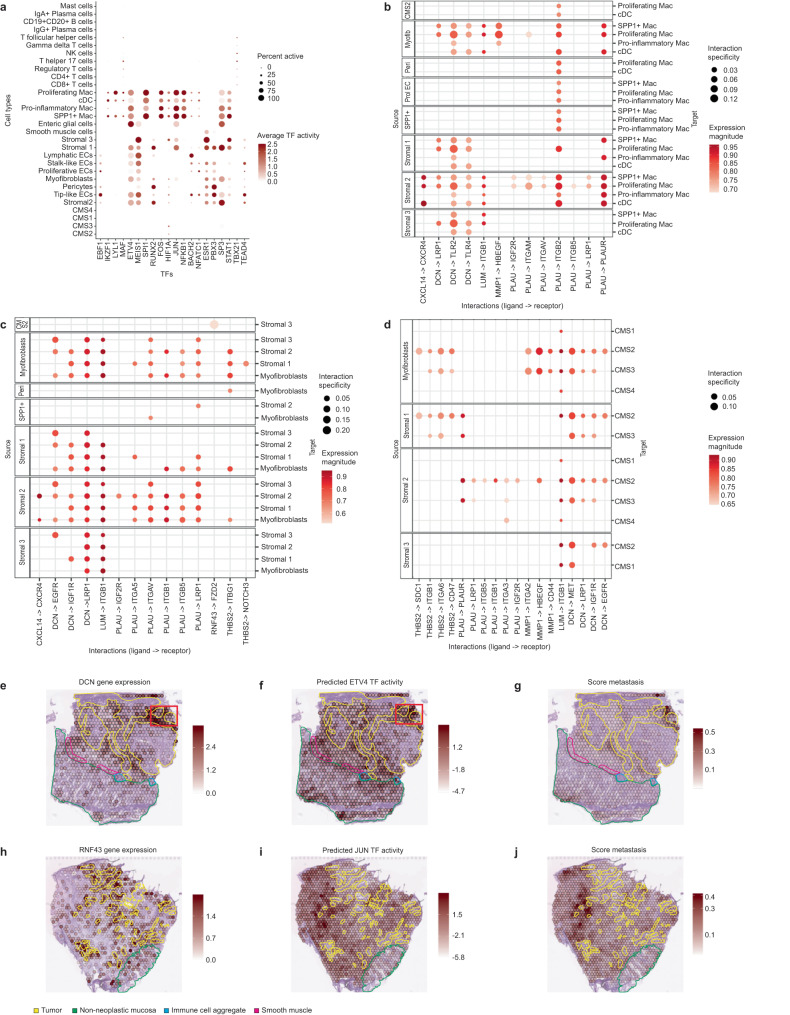
Transcription factor activity and ligand-receptor interactions in the scRNA-seq from Lee et al. Spatial maps showing gene expression, TF activity and a score for selected tumor-associated processes. **a** Average TF activity per cell type. The percentage of cells of a given type where the TF is active is represented by the size of the circle. NK: natural killers, Mac: macrophages, cDCs: conventional dendritic cells, ECs: endothelial cells. **b**–**d** Ligand-receptor interactions between the different cell types overlapping with the interactions predicted in our ST data. The left panel shows the source of the interaction (ligands) and the right the target (receptors): **b** target cell types are myeloid cells, **c** target cell types are the major stromal cell populations, and **d** target cell types are the different CMS tumor cell types. Mac macrophages, cDCs conventional dendritic cells. **e**–**g** Overlay of the DCN gene expression, the predicted ETV4 TF activity and the metastasis score with the pathologist’s tissue annotations in the S2_Col_R_Rep1 sample. A red square highlights the tumor region exhibiting invasive morphological traits. **h**–**j** Overlay of the RNF43 gene expression, the predicted JUN TF activity and the metastasis score with the pathologist’s tissue annotations in the S6_Rec_Rep2 sample.

We then estimated the potential influence of ligands highly expressed in the tumor and TME compartments on the transcriptional activity of stroma-enriched TFs using Misty^31^ (Fig. 4e, Methods). We connected the most consistent ligand-TF associations to putative upstream signaling by predicting inter-cellular ligand-receptor interactions at the tumor-stroma interface and their known signaling pathways (Fig. 4f, g). To validate the ST-derived signaling events and to identify the involved cell types, we additionally estimated TF activity and ligand-receptor interactions in CMS2 patients from the Lee et al.’s scRNA-seq dataset^6^ (Fig. 5a–d, Supplementary Fig. S18a, Methods).

Our results suggested that decorin (DCN), a proteoglycan secreted by stromal cells, triggers a protective pathway inhibiting tumor progression in the CMS2 subtype. DCN interacts with receptors like EGFR, IGF1R and MET, promoting their degradation and impairing downstream signaling, as described in previous studies^32^. The DCN-EGFR-SRC-STK11, DCN-EGFR-PRKDC-HMGB1-HOXD9 and DCN-MET-STAT3 signaling axis may modulate the transcriptional activity of ETV4, MEIS1 and SPI1 respectively, as supported by our findings in the ST and scRNA-seq data (Figs. 4e–g, 5a–f, Supplementary Fig. S18b–d). Increased activity of these TFs is associated with greater tumor invasiveness^33–35^. The spatial mapping of ETV4 transcriptional activity revealed overall low levels within the tumor, excepting for a region exhibiting invasive morphological traits and higher macrophage infiltration (Fig. 5e, f, Supplementary Figs. S2b and S18f). Our findings capture DCN’s effects on these macrophages through its interaction with the TLR2 and TLR4 receptors (Figs. 4f, 5b, Supplementary Fig. S18e). In summary, our results highlight DCN’s pivotal role in tumor suppression, particularly in CMS2 regions with elevated invasiveness potential (Fig. 5g).

Moreover, our data indicated that the CMS2-associated RNF43, a transmembrane protein, might influence several TFs within the TME, including JUN and TEAD4 (Figs. 4e, 5h, i, Supplementary Fig. S19a, b). Notably, these TFs are involved in tumor progression and associated with WNT signaling^36,37^. We predicted an RNF43-FZD2 interaction targeting stromal cell populations (Figs. 4f, 5c, Supplementary S19c, d), and signaling cascades connecting these elements, such as the FZD2-DVL3 and the YAP-TEAD4 interactions (Fig. 4g). In summary, elevated RNF43 expression increases WNT receptor degradation, affecting downstream transcriptional activity, and potentially indicating anatomical regions with lower metastatic activity (Fig. 5j).

In addition, we identified other ligand-TF pairs potentially modulating CMS2 tumor progression. For instance, the THBS2-CD36 interaction, known to inhibit angiogenic processes^38^, may modulate STAT1 activity (Fig. 4e, f, Supplementary Fig. S19e–h). The expression of *MMP1*, a matrix metalloproteinase involved in cancer progression through degradation of the extracellular matrix^39^, was predicted to have an effect on the activity of the FOS TF (Fig. 4e, Supplementary Fig. S18g, h). The PLAU-PLAUR interaction was identified between myofibroblasts and macrophages or conventional dendritic cells (Figs. 4f, 5b, Supplementary Fig. S18i–k), consistent with prior studies in prostate cancer, associating this interaction with macrophage infiltration and tumor progression^40^. Moreover, we found that chemokine CXCL14 could influence MAF transcriptional activity (Fig. 4e), which was shown to regulate the immunosuppressive function of tumor-associated macrophages^41^. Interestingly, a CXCL14-based peptide has previously been suggested as a potential cancer treatment^42^.

In conclusion, our results generate mechanistic hypotheses on how highly expressed ligands in CMS2 tumors and their TME may trigger signaling cascades modulating TFs involved in cancer progression.

### Deconvolution-based subtyping, heterogeneity and cell communication events confirmed in independent CRC cohort

To corroborate our findings, we analyzed an independent ST dataset^14^, comprising four primary CRC tumors exhibiting morphological features indicative of CMS2, along with their corresponding liver metastases. The samples were obtained from two untreated (*Unt*) and two neoadjuvant chemotherapy-treated patients (*Tre*).

**Fig. 6:**
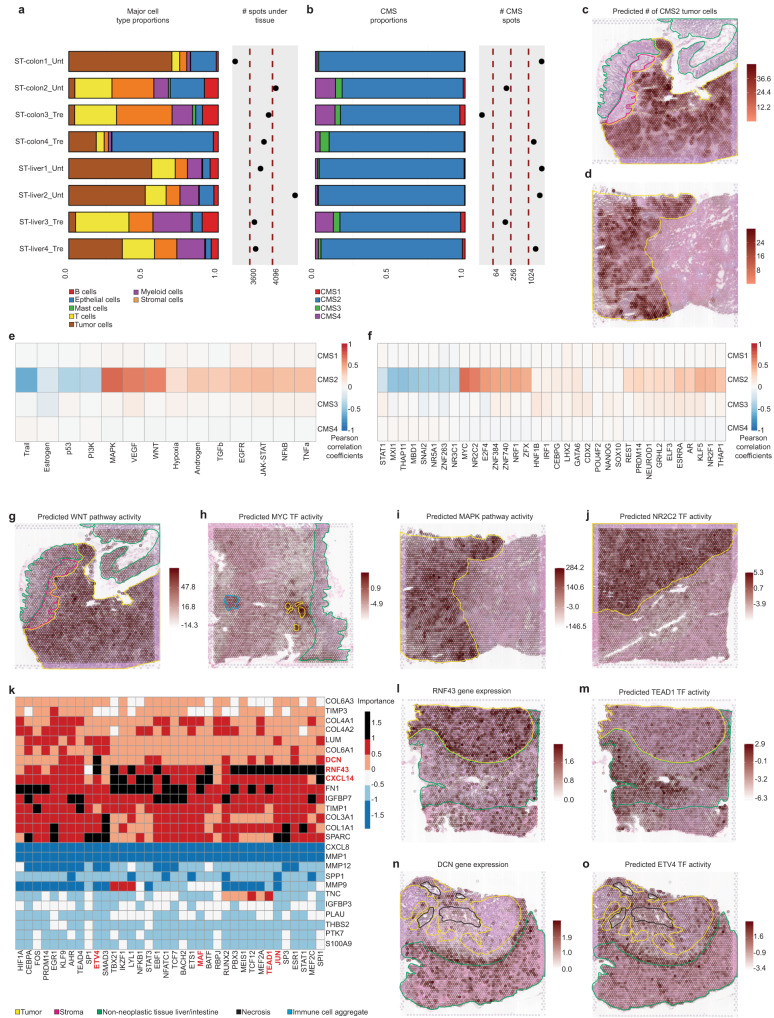
Characterization and analysis of an external ST CRC dataset to support the results in our internal set of samples. **a** Proportions of major cell classes per sample as estimated by the results of the deconvolution. The right hand side of the plot displays the number of analyzed spots per sample. **b** CMS tumor cell type proportions per sample as estimated by the results of the deconvolution approach. The number of spots containing an abundance of at least 20% of tumor cells subtypes is also displayed. **c**, **d** Overlay of the spatial mapping of the predicted CMS2 tumor cell abundance with the pathologist’s tissue annotations in the ST-colon1_Unt and ST-liver1_Unt samples. **e** Per spot Pearson’s cross-correlation across all the samples between pathway activities and CMS cell abundances. **f** Per spot Pearson’s cross-correlation across all the samples between TF activities and CMS cell abundances. For visualization purposes, the 10 most highly correlated TFs in absolute value per CMS are shown. **g** Overlay of the spatial mapping of the predicted WNT pathway activity with the pathologist’s tissue annotations in the ST-colon1_Unt sample. **h** Overlay of the spatial mapping of the predicted MYC TF activity with the pathologist’s tissue annotations in the ST-colon2_Unt sample. **i** Overlay of the spatial mapping of the predicted MAPK pathway activity with the pathologist’s tissue annotations in the ST-liver1_Unt sample. **j** Overlay of the spatial mapping of the predicted NR2C2 TF activity with the pathologist’s tissue annotations in the ST-liver2_Unt sample. **k** Misty results showing the potential importance of ligands (rows) expression on TF (columns) activity when considering the samples from primary CRC tumors. The ligand-TFs relationships with an importance score over 1 are represented as black slots and were considered as relevant. The ligands and TFs discussed in the results sections are highlighted in red. **l**, **m** Overlay of the spatial mapping of the RNF43 gene expression and the predicted TEAD1 TF activity with the pathologist’s tissue annotations in the ST-colon4_Tre sample. **n**, **o** Overlay of the spatial mapping of the DCN gene expression and the predicted ETV4 TF activity with the pathologist’s tissue annotations in the ST-liver4_Tre.

We first applied our deconvolution-based approach to profile this dataset (Fig. 6a). Major cell type proportions revealed a reduced tumor content of approximately 4% in ST-colon2_Unt, ST-colon3_Tre, and ST-liver3_Tre samples, in accordance with their histology. All samples, including the liver metastases, predominantly exhibited a CMS2 phenotype, with over 80% of tumor cells mapped to this subtype (Fig. 6b, c, Supplementary Fig. S20). In agreement with our previous results, CMS3 signatures were restricted to the non-neoplastic mucosa and CMS4 signals were minor and multifocally distributed. The CMS1 presence was almost negligible in these samples. Notably, substantial CMS2 and iCMS2 signals overlapped with the liver tumor histology, suggesting a conservation of the CMS phenotype in metastasis (Fig. 6d, Supplementary Figs. S21–S22). We further characterized these samples by analyzing the relative abundance of the different types of T cells, B cells, myeloid cells and the main stromal cells (Supplementary Fig. S23).

Next, we spatially mapped CRC-associated molecular features and assessed their correlation with the CMS cell abundance jointly in primary and hepatic metastatic tumors, focusing on the prevalent CMS2 subtype (Fig. 6e, f, Methods). As a result, we verified the activation of WNT and VEGF pathways in CMS2-rich regions and confirmed the activity of MYC and E2F4 transcription factors in CMS2 tumors (Fig. 6g, h, Supplementary Fig. S24a, b). Moreover, we noticed a link between the estimated CMS2 cells and the activity of the MAPK pathway and NR2C2 TF (Fig. 6i, j). This finding is consistent with our primary sample set (Fig. 2k, l) and of particular interest as their role in CMS2 tumors is not clearly defined.

We also used the external dataset to validate selected cell-to-cell communication processes previously identified, specifically the ligand-TF regulations. Using primary CRC tumors, we confirmed the modulation of JUN and TEAD family transcriptional activity by RNF43 expression, and the potential influence of DCN on ETV4 activity (Fig. 6k–m, Supplementary Fig. S24c, d). We also confirmed the potential downstream impact of the CXCL14 chemokine on MAF’s transcriptional activity (Fig. 6k, Supplementary Fig. S24e, f). Notably, we found that ETV4 and JUN’s transcriptional activity regulation by DCN and RNF43, respectively, was preserved in the liver metastatic samples (Fig. 6n, o, Supplementary Fig. S24g–i). These findings align with a recent study describing the protective role of DCN in hepatic metastasis of CRC^43^ and may provide new insights into the underlying molecular mechanisms.

Overall, the main findings of our study were indeed validated in an independent ST CRC dataset.

## Discussion

The clinical need for accurate CRC patient stratification led to the development of several gene expression-based classification systems, such as the CMS^5^ or the IMF^8^. The CMS classification system is broadly used and has helped to understand the different molecular mechanisms underlying CRC and disease prognosis^44^. Nevertheless, CMS intra-tumor heterogeneity hampers its clinical application, underlining the necessity of further characterizing the cellular composition and architecture of CRC and its microenvironment.

To complement our understanding of CRC CMS, we combined ST and scRNA-seq via cell type deconvolution, elucidating subtype-inherent transcriptomic and morphological features. This allowed us to map CMS1 and CMS2 tumor cells to neoplastic areas exhibiting distinct morphological features. In contrast, CMS3 signatures were confined to the non-neoplastic mucosa, which might be related to their normal-like expression patterns^5^. The EMT-associated CMS4 signals were minimal and overlapped with invasive tumor regions, in line with previous studies referring to CMS4 as a transcriptional state of stromal cells rather than tumor-like epithelial cells^10,45^. This reduced signal made it challenging to observe typical CMS4 molecular features such as TGFb pathway activation in our integrated analysis (Figs. 2l and 6e), though such features are evident in individual samples (Supplementary Fig. S25a). Across various samples, we observed a co-existence of the different subtypes in line with recent findings suggesting that CRC is more accurately represented by a transcriptomic continuum than by discrete subtypes^7^. Indeed, the bulk RNA-based classification of our analyzed samples emphasizes the significant influence of the surrounding tissue on tumor classification (Supplementary Fig. S25b, c). The S6_Rec patient samples illustrate this, with small tumor islands enveloped by large stroma bundles, leading to a CMS2 classification via deconvolution but a CMS4 assignment by CMScaller^46^. This morphology hampers the separation of the tumor components in bulk RNA-seq data, whereas ST can provide their detailed assessment. The CMS4 classification of stroma-rich tumors is in accordance with previous studies linking CMS4 signatures with marker genes of cancer-associated fibroblast and other stromal cells^47^. Similarly, the external ST-colon4_Tre sample, classified as CMS2 by deconvolution but CMS3 by CMScaller, raises concerns about the impact of non-neoplastic mucosa, which contains CMS3 signals, on bulk-based CMS classification systems.

Overall, our results underline the potential of ST in CRC characterization beyond bulk- or scRNA-seq, enabling the spatial correlation of morphological tumor, stroma and non-neoplastic tissue patterns with corresponding transcriptomic features. Nevertheless, limitations inherent to our deconvolution-based approach should be acknowledged. Firstly, the choice of the scRNA-seq reference can significantly impact the deconvolution results. We compared the results yielded by two similarly annotated reference datasets^6^ in Supplementary Note 1. The overall results were highly comparable, but some discrepancies were observed for particular cell types, e.g. CMS1 tumor cells. Factors such as the differences in the genetic background between both cohorts could contribute to these discrepancies. Secondly, and regardless of the used reference, the deconvolution partially failed to map stromal cells on their expected anatomical location, especially in the S3_Col_R sample. This can be attributed to the absence of specific stromal cell types in the reference or due to a decrease in deconvolution sensitivity in regions with lower transcripts per spot, as a result from tissue properties or technical variabilities (Supplementary Fig. S26). Finally, the current size of 10x VISIUM spots makes region-specific assignment challenging, as seen in samples from the S6_Rec patient, where its unique morphology complicates pure tumor spot annotation. This may cause interpatient tumor expression differences due to residual stromal cells. It possibly explains the elevated FOXM1 transcriptional activity and the mixed CMS2 and stromal-related signatures in cluster 6, unique to S6_Rec in our TF activity-based clustering (Figs. 3d and 4a–d).

We also explored the ability of ST to scrutinize ligand-receptor interactions at the tumor-stroma interface, which might trigger signaling pathways critical for tumor progression. Our results encompass a range of novel and well-known tumor growth-inhibiting as well as -activating signatures, such as the potential regulation of the ETV4 transcriptional activity by DCN or the PLAU-PLAUR ligand-receptor interaction. While these predictions may guide the identification of potential therapeutic targets, they require further investigation as our methodology of spatially modeling TF activity based on ligand gene expression may not necessarily reflect direct causal regulations. Along the same line, the ligand-receptor analysis could also capture indirect gene expression associations. For instance, we consistently predicted the RNF43-FZD2 interaction targeting stromal cell populations in both ST and scRNA-seq data. However, this interaction is mostly reported to occur in the intracellular domain of RNF43 in tumor cells^48^, with few studies reporting a potential extracellular interaction^49^.

To support our key findings, we used an independent ST CRC dataset. Interestingly, our deconvolution approach delineated the primary, but also the metastatic carcinomas, as CMS2. In these liver tumors, we captured the CMS2 main molecular features and preserved cell communication events as the modulation of the transcriptional activity of ETV4 by DCN. This suggested that the CMS2 phenotype was largely retained after migration of the primary CRC cells to sites of metastasis.

In conclusion, our study illustrates the value of integrating ST and scRNA-seq in analyzing CRC and its CMS, providing insights into spatial cellular organization within tumors and their TME. Although the small patient cohort limits the scope of our study, we envision that our proof-of-concept work demonstrates ST’s potential to inform patient-specific treatment strategies. More refined patient stratification could be achieved by jointly considering cell composition, spatial distribution and morphological features. In addition, understanding intra-tumor spatial heterogeneity can unveil anatomically restricted or region-specific progression-related processes, fueling the development of novel therapies, such as targeted or combination treatments. As ST technologies evolve in resolution, affordability, and clinical validation, we anticipate its application to larger CRC cohorts, paving the way towards personalized oncology.

## Methods

### Collection of CRC samples

Human CRC tissues (<8 months storage) and annotated data were obtained and experimental procedures were performed within the framework of the non-profit foundation HTCR (Human Tissue and Cell Research) Foundation^50^. This includes written informed consent from all donors and the approval by the ethics commission of the Faculty of Medicine in the Ludwig Maximilian University of Munich (Number 025-12) and the Bavarian State Medical Association (Number 11142). Sampling and handling of any patient material was performed in accordance with the ethical principles of the Declaration of Helsinki. Tissues were cut on a Cryostat (CryoStar NX70, Thermo Scientific) at 10 um. A pathologist performed quality and comparability assessment of fresh-frozen material using a hematoxylin-eosin (H&E) stained slide.

### Sample preparation

RNA from all samples was extracted using the Arcturus® PicoPure® RNA Isolation Kit (Applied Biosystems™, KIT0204). For cell lysis, a 10 um section of the sample was resuspended in a 200 ul extraction buffer. Total RNA was extracted following the instructions of the manual. RNA integrity number (RIN) was assessed using the 2100 Bioanalyzer system (Agilent Technologies, Inc.) with an Agilent RNA 6000 Pico Kit (Agilent Technologies, Inc., 5067-1513). Samples with RIN above 7.0 were used.

Tissue optimization was carried out according to the manufacturer’s instructions (VISIUM Spatial Tissue Optimization User Guide_RevC). Image acquisition was performed on the Hamamatsu NanoZoomer S 360 C13220 series at 40x magnification and the coverslip was removed afterwards by immersing the slide in a 3x Saline-Sodium Citrate buffer. The stained tissue sections were permeabilized using a time course to test for the optimal permeabilization time. After performing a fluorescent cDNA synthesis, the tissue was removed. Finally, the fluorescent cDNA was imaged using a Zeiss Axio Scan.Z1 with a Plan Apochromat 20×/0.8 M objective, an ET-Gold FISH filter (ex 538–551 nm/em 556–560 nm) and 100 ms exposure time.

For the gene expression analysis, 10 um thick sections of the samples were placed with a random distribution over four chilled 10x Genomics VISIUM Gene Expression slides containing four capture areas each. The sections were similarly stained with H&E and subsequently imaged as described above. To release the mRNA, the sections were permeabilized for 30 min as defined by tissue optimization. For further processing, the cDNA was amplified according to the manufacturer’s protocol (CG000239_VisiumSpatialGeneExpression_UserGuide_RevC). Double indexed libraries were prepared. The libraries were quality controlled using a 2100 Bioanalyzer system with Agilent High Sensitivity DNA Kit (Agilent Technologies, Inc., 5067-4626) and quantified with Qubit™ 1X dsDNA HS Assay Kit (Invitrogen, Q33230) on a Qubit 4 Fluorometer (Invitrogen, Q33238). The libraries were loaded onto the NovaSeq 6000 (Illumina) at a concentration of 250 pM. A NovaSeq S1 v 1.5 or SP v 1.5 Reagent Kit (100 cycles) (Illumina, 20028319 and 20028401) was used. For paired end-dual indexed sequencing, the following read protocol was used: read 1: 28 cycles; i7 index read: 10 cycles; i5 index read: 10 cycles; and read 2: 90 cycles. All libraries were sequenced at a minimum of 50000 reads per covered spot.

Raw sequencing data were demultiplexed using the *mkfastq* function from Space Ranger (v. 1.2.0). Demultiplexed data were mapped to the human reference GRCh38 with *spaceranger count*. Spots under tissue folds, artifacts and at the tissue boundary were manually removed using the 10X Loupe browser (v. 5.1.0).

### Histopathological annotations and spot categorization

H&E stained tissue sections were annotated by the pathologist using QuPath software (v. 0.2.3)^51^. Spot categorization was performed by the pathologist using the 10X Loupe browser (v. 5.1.0). Categories and corresponding criteria are listed in Supplementary Table S5.

### Grading of CMS signatures

Grading of CMS signatures in the tumor tissue was performed semi-quantitatively according to the number of spots with positive signature and the percentage of positive cells per spot. This grading was done in an individual replicate per patient (S1_Cec_Rep1, S2_Col_R_Rep1, S3_Col_R_Rep1, S4_Col_Sig_Rep1, S5_Rec_Rep1, S6_Rec_Rep2 and S7_Rec/Sig_Rep1) according to the scheme detailed in Supplementary Table S6.

### ST data pre-processing

We used the Seurat^52^, Scanpy^53^ and SingleCellExperiment^54^ packages to load the output of the Space Ranger pipeline and process the ST data. We evaluated the quality of the ST data by determining the average number of reads, UMIs and genes per spot covered by tissue and compared it with those from spots non covered by tissue. We found substandard quality for the S1_Cec_Rep2 sample as revealed by its low numbers of unique molecular identifier (UMI) counts and genes in spots covered by tissue (Supplementary Fig. S1). Consequently, this sample was either treated carefully or excluded from integrative analysis. For each individual sample, we filtered out spots for which the number of UMI counts detected were below 500 or above 45000. In addition, spots containing a fraction of more than 0.5 mitochondrial genes were not considered in the analysis. We normalized the UMI counts from the remaining spots using SCTransform^55^.

### Sample integration, batch correction and dimensionality reduction

To jointly represent the CRC samples in the same low dimensional space (UMAP embedding), correct from batch effects and integrate samples and technical replicates for downstream analysis, we used Harmony^56^. We ran Harmony with default parameters allowing a maximum number of 20 interactions (*max.iter.harmony* = 20) and correcting per individual samples. Of note, Harmony was either applied to batch-correct for all the spots derived from all the samples or to batch-correct only the tumor annotated spots from a subset of samples (CMS2 tumor samples).

### Deconvolution of the ST datasets

ST datasets derived from 10x Genomics VISIUM technology currently lack single cell resolution. Therefore, the gene expression values detected per spot originate from a variable number of different cells, i.e. every spot can be considered as a mini-bulk RNAseq dataset. Consequently, a deconvolution approach is required to estimate the different cell types and their proportions across spots.

To this end, we used the recently proposed Cell2Location (v 0.0.5)^18^ method. Cell2location first creates gene expression signatures of cell types from a scRNA-seq reference. We adopted as scRNA-Seq reference a comprehensive dataset from a recent publication exploring the cellular landscape of the different CRC subtypes and their microenvironment^6^. The annotations from the original publication at the cell subtype level (Supplementary Table S1) were used to generate the signature using the *run_regression* function with the following parameters: *n_epochs* = *100, minibatch_size* *=* *1024, learning_rate* *=* *0.01* and *train_proportion* = 0.9. These signatures are subsequently used to assess cell type abundances in the ST data using the *run_cell2location* with *selection_specificity* = 0.20. This parameter determines the number of genes used to establish the signature per cell type (Supplementary Table S1). Additional parameters were set as follows: *n_iter* = *40000, cells_per_spot* = *8, factors_per_spot* = *9, combs_per_spot: 5, mean* = *1/2 and sd* = *1/4*.

### Consistency of deconvolution results between technical replicates

To evaluate the consistency of the deconvolution between technical replicates, we batch-corrected their transcriptomic profiles using Harmony^56^ as described above. Then, we clustered the Harmony embeddings using the Louvain algorithm as encoded in the *FindClusters* function from the Seurat package. We chose a series of large resolution parameters (ranging from 1 to 2 increasing by 0.1 steps) to obtain fine-grain clusters that can match with anatomical regions displaying similar cell type distribution patterns across replicates. Finally, we computed the mean number of UMIs estimated by Cell2Location per cell type and cluster, and applied Pearson’s correlation to evaluate their similarity between technical replicates.

### Enrichment/depletion of cell types in different anatomical regions

The enrichment (depletion) in the abundance of the deconvolution-estimated cell types in different pathologist-assigned tissue categories was assessed following a similar procedure to be one described in Andersson et al.^11^. Briefly, the estimated cell type proportions per spot were 10 000 times randomly shuffled with respect to their spatial location. Then, we computed the average cell type proportions per permutation and tissue type. The mean value of differences between the real and the permuted average proportions divided by the standard deviation of these differences was used as the enrichment score for the different tissue categories.

### Pathway activity

We estimated pathway activity per spot and at subspot resolution (see section Clustering and enhanced gene expression at the sub spot level) using PROGENy^57^. PROGENy computes pathway activity by accounting for the expression of genes which are more responsive to perturbations on those pathways. The PROGENy model comprises 14 pathways, namely: Wnt, VEGF, Trail, TNFα, TGFβ, PI3K, p53, NFkB, MAPK, JAK/STAT, Hypoxia, Estrogen, Androgen and EGFR. In our setup, we ran PROGENy using the top 500 most responsive genes per pathway.

In addition, we also computed pathway activities in pseudo-bulk generated from our ST samples (see section Pseudo-bulk generation). We again used the top 500 most responsive genes per pathway. In this case, we set the *scale* parameter to *TRUE* to allow direct comparison of pathway activities between samples.

### Transcription factor activity

We computed TF activity per spot using the Viper^58^ algorithm coupled with regulons extracted from DoRothEA^59^. In DoRothEA, every TF–target interaction is assigned a confidence score based on the reliability of its source, which ranges from A (most reliable) to E (least reliable). In this study, we selected interactions with confidence scores A, B and C and computed the activity for TFs with at least four different targets expressed per spot.

The activity profiles of the different TFs were additionally used to cluster the spots from our four CMS2 tumor samples. To do so, the TF activity scores from these samples were first merged and subsequently scaled and centered. Then, the standard procedure to compute clustering using the Seurat package was followed. Briefly, we computed a Principal Component Analysis (PCA) dimensionality reduction on the scaled TF activities per spot followed by the computation of the 20 nearest neighbors. Finally, we applied the Louvain algorithm with a resolution parameter of 0.5 to group the spots into different clusters according to their TF activity profile. We identified TF with a differential activity profile among the different clusters using Receiver Operating Characteristic (ROC) analysis as implemented in the Seurat’s *FindAllMarkers* function. We only considered TF whose activity was computed in at least 25% of the spots per cluster and with a log_2_ fold-change greater than 1.

Of note, we used the same procedure to compute TF activity per cell on the scRNA-seq dataset from Lee et al.^6^.

### Canonical correlation analysis

We used the *cc* function from the CCA package^60^ to compute canonical correlation between the cell type proportions per spot and pathway or TF activity per spot. This canonical correlation analysis was first performed for every individual CRC sample. To capture global correlations across samples, we performed an integrative analysis by merging spots coming from all the different samples (excluding S1_Cec_Rep2) into matrices and computing the canonical correlation on them.

### Selection of tumor surrounding spots

We applied the *GetTissueCoordinates* function from the Seurat package to get the spatial coordinates of the spots in the different CRC samples. We subsequently computed the Euclidean distance between every pair of spots. Finally, we selected as tumor-surrounding-spots those lying within a distance smaller or equal to 2 from a tumor annotated spot. Spots fulfilling these criteria but annotated as tumors were discarded.

### Pseudo-bulk generation

We generated pseudo-bulk from the ST samples using the *sumCountsAcrossCells* function from the Scater package^61^. Here, counts were normalized by the total number of reads (counts per million normalization). We used the *filterByExpr* function from the edgeR package^62^ to filter out genes with less than 50 counts per sample.

### Definition of different anatomical regions in tumor annotated spots

The distance between every tumor annotated spot and non-tumor annotated spots was calculated as described in section Selection of tumor surrounding spots. We then defined the different tumor anatomical regions for the S2_Col_R_Rep1 sample based on the following criteria:

- *Peripheral Tumor*: tumor spots in direct contact with at least a non-tumor annotated spot. Their Euclidean distance to a non-tumor annotated spot is smaller than 2.
- *Central Tumor*: tumor spots in the most solid and internal region of the tumor. Their Euclidean distance to a non-tumor annotated spot is greater than 2.5.
- *Intermediary Tumor*: tumor spots that we consider as a transition region between the inner and outer tumor. Their Euclidean distance to a non-tumor annotated spot is greater or equal to 2 and smaller than 2.5.

### Clustering and enhanced gene expression at the sub spot level

We applied BayesSpace^63^ to cluster at the subspot level and increase the gene expression resolution of our CMS2 tumor annotated spots in the S5_Rec_Rep1 sample. To do so, BayesSpace uses the neighborhood structure in spatial transcriptomic data. Of note, the preprocessing of the ST raw data was conducted following the recommendations of BayesSpace authors. This procedure is slightly different from the one described in previous sections. Briefly, the ST data was processed using the SingleCellExperiment package and raw counts were log normalized using the *logNormCounts* function from the Scuttle package^61^. Then, the Scran^64^ package was used to model the variance of the log-expression profiles for each gene and select the 2000 most variable genes. We performed a PCA using the Scater^61^ package.

Using BayesSpace, we subsequently computed the spatial clustering and the enhanced clustering with default parameters, excepting the *jitter_scale* parameter which was set to 3. Finally, we enhanced the gene expression of all the genes expressed in the considered spots using the *enhanceFeatures* function with default parameters.

### Differential gene expression analysis

The CMS2 tumor regions extracted from the different samples were integrated into the same Seurat^52^ object. We used the Wilcoxon Rank Sum test to identify differentially expressed genes between the groups of spots coming from different patients as implemented in the Seurat’s *FindAllMarkers* function. We set a log_2_ fold-change threshold of 0.25 and only positive markers were retrieved. Some specific criteria were followed for the analyses conducted in section 2.3:

- To describe inter-patient heterogeneity, the differential gene expression analysis was performed between the different patients (two replicates per patient considered). We filtered results by only considering genes that are overexpressed in tumor annotated spots versus non-tumor annotated spots. To do so, we took advantage of the pathologist’s annotations and used the Seurat’s *FindMarkers* with the same parameters described above for the *FindAllMarkers* function. Ribosomal and mitochondrial genes were removed due to the fact that they can be overrepresented in tumor necrotic regions.
- To describe intra-tumor heterogeneity, the differential expression analysis was carried out between the different anatomical regions of the tumor in the S2_Col_R_Rep1 sample (see section Definition of different anatomical regions in tumor annotated spots) with no further considerations.
- Another differential gene expression analysis was conducted on the enhanced gene expression between the different enhanced clusters generated by BayesSpace (see section Clustering and enhanced gene expression at the sub spot level) on the S5_Rec_Rep1 sample. We selected for further analysis genes with an adjusted *p*-value smaller than 0.01 in the Wilcoxon Rank Sum test. Ribosomal and mitochondrial genes were excluded from the analysis.

### Gene set overrepresentation analysis

Differentially expressed genes were subsequently used for gene set overrepresentation analysis using the Hallmark annotations from MSigDB^65^. The Hallmark gene sets contain 50 well-defined biological states or processes. We used the *enricher* function from the clusterProfiler^66^ package to carry out the analysis. We set a minimal size of the genes annotated for testing to five, excepting for the analysis between different patients where it was set to three. Background genes were adjusted accordingly to the global set of genes expressed in the different contexts.

### Ligand modulation of TF activity

As a first step and taking as reference the TF activity-based clustering, we selected ligands which are overexpressed in the tumor and TME with respect to the other anatomical regions across all our CRC samples. To do so, we applied the Seurat’s *FindMarkers* function with a log_2_ fold-change threshold of 0.5 and only positive markers were retrieved. We matched our set of overexpressed genes against the set of proteins annotated as ligands in the Omnipath^67^ database. Additionally, we filtered out ligands that are not detected in at least 10% of the tumor and TME spots in every individual sample.

In the second place, we chose TFs with a higher differential activity profile in the TME regions across all the samples according to the clustering approach described in section Transcription factor activity. In particular, we selected those TFs that are considered as markers of the TME cluster when using the Seurat’s *FindAllMarkers* function (AUC ≥ 0.75).

We then applied Misty^31^ to investigate the potential effect of the expression of the selected ligands in modulating the transcriptional activity of the chosen TFs. Specifically, we created an intrinsic view (*intraview*) describing ligand gene expression and a local niche view (*juxtaview*) using TF activity with a *neighbor.thr* = *2* aiming at capturing effects in the direct neighborhood of each spot. This criteria is based on the fact that many cancer relevant ligands are membrane bound and that the majority of secreted ligands cannot travel long distances. Following this approach, Misty was first individually applied to every sample. Then, the individual results were collected and aggregated using Misty’s *collect_results* function in order to obtain the most robust common signals across samples. Ligand-TF associations with an aggregated importance greater than 1 were considered for further analysis. Of note, when running Misty on the external dataset, the ST-colon3-Tre and ST-liver3-Tre samples were excluded from the analysis due to their reduced tumor content.

### Prediction of ligand-receptor interactions

We used LIANA^68^ to estimate the most likely ligand-receptor interactions between the different spatial clusters defined by their TF activity profiles. It is to note that the interactions were computed for every pair of clusters, but for subsequent analysis and visualization we focused on the interactions between the clusters labeled as 0 (Tumor) and 1 (TME). LIANA computes an aggregated score for every potential ligand-receptor interaction based on the results of different methods. In our particular case, we ran LIANA with default settings and used OmniPath^67^ as a source of prior knowledge in human ligand-receptor interactions. For further analysis, we considered interactions involving Misty’s predicted ligands with an aggregated rank smaller than 0.01, as this value can be seen as analogous to a *p*-value^69^. We also ran LIANA on the scRNA-seq dataset from Lee et al.^6^ using the same procedure.

### Inference of signaling networks

We used a network-based approach to infer the most likely signaling cascades linking LIANA’s predicted ligand-receptor interactions to their targeted TFs according to Misty’s predictions. To do so, we first built an intra-cellular signaling network by retrieving protein-protein interactions from Omnipath^67^. Then, for every ligand, we selected their predicted receptors and targeted TFs. We subsequently connected every receptor to every corresponding TF by selecting the shortest path between them in the signaling network. All the resultant shortest paths were merged into a network together with the previously predicted ligand-receptor interactions. Finally, for every gene in the predicted network, we computed its average expression in the TME cluster, as defined by TF activity profiles (see section Transcription factor activity), across all the CMS2 samples. Cytoscape^70^ was utilized for the visualization of the network.

### Metagenes/module scores

We computed module scores for different sets of genes using the Seurat’s *AddModuleScore* function. We detail below the particular gene sets used:

- The list of up-regulated genes in iCMS2 and iCMS3, as well as, the markers involved in gastric metaplasia were extracted from the study where the IMF classification system was introduced^8^.
- The list of genes associated with tubular adenomas or with sessile serrated lesions were extracted from Chen et al.^71^.
- We fetched the crypt bottom and upper markers from Kosinski et al.^72^.
- We retrieved a list of genes linked to metastatic processes from CancerSEA^73^.

### Prediction of microsatellite status

We inferred microsatellite instability status by running Microsatellite instability Absolute single sample Predictor (MAP)^74^ on pseudo-bulk generated from our ST samples (see section Pseudo-bulk generation). They were classified as microsatellite instable (MSI) or microsatellite stable (MSS).

### Reporting summary

Further information on research design is available in the Nature Research Reporting Summary linked to this article.

### Supplementary information

Supplementary Materials

Reporting Summary

## Data Availability

The output of Space Ranger, including processed count data matrices and histological images, for the ST data generated in this study is available at 10.5281/zenodo.7551712. In addition, this repository also contains the spot categorization made by the pathologist. The processed scRNA-seq and metadata used for the deconvolution and for further characterization of the cell communication processes are available via the GEO database under the accession codes GSE132465 and GSE144735^6^. The processed data from the external ST CRC dataset used to support our findings was downloaded from http://www.cancerdiversity.asia/scCRLM^14^.

## Code availability

The scripts containing all the code used to generate the results presented in this study are available at https://github.com/alberto-valdeolivas/ST_CRC_CMS. Their associated notebooks containing additional results and information about the versions of the different packages used are available at 10.5281/zenodo.7440182. Finally, Intermediary object files to reproduce the analysis are available at 10.5281/zenodo.7551712.
