# Supplementary material for: Profiling the heterogeneity of colorectal cancer consensus molecular subtypes using spatial transcriptomics
Source: NPJ Precis Oncol. 2024 Jan 10;8:10. doi: 10.1038/s41698-023-00488-4 (PMC10781769; doi:10.1038/s41698-023-00488-4)
Supplement: Supplementary file 1 — Supplementary Materials [file 41698_2023_488_MOESM1_ESM.pdf]

Supplementary Materials

The present file contains the supplementary materials associated with the "*Profiling the Heterogeneity of Colorectal Cancer Consensus Molecular Subtypes using Spatial Transcriptomics*" publication. In particular, it contains the following sections:

1. *Supplementary Figures*
2. *Supplementary Tables*
3. *Supplementary Note 1*

1. Supplementary Figures

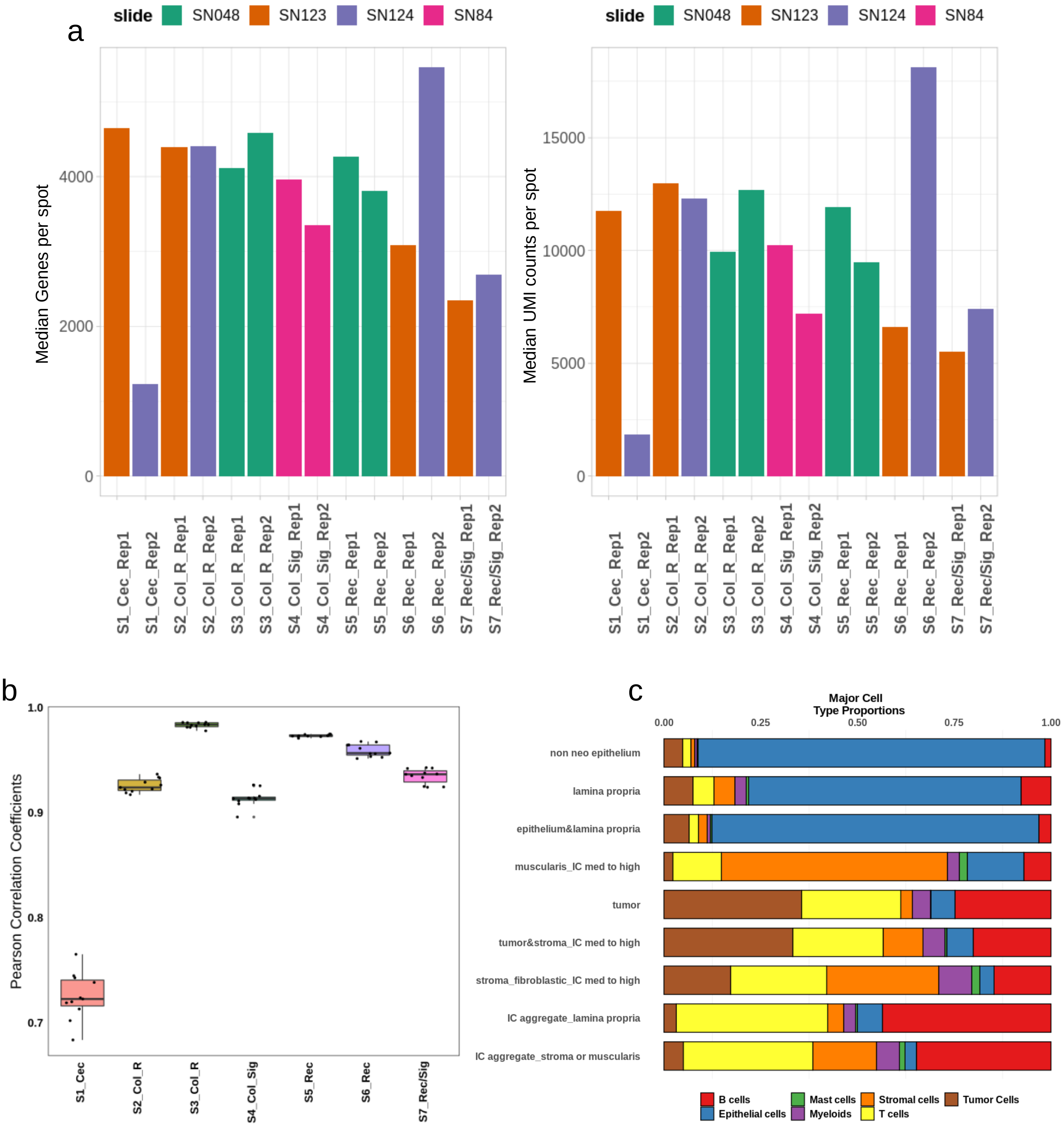

Supplementary Figure S1: *a) Median number of genes and UMI counts per spot in our set of CRC samples. The bar color indicates the samples that were processed on the same gene expression slide. Note the reduced number of genes and UMI counts in the S1_Cec_Rep2 sample, which was considered of substandard quality; b) Pearson's correlation coefficients of the cell subtype abundance in small anatomical regions of variable size that were considered equivalent between technical replicates for all the patients (Methods); c) Proportions of major cell classes as estimated by the results of the deconvolution approach in the different anatomical regions annotated by the pathologists.*

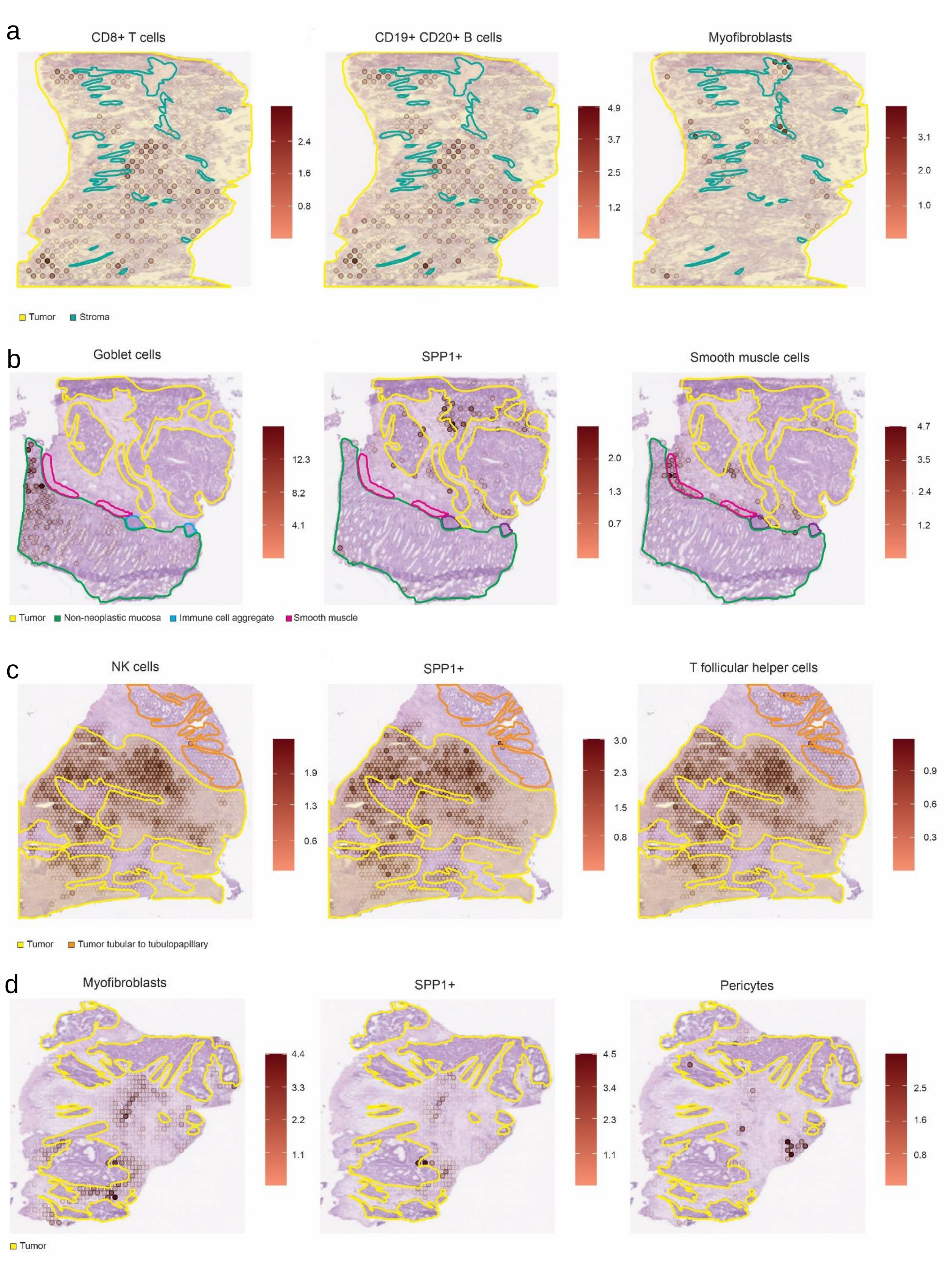

Supplementary Figure S2: *Spatial mapping of the predicted number of cells of different types according to the deconvolution results, overlaying with the pathologist's tissue annotations for the samples: a) S1_Cec_Rep1, b) S2_Col_R_Rep1, c) S3_Col_R_Rep1 and d) S4_Col_Sig_Rep2.*

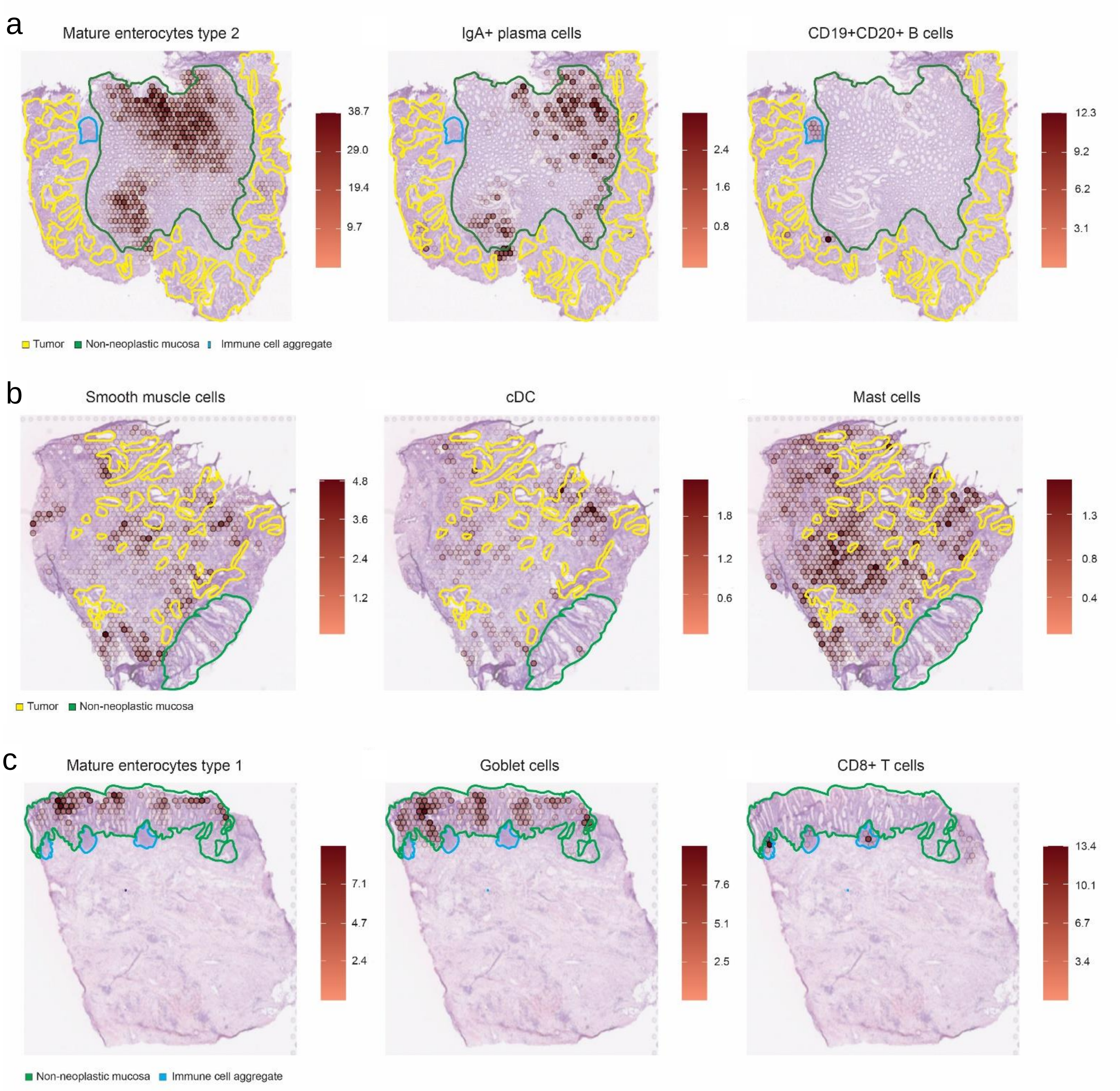

Supplementary Figure S3: *Spatial mapping of the predicted number of cells of different types according to the deconvolution results, overlaying with the pathologist's tissue annotations for the samples: a) S5_Rec_Rep1, b) S6_Rec_Rep2, and c) S7_Rec/Sig_Rep1.*

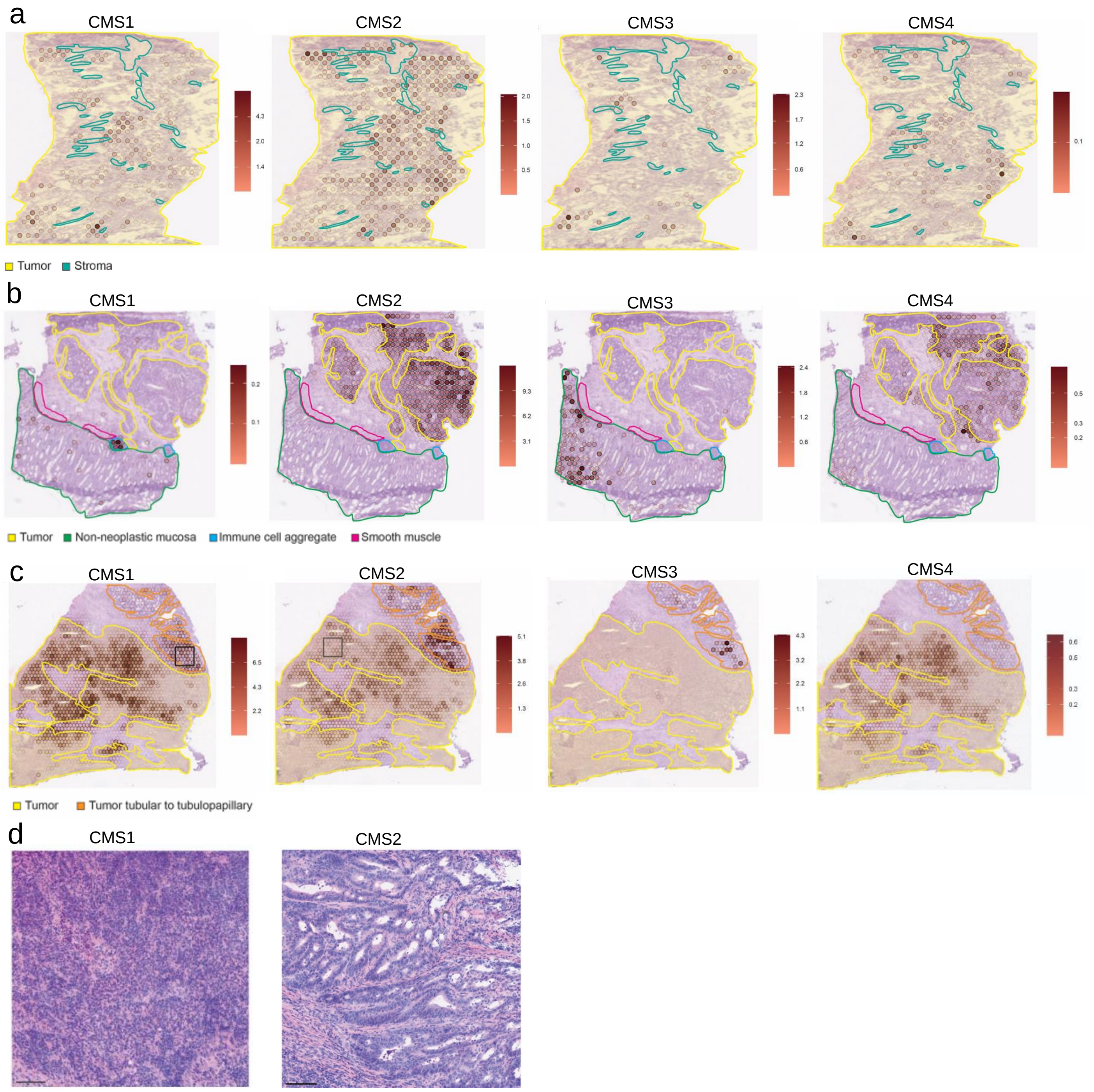

Supplementary Figure S4: *Spatial mapping of the predicted number of tumor cells of the different Consensus Molecular Subtypes (CMS) of CRC according to the deconvolution results, overlaying with the pathologist's tissue annotations for the samples: a) S1_Cec_Rep1, b) S2_Col_R_Rep1 and c) S3_Col_R_Rep1; d) Hematoxylin and eosin (H&E) staining of sample S3_Col_R_Rep1 reveals morphological features for the different tumor anatomical regions: CMS1-predominant areas display a diffuse growth pattern; whereas CMS2-predominant regions are delineated from the former and show a tubular to tubulopapillary growth pattern. Scale bar 100 μm.*

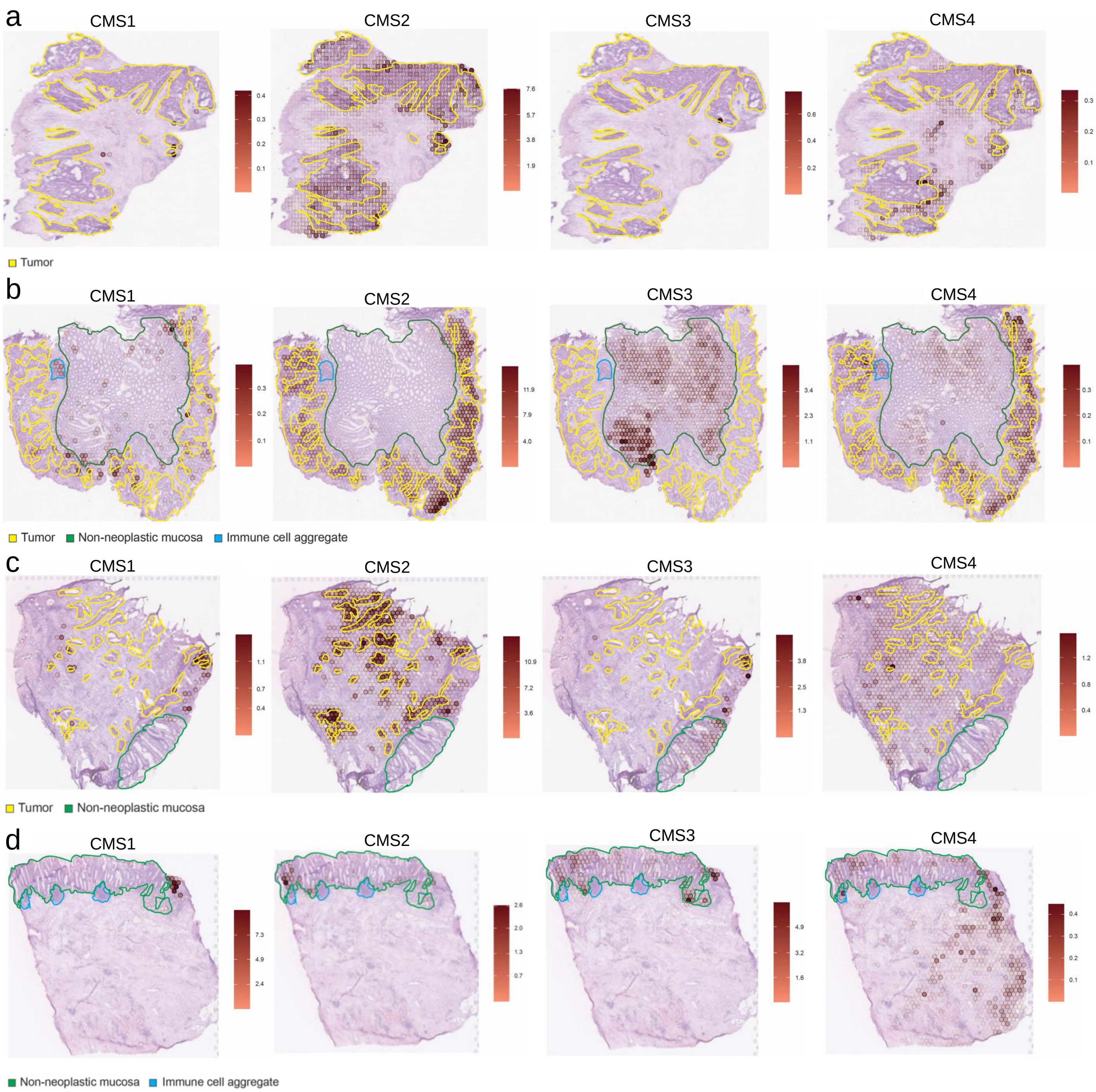

Supplementary Figure S5: *Spatial mapping of the predicted number of tumor cells of the different Consensus Molecular Subtypes (CMS) of CRC according to the deconvolution results, overlaying with the pathologist's tissue annotations for the samples: a) S4_Col_Sig_Rep2, b) S5_Rec_Rep1, c) S6_Rec_Rep2, and d) S7_Rec/Sig_Rep1*

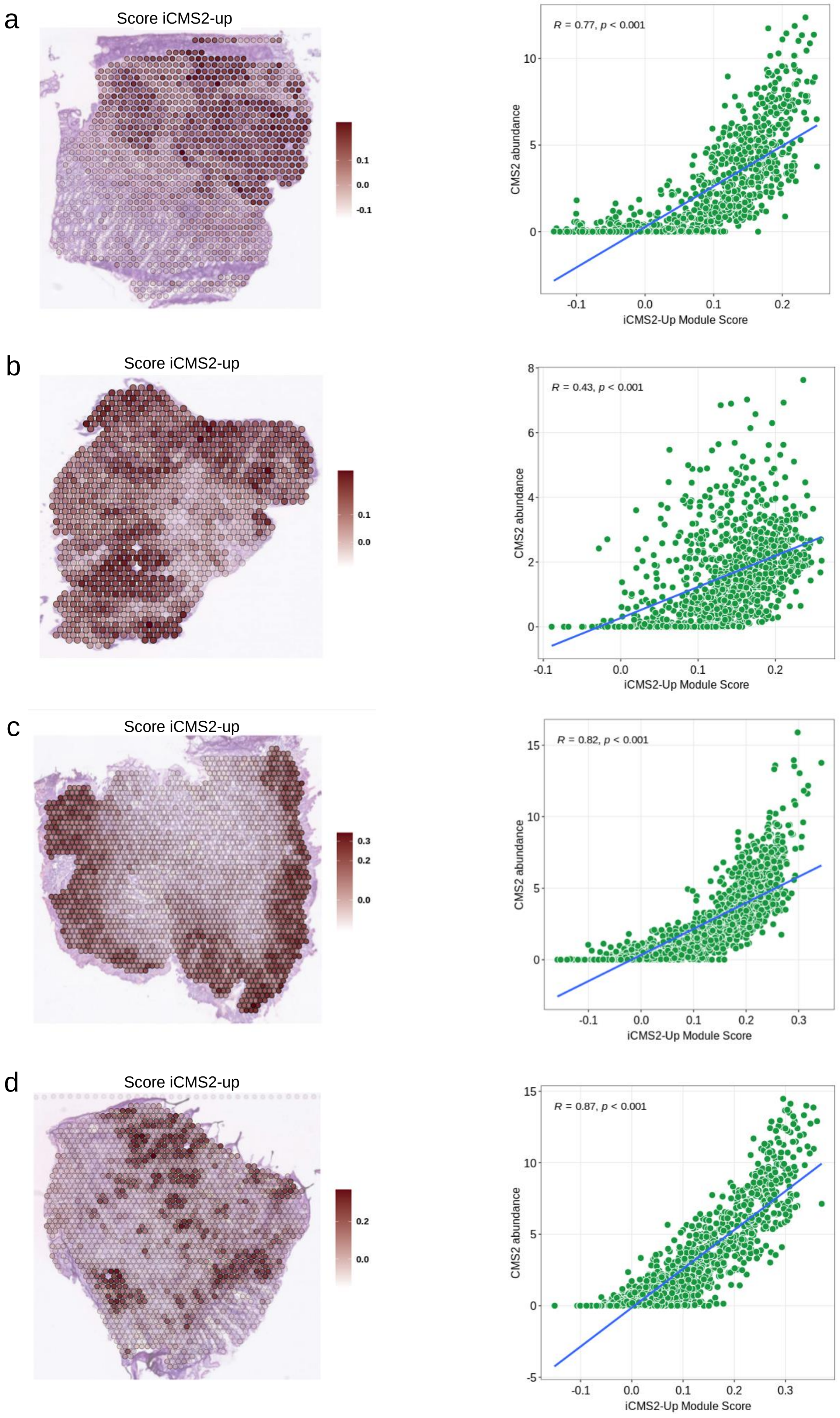
a
Score iCMS2-up
0.1
0.0
-0.1
R = 0.77, p < 0.001
CMS2 abundance
iCMS2-Up Module Score
b
Score iCMS2-up
0.1
0.0
R = 0.43, p < 0.001
CMS2 abundance
iCMS2-Up Module Score
c
Score iCMS2-up
0.3
0.2
0.0
R = 0.82, p < 0.001
CMS2 abundance
iCMS2-Up Module Score
d
Score iCMS2-up
0.2
0.0
R = 0.87, p < 0.001
CMS2 abundance
iCMS2-Up Module Score

Supplementary Figure S6: *Spatial mapping of the iCMS2-upregulated module score and the Pearson's correlation between this score and the deconvolution-predicted CMS2 abundance for the samples: a) S2_Col_R_Rep1, b) S4_Col_Sig_Rep2, c) S5_Rec_Rep1, and d) S6_Rec_Rep2.*

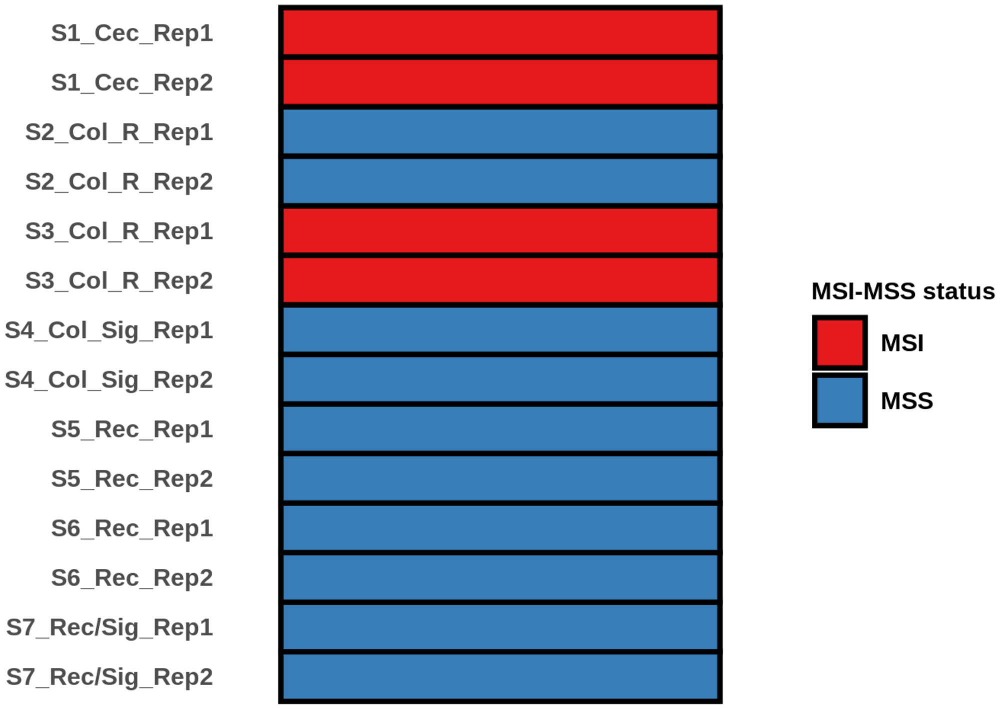

Supplementary Figure S7: *Microsatellite instability status resulting from applying MAP (Microsatellite instability Absolute single sample Predictor) on pseudo-bulk RNA-seq generated by pooling together all the spots for each of our CRC samples. MSI: microsatellite instable; MSS: microsatellite stable.*

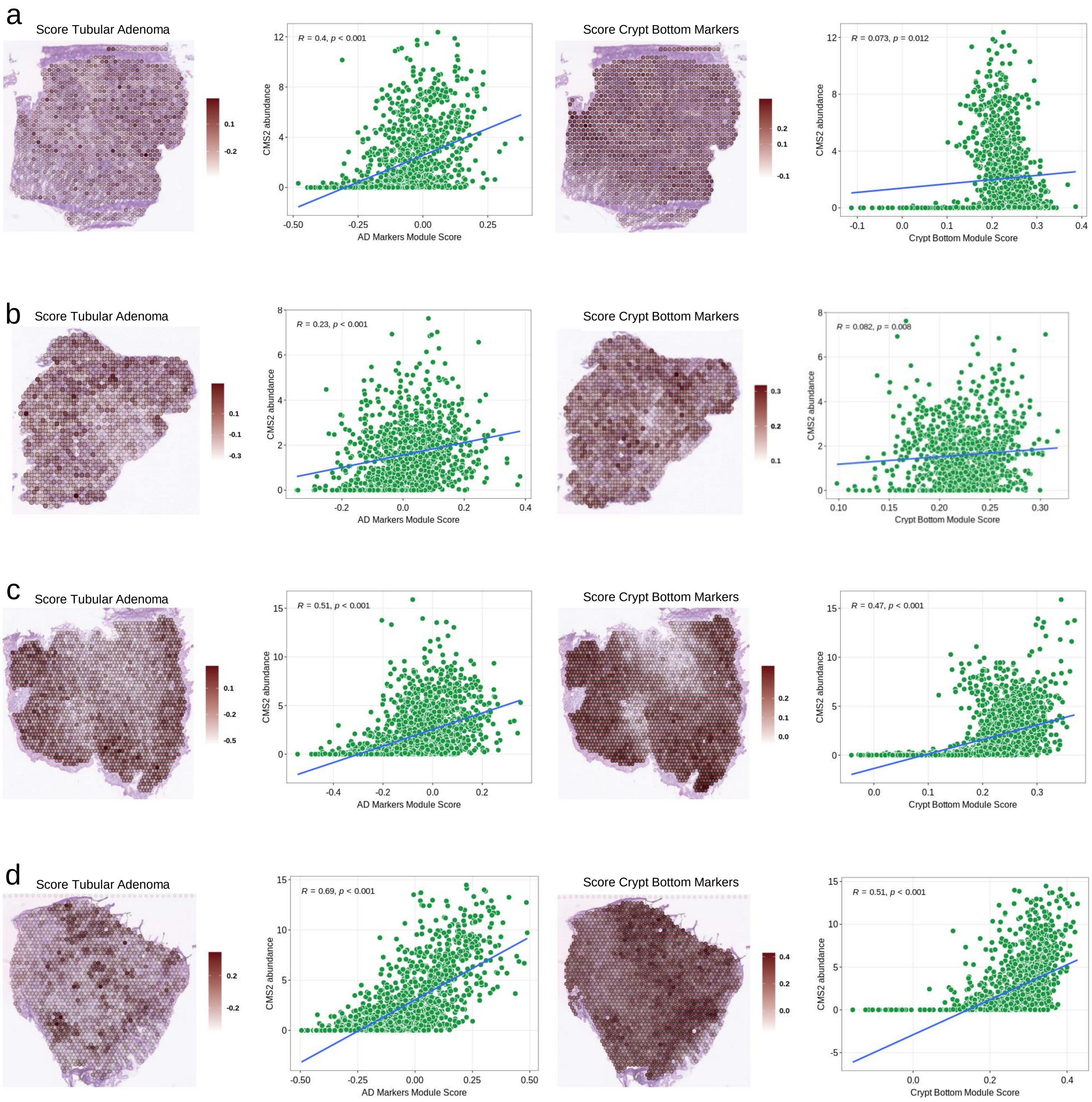

Supplementary Figure S8: *Spatial mapping of the tubular adenoma markers and crypt bottom markers module score and the Pearson's correlation between these scores and the deconvolution-predicted CMS2 abundance for the samples: a) S2_Col_R_Rep1, b) S4_Col_Sig_Rep2, c) S5_Rec_Rep1, and d) S6_Rec_Rep2. AD: Tubular Adenoma.*

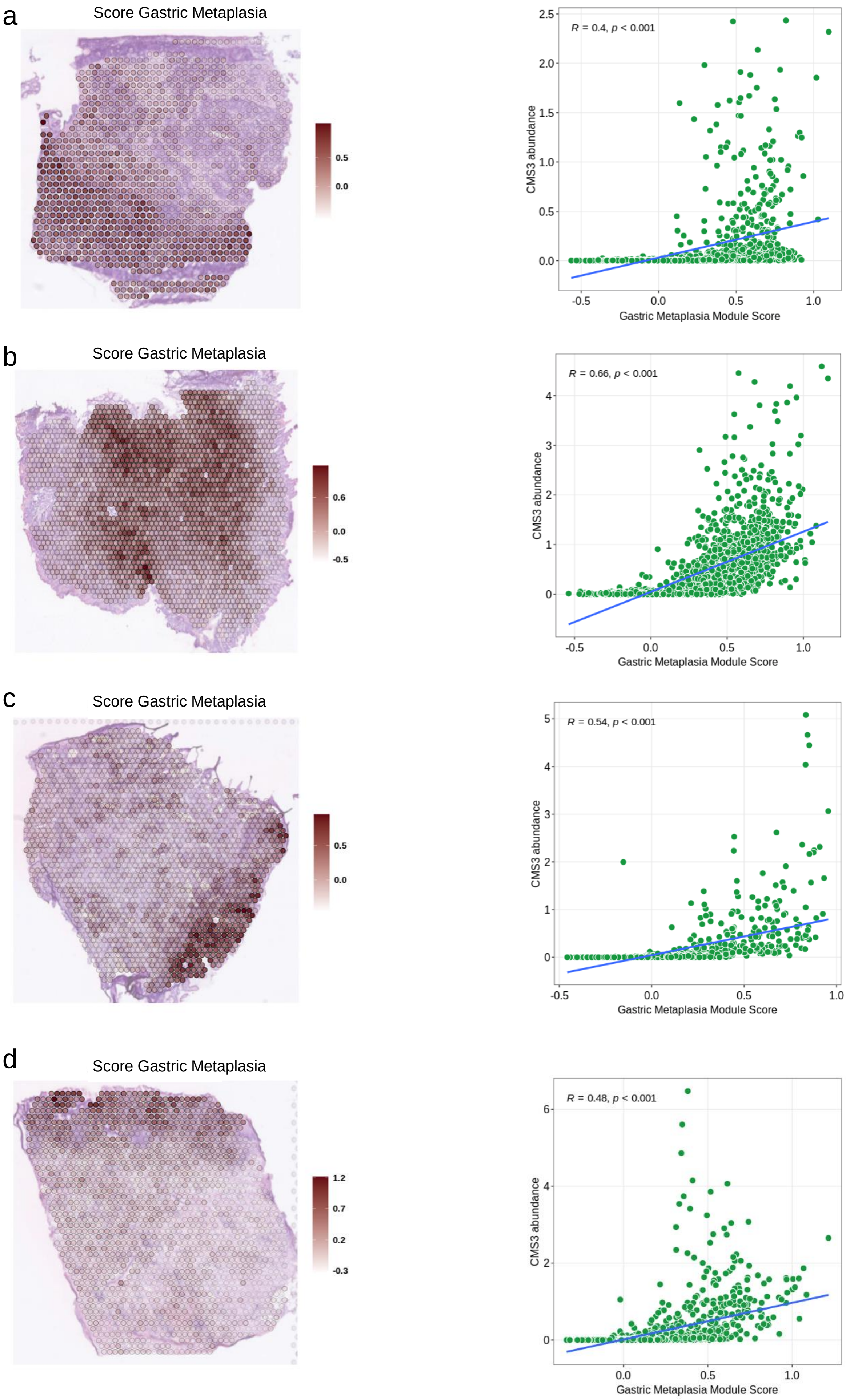
a
Score Gastric Metaplasia
0.5
0.0
R = 0.4, p < 0.001
CMS3 abundance
Gastric Metaplasia Module Score
b
Score Gastric Metaplasia
0.6
0.0
-0.5
R = 0.66, p < 0.001
CMS3 abundance
Gastric Metaplasia Module Score
c
Score Gastric Metaplasia
0.5
0.0
R = 0.54, p < 0.001
CMS3 abundance
Gastric Metaplasia Module Score
d
Score Gastric Metaplasia
1.2
0.7
0.2
-0.3
R = 0.48, p < 0.001
CMS3 abundance
Gastric Metaplasia Module Score

Supplementary Figure S9: *Spatial mapping of the gastric metaplasia module score and the Pearson's correlation between this score and the deconvolution-predicted CMS3 abundance for the samples: a) S2_Col_R_Rep1, b) S5_Rec_Rep1, c) S6_Rec_Rep2 and d) S7_Rec/Sig_Rep1.*

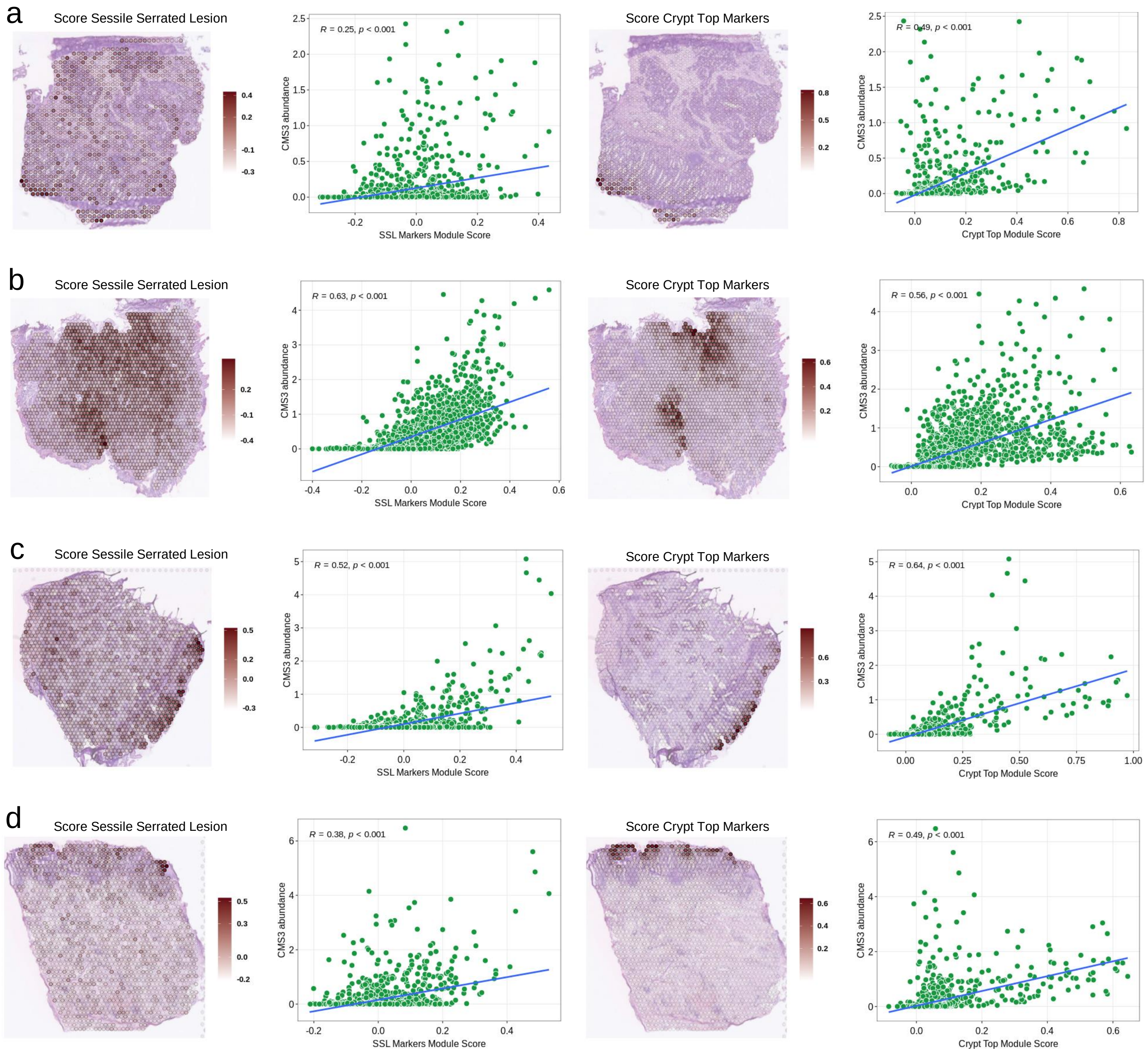

Supplementary Figure S10: *Spatial mapping of the sessile serrated lesion markers and crypt top markers module score and the Pearson's correlation between these scores and the deconvolution-predicted CMS3 abundance for the samples: a) S2_Col_R_Rep1, b) S5_Rec_Rep1, c) S6_Rec_Rep2, and d) S7_Rec/Sig_Rep1. SSL: Sessile serrated lesion.*

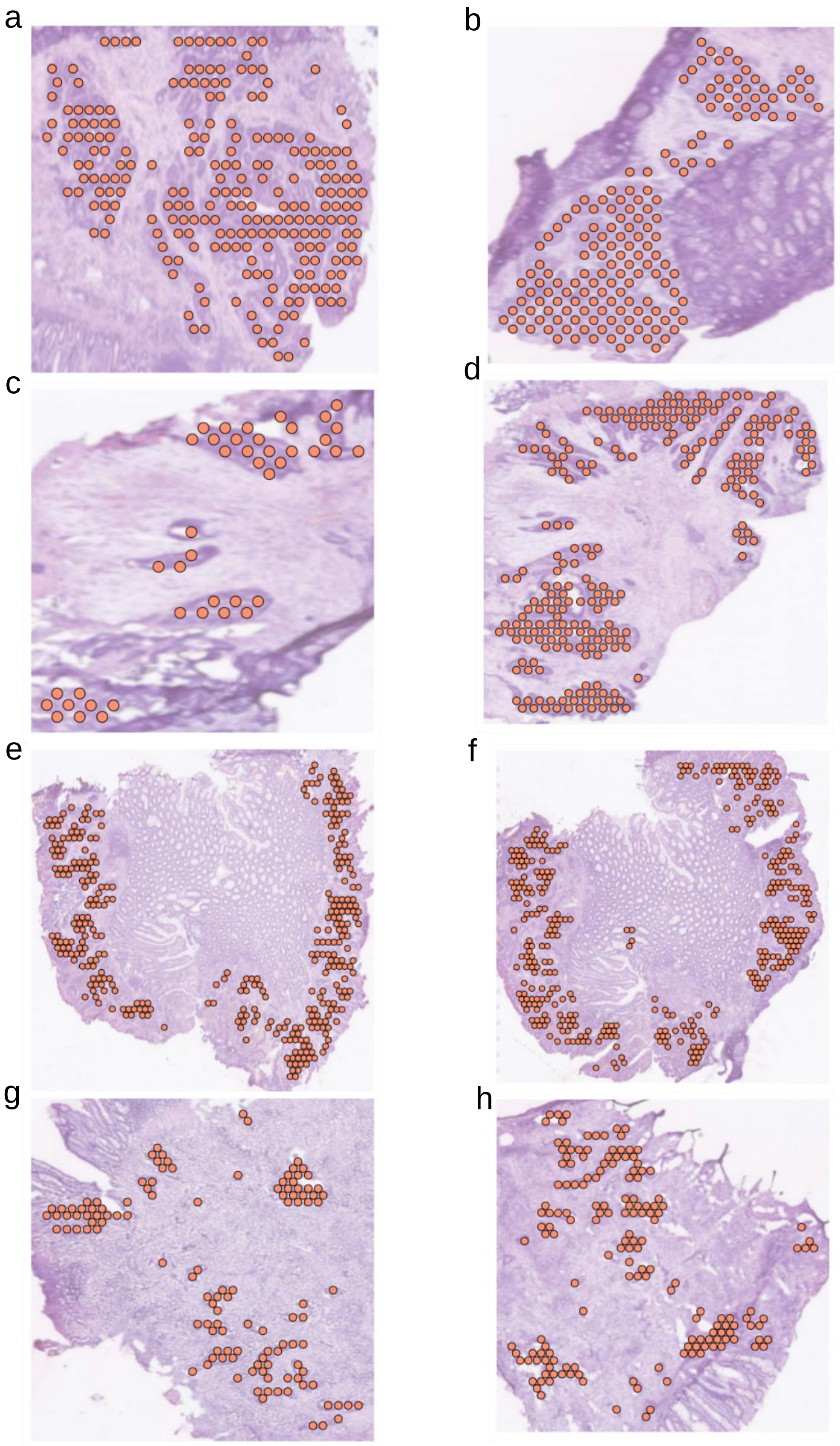

Supplementary Figure S11: *Spots annotated as tumor by the pathologist for the CMS2 tumor samples, a) S2_Col_R_Rep1, b) S2_Col_R_Rep2, c) S4_Col_Sig_Rep1, d) S4_Col_Sig_Rep2, e) S5_Rec_Rep1, f) S5_Rec_Rep2, g) S6_Rec_Rep1, and h) S6_Rec_Rep2.*

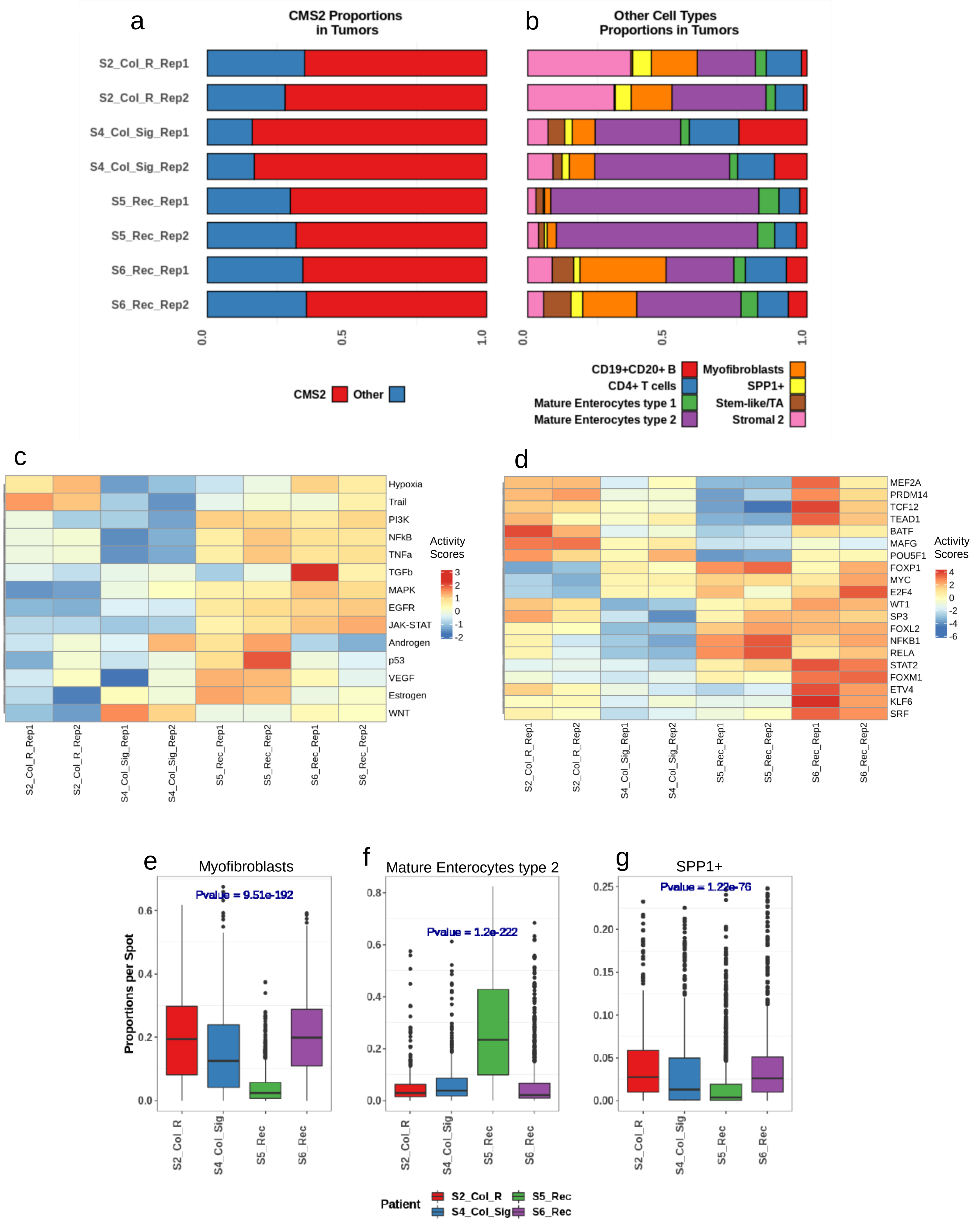

a
CMS2 Proportions in Tumors
S2_Col_R_Rep1
S2_Col_R_Rep2
S4_Col_Sig_Rep1
S4_Col_Sig_Rep2
S5_Rec_Rep1
S5_Rec_Rep2
S6_Rec_Rep1
S6_Rec_Rep2
0.0
0.5
1.0
CMS2
Other
b
Other Cell Types Proportions in Tumors
CD19+CD20+ B
CD4+ T cells
Mature Enterocytes type 1
Mature Enterocytes type 2
Myofibroblasts
SPP1+
Stem-like/TA
Stromal 2
c
Hypoxia
Trail
PI3K
NFkB
TNFa
TGFb
MAPK
EGFR
JAK-STAT
Androgen
p53
VEGF
Estrogen
WNT
Activity Scores
d
MEF2A
PRDM14
TCF12
TEAD1
BATF
MAFG
POU5F1
FOXP1
MYC
E2F4
WT1
SP3
FOXL2
NFKB1
RELA
STAT2
FOXM1
ETV4
KLF6
SRF
Activity Scores
e
Myofibroblasts
Pvalue = 9.51e-192
Proportions per Spot
f
Mature Enterocytes type 2
Pvalue = 1.2e-222
g
SPP1+
Pvalue = 1.22e-76
S2_Col_R
S4_Col_Sig
S5_Rec
S6_Rec
Patient

Supplementary Figure S12: *Cell type proportions in the tumor-annotated spots per sample as estimated by the results of the deconvolution: a) CMS2 proportions as compared to all the remaining cell types considered in the study, and b) proportions of the most relevant cell types within CMS2 tumors excluding CMS2 cells. Differential c) pathway and d) TF activity computed on pseudo-bulk RNA-seq generated from the tumor-annotated spots for the different CMS2 samples. e) myofibroblast, f) mature enterocytes type 2, and g) SPP1+ macrophages proportions per spot in the tumor neighborhood of the different patients (both replicates considered) as estimated by the results of the deconvolution approach. A Kruskal-Wallis statistical test was performed to assess whether the cell type proportions for the different samples originated from the same distribution (p-value).*

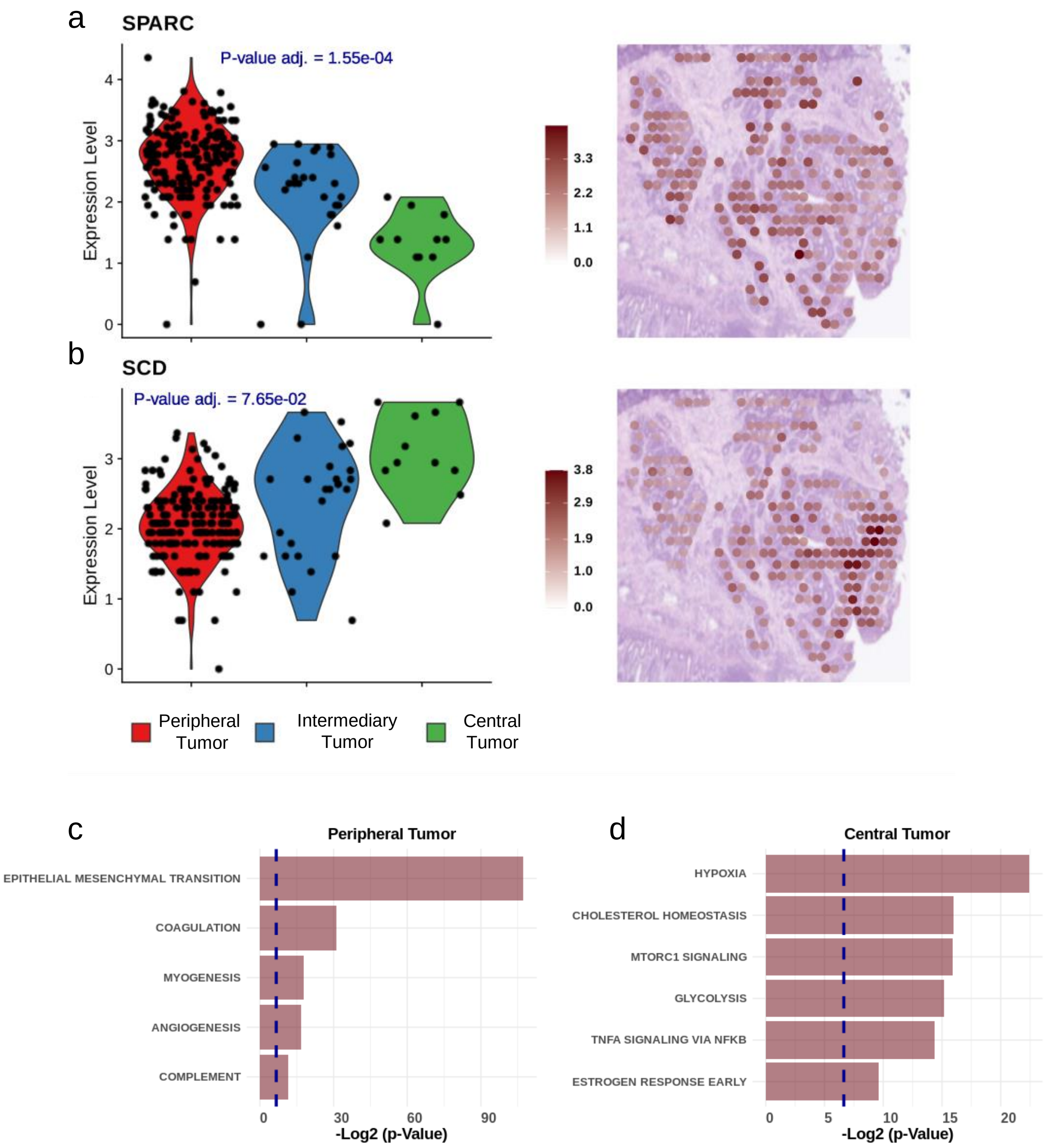

Supplementary Figure S13: *Gene expression gradients of a) SPARC and b) SCD in the different anatomical regions of tumor-annotated spots in the S2_Col_R_Rep1 sample. A Wilcoxon rank sum test was conducted to assess the significance of the gene expression variation (p-value adjusted). c,d) Significant enrichment results (p-value<0.01) on the differentially expressed genes between the different anatomical regions of the CMS2 tumor in sample S2_Col_R_Rep1.*

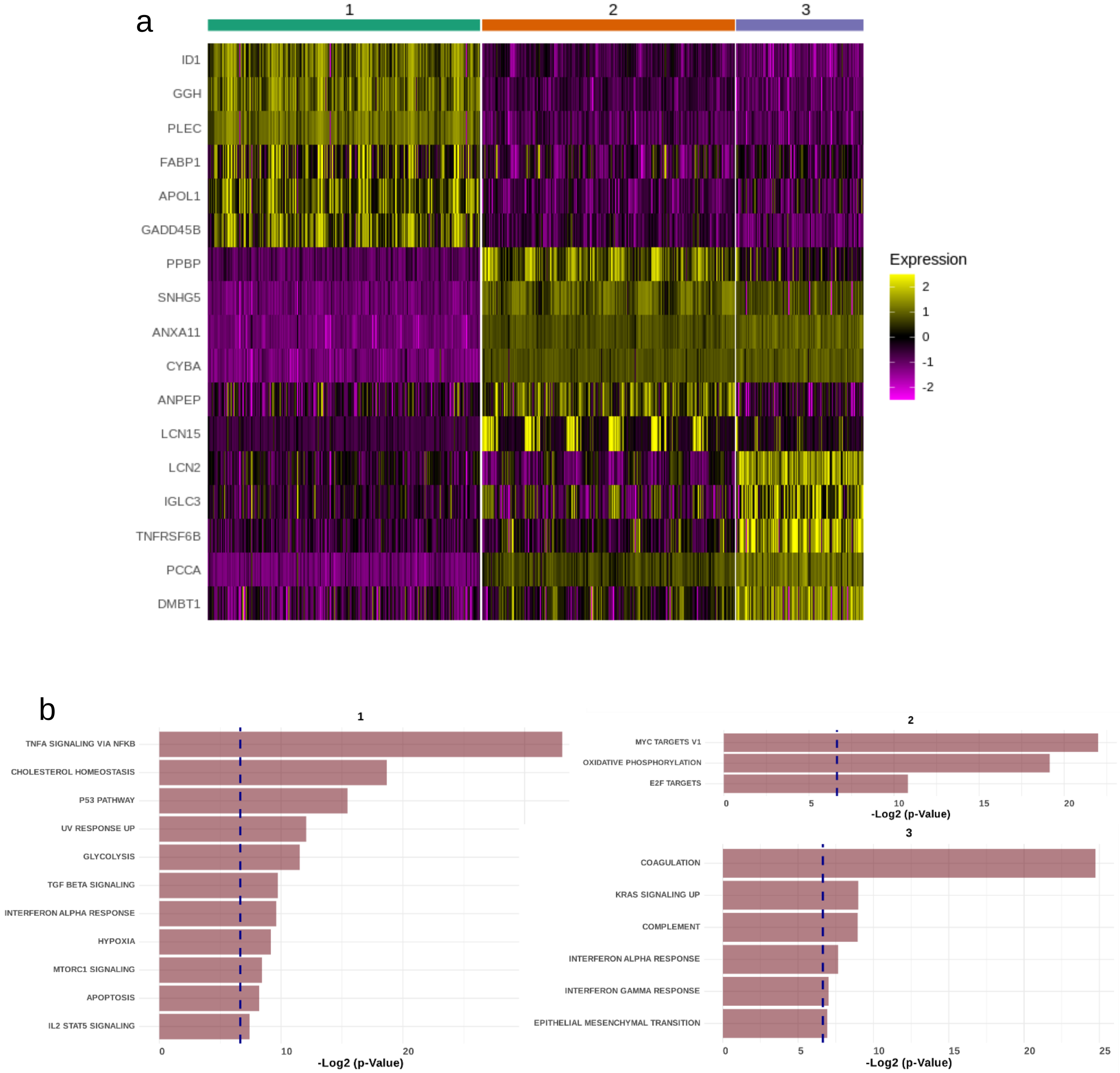

Supplementary Figure S14: *a) Heatmap displaying the differentially expressed genes with a larger fold-change per group between the different subclusters within the CMS2 tumor-annotated spots in the S5_Rec_Rep1 sample. For visualization purposes, a maximum of 6 markers are displayed per group; b) Significant enrichment results (p-value<0.01) on all the differentially expressed genes between the aforementioned subclusters.*

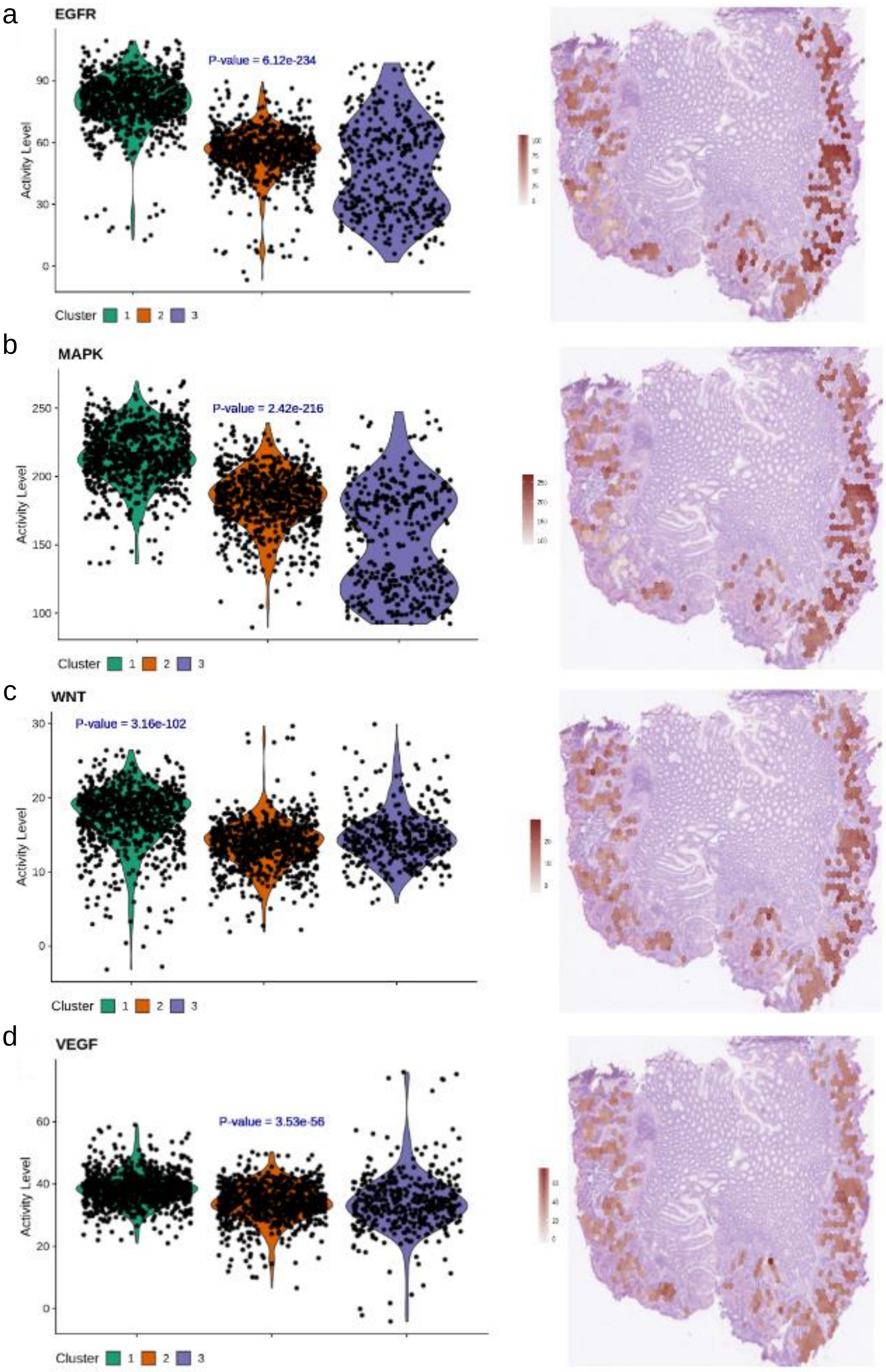
a
EGFR
P-value = 6.12e-234
Activity Level
90
60
30
0
Cluster 1 2 3
b
MAPK
P-value = 2.42e-216
Activity Level
250
200
150
100
Cluster 1 2 3
c
WNT
P-value = 3.16e-102
Activity Level
30
20
10
0
Cluster 1 2 3
d
VEGF
P-value = 3.53e-56
Activity Level
60
40
20
0
Cluster 1 2 3

Supplementary Figure S15: *Pathway activities at the enhanced subspot resolution. Per cluster violin plots and spatial distribution for the following pathways: a) EGFR, b) MAPK, c) WNT and d) VEGF in the S5_Rec_Rep1 sample. A Kruskal-Wallis statistical test was performed in each case to assess whether the pathway activities in the different subclusters originated from the same distribution (p-value).*

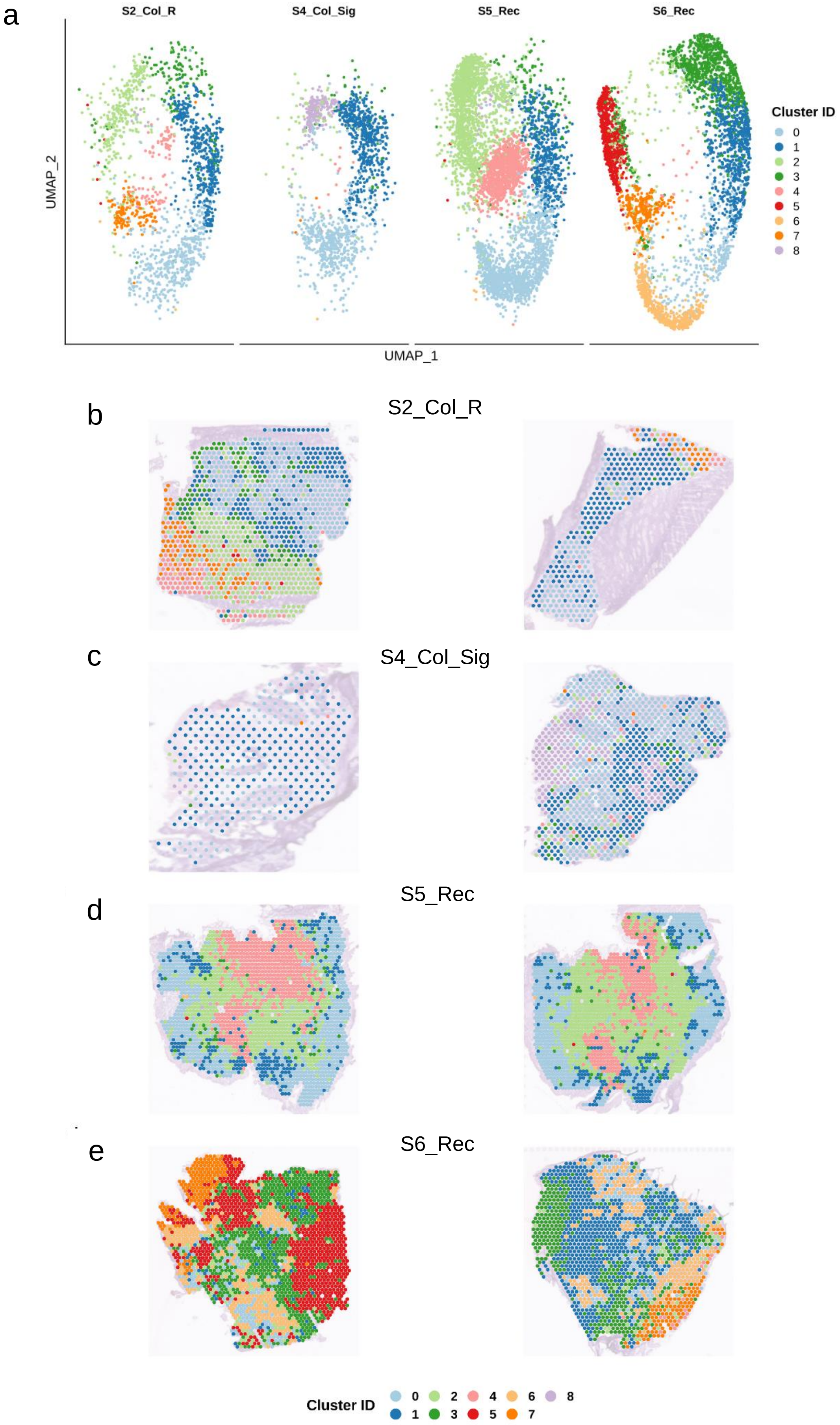
a
S2_Col_R
S4_Col_Sig
S5_Rec
S6_Rec
UMAP_2
UMAP_1
Cluster ID
0
1
2
3
4
5
6
7
8
b
S2_Col_R
c
S4_Col_Sig
d
S5_Rec
e
S6_Rec
Cluster ID
0 2 4 6 8
1 3 5 7

Supplementary Figure S16: *a) UMAP embedding of the TF activity profiles for our set of CMS2 samples split by patient with the spots colored by cluster identifiers. Spatial arrangement of these clusters in the samples: b)S2_Col_R, c) S4_Col_Sig, d)S5_Rec, and e) S6_Rec. Both replicates are displayed per patient.*

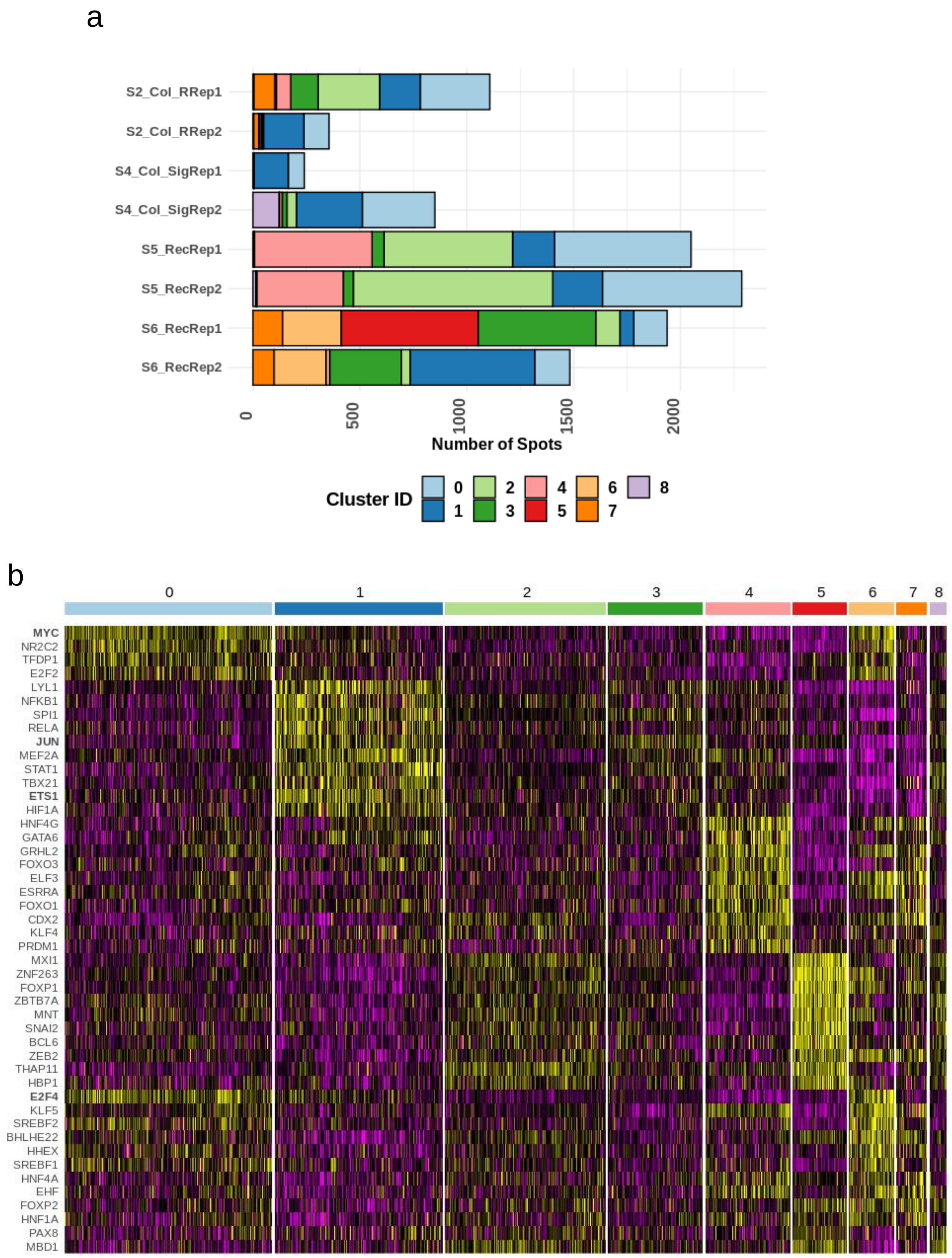

Supplementary Figure S17: *a) Number of spots per sample and per cluster identifier according to the results of the clustering based on TF activity profiles; b) Most differentially active TF between those different clusters. We display TF with an average Log2 fold-change > 1.25 and a maximum of 10 TFs per cluster. Bold characters were used to highlight the name of TFs referred to in the main text.*

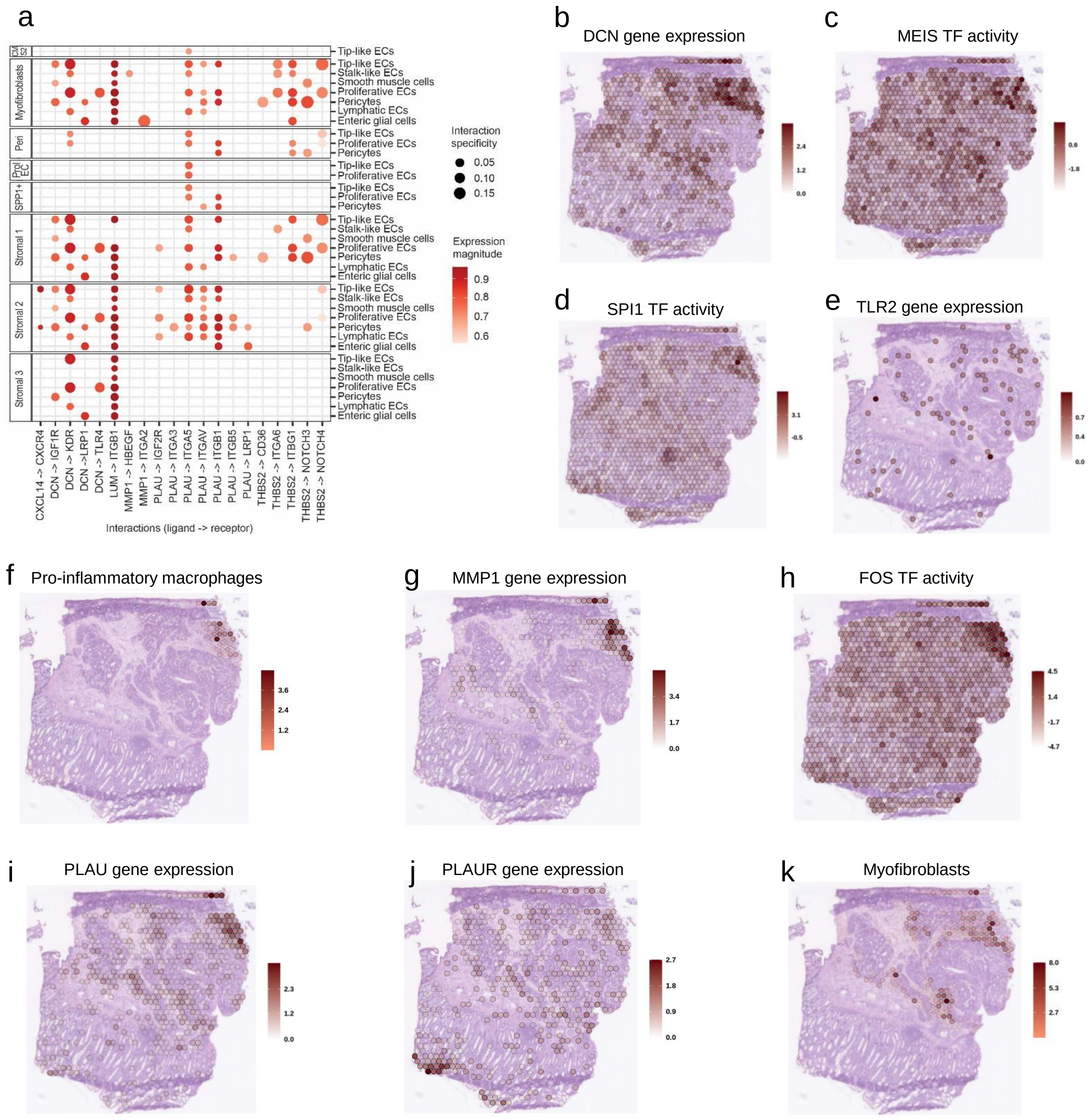

Supplementary Figure S18: *a) Ligand-receptor interactions between the different cell types derived from scRNA-seq dataset from Lee et al. that overlap with the interactions predicted in our ST data. The left panel shows the source of the interaction (ligands) and the right the target (receptors), which in this case are the minor stromal cell populations. Spatial mapping of: b) DCN gene expression, c) MEIS1 TF activity, d) SPI1 TF activity, e) TLR2 gene expression, f) estimated number of pro-inflammatory macrophages, g) MMP1 gene expression, h) FOS TF activity, i) PLAU gene expression, j) PLAUR gene expression, and k) estimated number of myofibroblasts in the S2_Col_R_Rep1 sample.*

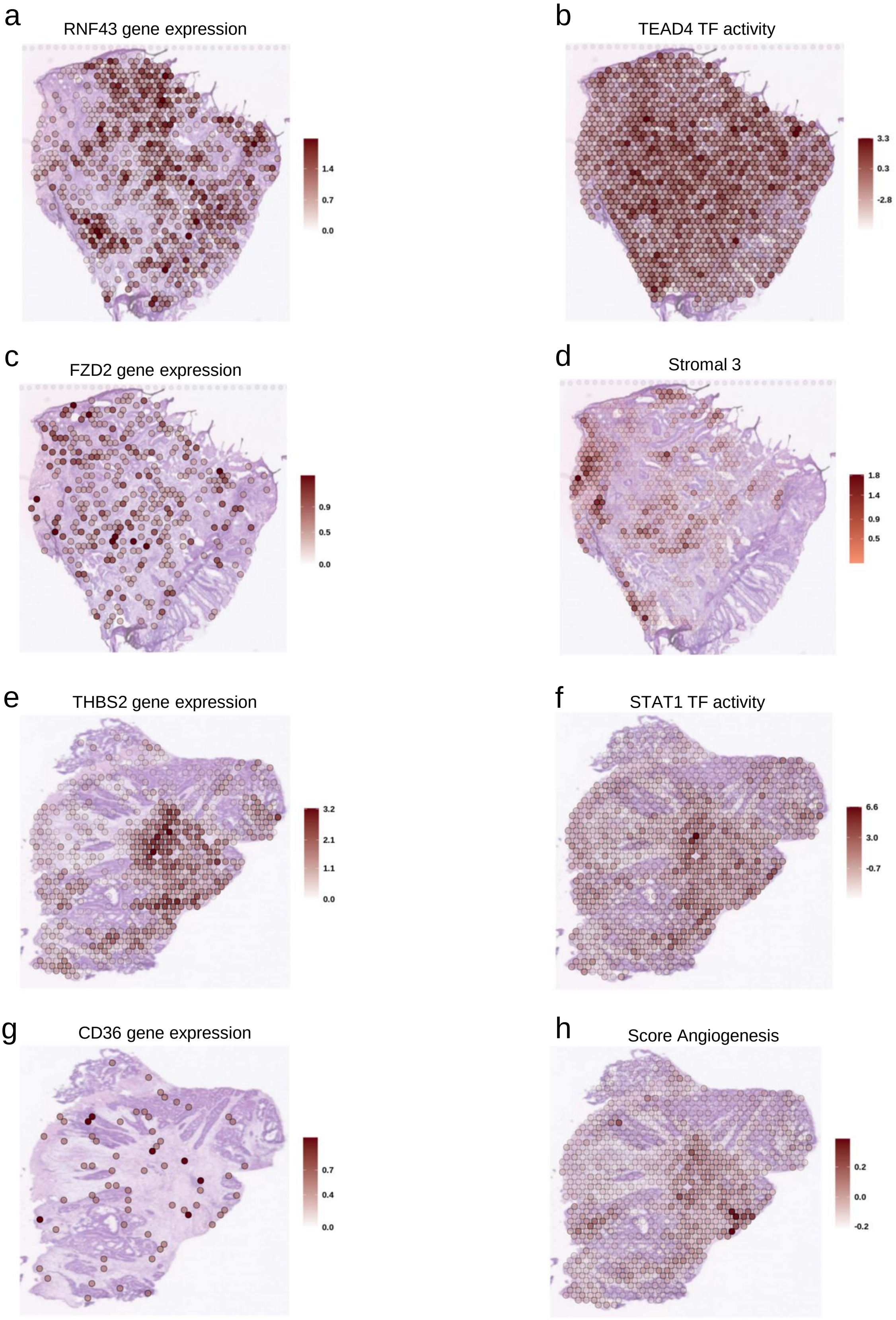

Supplementary Figure S19: *Spatial mapping of: a) RNF43 gene expression, b) TEAD4 TF activity, c) FZD2 gene expression, and d) estimated stromal 3 cell abundance in the S6_Rec_Rep2 sample. Spatial mapping of: e) THBS2 gene expression, f) STAT1 TF activity, g) CD36 gene expression, and h) the angiogenesis score in the S4_Col_Sig_Rep2 sample.*

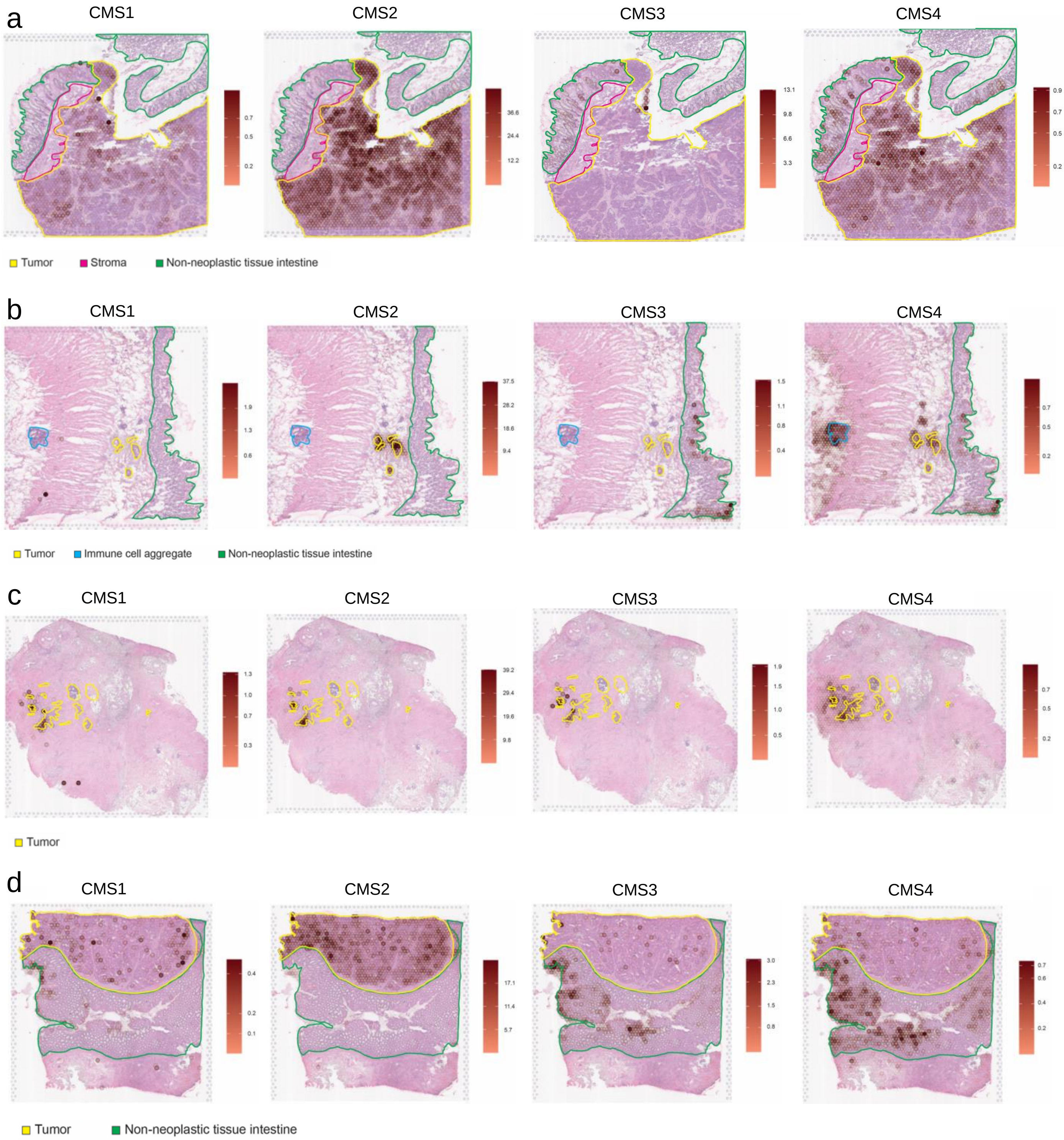

Supplementary Figure S20: *Spatial mapping of the predicted number of tumor cells of the different Consensus Molecular Subtypes (CMS) of CRC according to the deconvolution results, overlaying with the pathologist's tissue annotations for the samples: a) ST-colon1_Unt, b) ST-colon2_Unt, c) ST-colon3_Tre, and d) ST-colon4_Tre.*

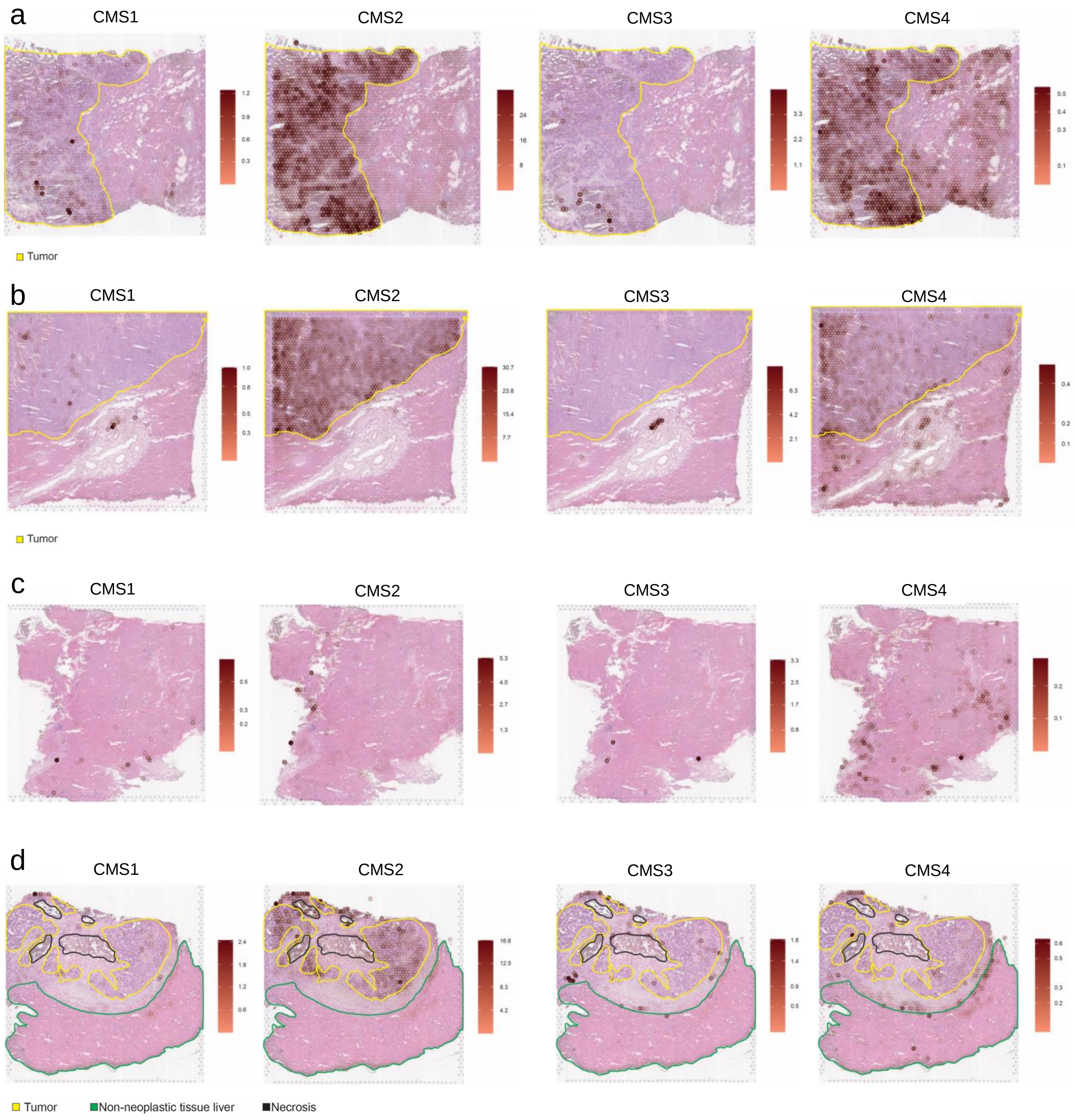

Supplementary Figure S21: *Spatial mapping of the predicted number of tumor cells of the different Consensus Molecular Subtypes (CMS) of CRC according to the deconvolution results, overlaying with the pathologist's tissue annotations for the samples: a) ST-liver1_Unt, b) ST-liver2_Unt, c) ST-liver3_Tre, and d) ST-liver4_Tre.*

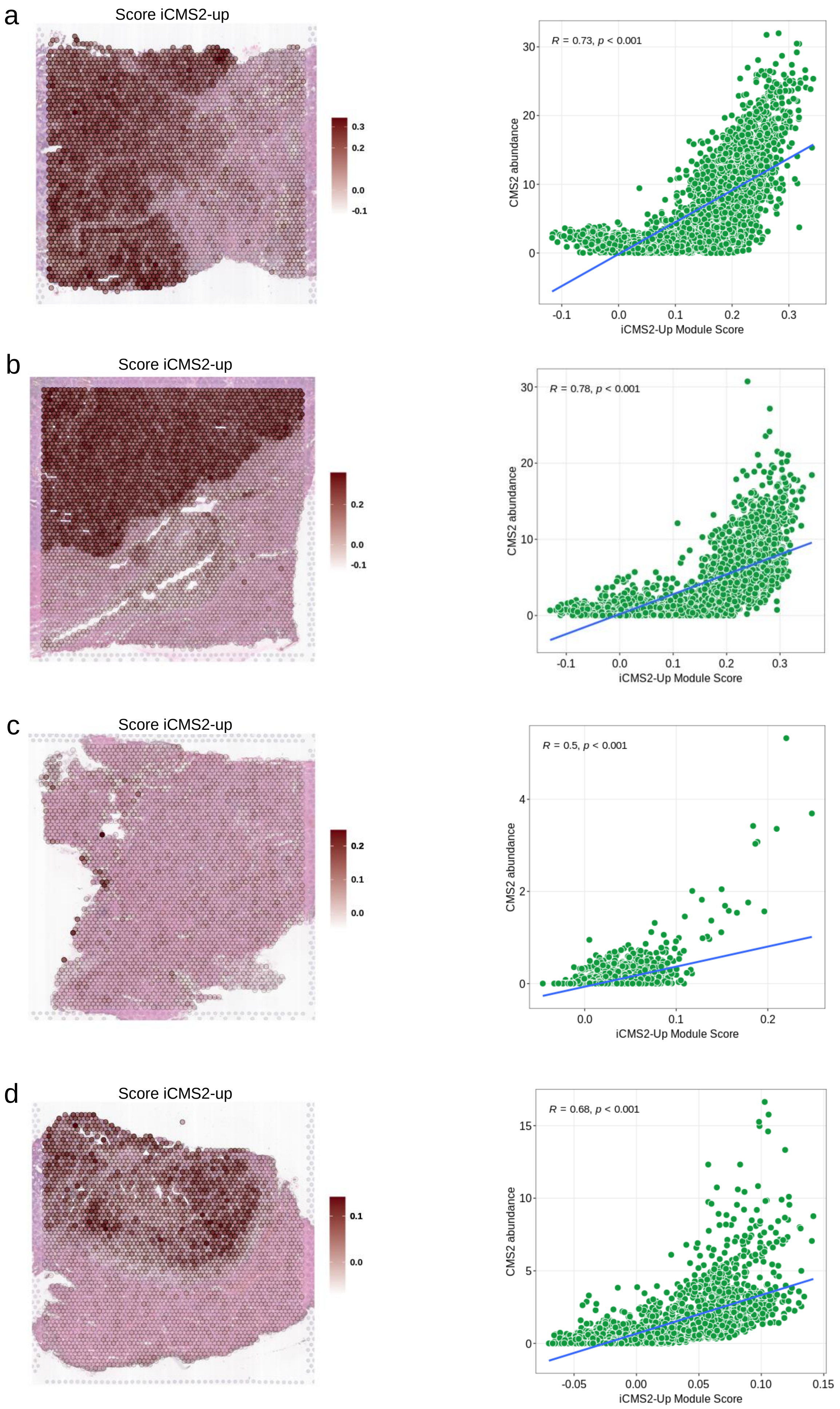
a
Score iCMS2-up
0.3
0.2
0.0
-0.1
R = 0.73, p < 0.001
CMS2 abundance
iCMS2-Up Module Score
b
Score iCMS2-up
0.2
0.0
-0.1
R = 0.78, p < 0.001
CMS2 abundance
iCMS2-Up Module Score
c
Score iCMS2-up
0.2
0.1
0.0
R = 0.5, p < 0.001
CMS2 abundance
iCMS2-Up Module Score
d
Score iCMS2-up
0.1
0.0
R = 0.68, p < 0.001
CMS2 abundance
iCMS2-Up Module Score

Supplementary Figure S22: *Spatial mapping of the iCMS2-upregulated module score and the Pearson's correlation between this score and the deconvolution-predicted CMS2 abundance for the samples: a) ST-liver1_Unt, b) ST-liver2_Unt, c) ST-liver3_Tre, and d) ST-liver4_Tre.*

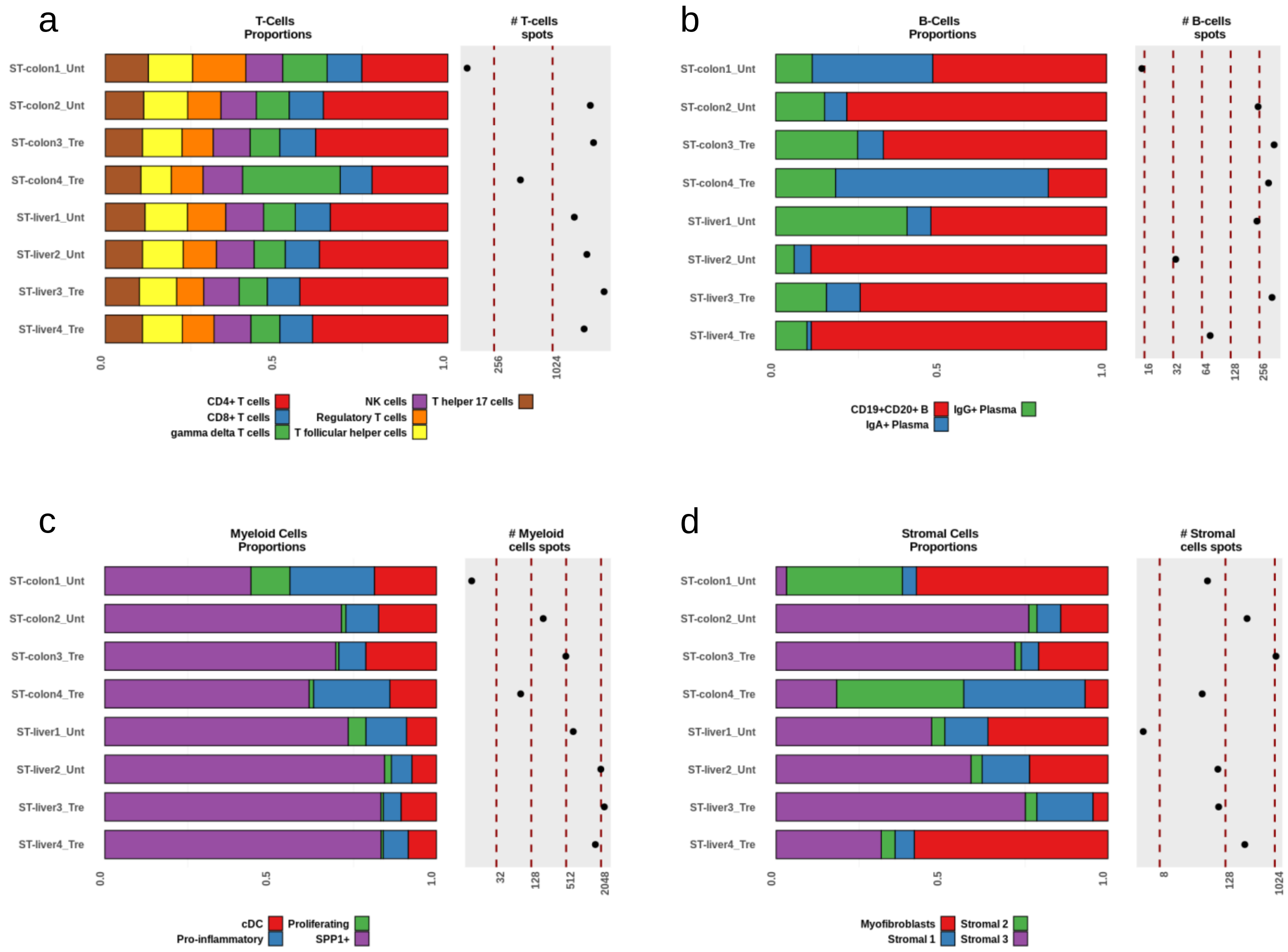

Supplementary Figure S23: *Per sample proportions of the a)T-cells subtypes, b) B-cells subtypes, c) myeloid cells subtypes, and d) stromal cells subtypes, as estimated by the results of the deconvolution approach. The number of spots containing an abundance of at least 20% of the respective cell types are also displayed.*

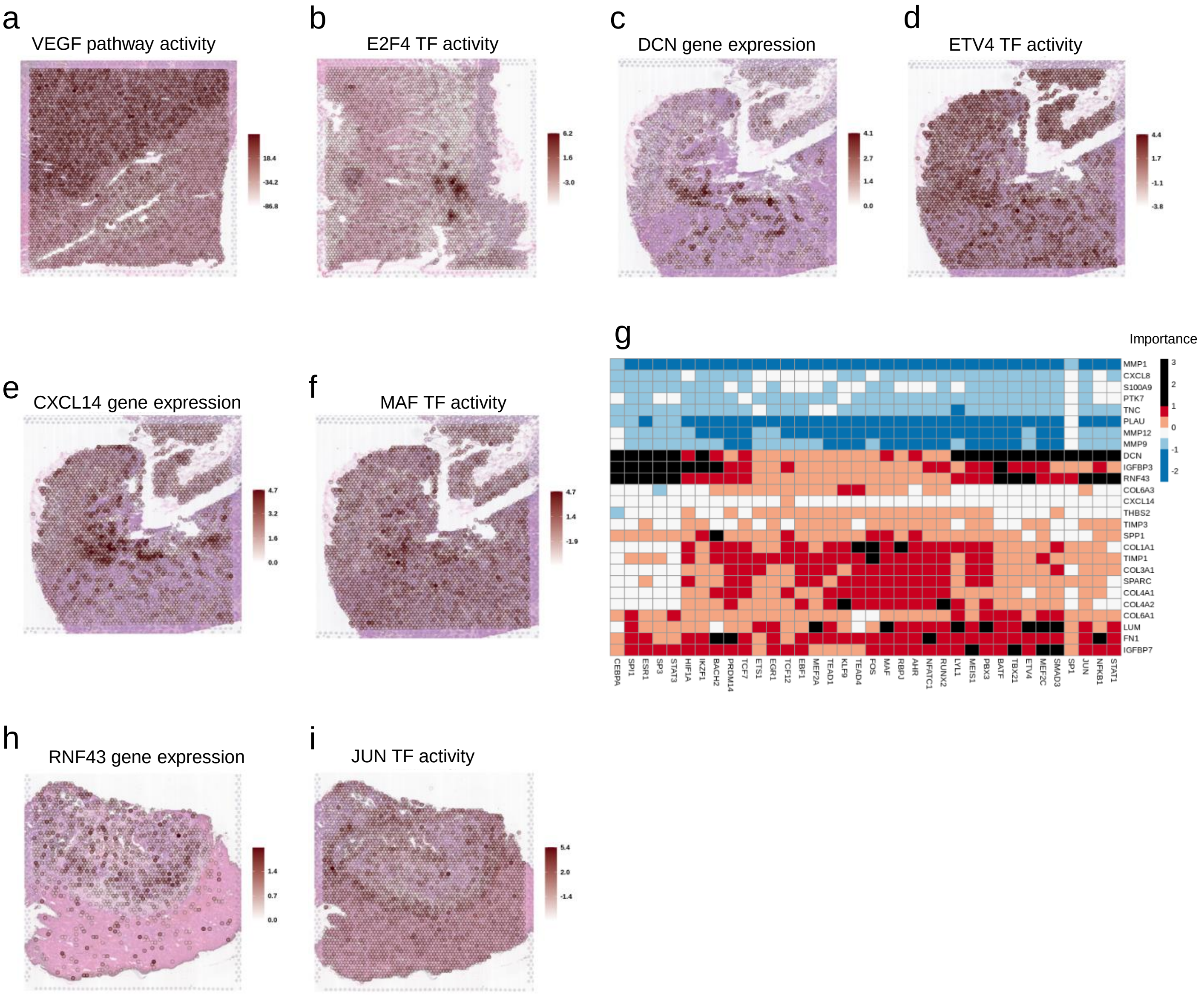

Supplementary Figure S24: *Spatial mapping of: a) the VEGF pathway activity in the ST-liver2_Unt sample, and b) the E2F4 TF activity in the ST-colon2_Unt sample. Spatial mapping of: c) DCN gene expression, d) ETV4 TF activity, e) CXCL14 gene expression, and f) MAS TF activity in the ST-colon1_Unt sample; g) Misty results showing the potential importance of ligands (rows) expression on TF (columns) activity when considering the samples with hepatic metastasis of CRC tumors. The ligand-TFs relationships with an importance score over 1 are represented as black slots and were considered as relevant. Spatial mapping of: h) RNF43 gene expression and i) JUN TF activity in the ST-liver4_Tre sample.*

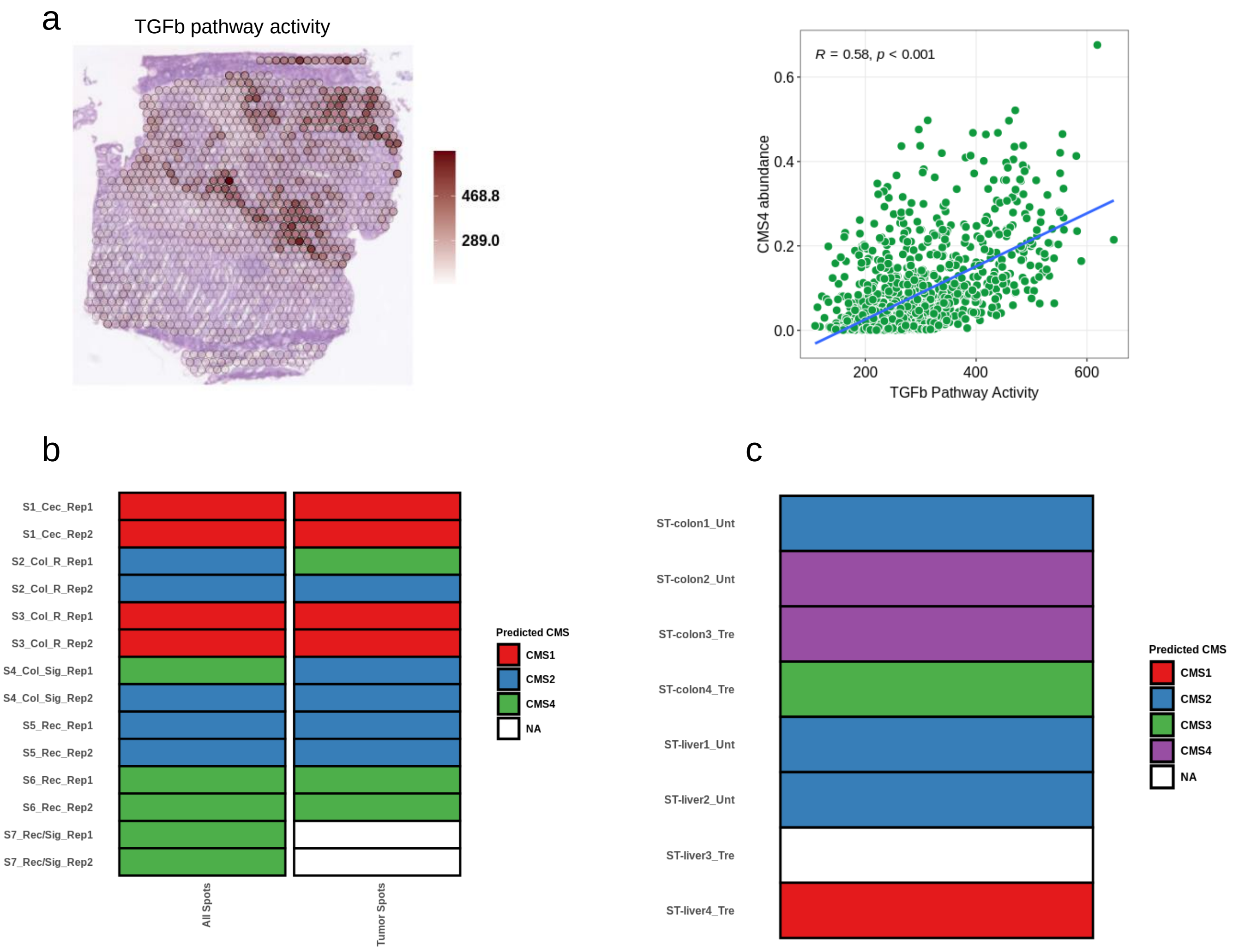

Supplementary Figure S25: *a) Spatial mapping of the estimated TFGb pathway activity for the sample S2_Col_R_Rep1. Pearson's correlation between this activity and the deconvolution-predicted CMS4 abundance is also shown; b) Predicted CMS classification resulting from applying CMScaller on pseudo-bulk RNA-seq generated by either pooling together all the spots or only the tumor annotated spots for each of our CRC samples; c) Predicted CMS classification resulting from applying CMScaller on pseudo-bulk RNA-seq generated by pooling together all the spots from the external ST CRC dataset.*

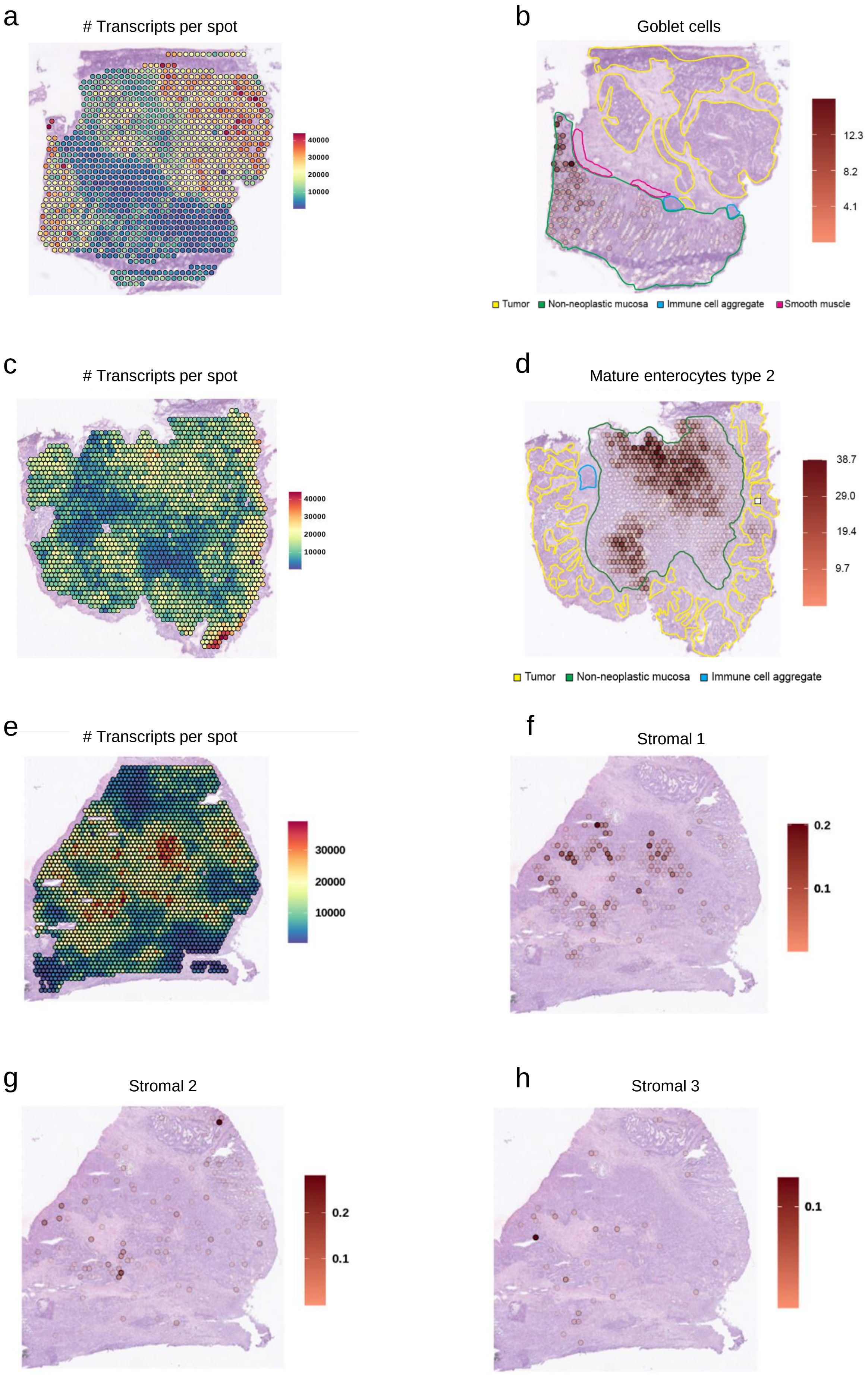

a
Transcripts per spot
40000
30000
20000
10000
b
Goblet cells
12.3
8.2
4.1
Tumor
Non-neoplastic mucosa
Immune cell aggregate
Smooth muscle
c
Transcripts per spot
40000
30000
20000
10000
d
Mature enterocytes type 2
38.7
29.0
19.4
9.7
Tumor
Non-neoplastic mucosa
Immune cell aggregate
e
Transcripts per spot
30000
20000
10000
f
Stromal 1
0.2
0.1
g
Stromal 2
0.2
0.1
h
Stromal 3
0.1

Supplementary Figure S26: *Spatial mapping of a) the number of transcript per spot, and b) the predicted abundance of goblet cells for the S2_Col_R_Rep1 sample. Spatial mapping of: c) the number of transcript per spot, and d) the predicted abundance of mature enterocytes type 2 cells for the S5_Rec_Rep1 sample. It is to note that the regions of the non-neoplastic mucosa showing a lower number of transcripts per spot were accordingly predicted to have a reduced amount of goblet cells and mature enterocytes type 2. Pathologist's tissue annotations are also displayed for clarification. Spatial mapping of the number of transcript per spot (e) and the predicted abundance of the different stromal main populations, excluding myofibroblasts, (f-h) for the S3_Col_Rep1 sample. The regions with lower number of transcripts match with stromal morphological features, however non stromal abundance was mapped into those areas.*

2. Supplementary Tables

Cell type	*Cell subtype*	*Comments*	*Number of Cells*	*Genes used for deconvolution*
B cells	*CD19+CD20+ B*	*B lymphocytes*	*3733*	*38*
B cells	*IgA+ Plasma*	*B lymphocytes*	*7305*	*7*
B cells	*IgG+ Plasma*	*B lymphocytes*	*6404*	*15*
Epithelial cells	*CMS1*	*Tumor cells*	*1201*	*412*
Epithelial cells	*CMS2*	*Tumor cells*	*10771*	*620*
Epithelial cells	*CMS3*	*Tumor cells*	*5486*	*228*
Epithelial cells	*CMS4*	*Tumor cells*	*11*	*22*
Epithelial cells	*Goblet cells*	*Normal epithelial cells*	*305*	*176*
Epithelial cells	*Intermediate*	*Normal epithelial cells*	*139*	*131*
Epithelial cells	*Mature Enterocytes type 1*	*Normal epithelial cells*	*3546*	*182*
Epithelial cells	*Mature Enterocytes type 2*	*Normal epithelial cells*	*1755*	*172*
Epithelial cells	*Stem-like/TA*	*Normal epithelial cells*	*181*	*14*
Mast cells	*Mast cells*	*Normal epithelial cells*	*108*	*186*
Myeloids	*cDC*	*Conventional dendritic cells*	*187*	*43*
Myeloids	*Pro-inflammatory*	*Pro-inflammatory Macrophages*	*213*	*77*
Myeloids	*Proliferating*	*Proliferating Macrophages*	*131*	*73*
Myeloids	*SPP1+*	*SPP1+ Macrophages*	*1155*	*305*
Stromal cells	*Enteric glial cells*		*2399*	*55*
Stromal cells	*Lymphatic ECs*	*Lymphatic Endothelial Cells*	*171*	*239*

Stromal cells	*Myofibroblasts*		*44*	*521*
Stromal cells	*Pericytes*		*3097*	*32*
Stromal cells	*Proliferative ECs*	*Proliferative Endothelial Cells*	*3252*	*168*
Stromal cells	*Smooth muscle cells*		*214*	*151*
Stromal cells	*Stalk-like ECs*	*Stalk-like Endothelial Cells*	*438*	*99*
Stromal cells	*Stromal 1*		*406*	*618*
Stromal cells	*Stromal 2*		*1045*	*94*
Stromal cells	*Stromal 3*		*394*	*171*
Stromal cells	*Tip-like ECs*	*Tip-like Endothelial Cells*	*868*	*381*
T cells	*CD4+ T cells*	*T lymphocytes*	*620*	*25*
T cells	*CD8+ T cells*	*T lymphocytes*	*1978*	*28*
T cells	*gamma delta T cells*	*T lymphocytes*	*917*	*265*
T cells	*NK cells*	*Natural Killer T lymphocytes*	*445*	*248*
T cells	*Regulatory T cells*	*T lymphocytes*	*2181*	*16*
T cells	*T follicular helper cells*	*T lymphocytes*	*482*	*200*
T cells	*T helper 17 cells*	*T lymphocytes*	*996*	*37*

Supplementary Table S1: *Cell types and subtypes as annotated in the scRNA-seq used as reference for the deconvolution process. We show the number of cells per type in that dataset and the number of genes used for deconvolution. We additionally include a column with comments for further clarification. Of Note: there are also groups of Myeloid, B cells and T cells annotated as unknown at the cell subtype level.*

ID	Gene Ratio	BgRatio	pvalue	p.adjust	qvalue	geneID
Patient: *S2_ Col_ R*						
WNT_BETA_CATENIN_SIGNALING	*6/168*	*40/4153*	*0.0048993*	*0.2012755*	*0.1906821*	*NKD1/NOTCH1/LEF1/CTNNB1/PTCH1/AXIN2*
HEDGEHOG_SIGNALING	*5/168*	*34/4153*	*0.0109591*	*0.2012755*	*0.1906821*	*AMOT/ADGRG1/PTCH1/CDK6/VEGFA*
MYC_TARGETS_V1	*15/168*	*196/4153*	*0.0120765*	*0.2012755*	*0.1906821*	*SNRPD1/HSP90AB1/CCT4/ACP1/CCT7/PCBP1/YWHAQ/RAD23B/YWHAE/XPO1/GSPT1/SET/SSBP1/EIF4H/RNPS1*
Patient: *S4_ Col_ Sig*						
WNT_BETA_CATENIN_SIGNALING	*6/81*	*40/4153*	*0.0001033*	*0.0044404*	*0.0040219*	*NKD1/AXIN2/KAT2A/CCND2/AXIN1/HDAC5*
MTORC1_SIGNALING	*10/81*	*198/4153*	*0.0046955*	*0.1009540*	*0.0914394*	*PPIA/CYB5B/PHGDH/NUPR1/SQSTM1/CCT6A/EIF2S2/MCM4/HSPD1/SQLE*
MYC_TARGETS_V1	*9/81*	*196/4153*	*0.0132326*	*0.1896669*	*0.1717913*	*PPIA/CBX3/EIF2S2/POLD2/MCM4/HNRNPA2B1/GNL3/HSPD1/SRSF2*
Patient: *S5_ Rec*						
MYC_TARGETS_V1	*18/90*	*196/4153*	*0.0000001*	*0.0000050*	*0.0000044*	*MYC/HSPE1/PGK1/ODC1/PHB/KPNA2/PSMA7/SNRPG/RAN/HSPD1/NME1/NOP56/HPRT1/TFDP1/PRDX4/STARD7/DEK/PRPS2*
E2F_TARGETS	*14/90*	*198/4153*	*0.0000722*	*0.0015530*	*0.0013687*	*MYC/TFRC/DCTPP1/KPNA2/CDC25B/RAN/NME1/AURKA/NOP56/SNRPB/CSE1L/PRDX4/DEK/HMGB3*
MYC_TARGETS_V2	*7/90*	*58/4153*	*0.0002173*	*0.0031145*	*0.0027447*	*MYC/HSPE1/PHB/DCTPP1/HSPD1/NOP56/SORD*
G2M_CHECKPOINT	*11/90*	*194/4153*	*0.0028328*	*0.0285653*	*0.0251738*	*MYC/HSPA8/SLC12A2/ODC1/KPNA2/CDC25B/DKC1/AURKA/UBE2C/TFDP1/HMGB3*
MTORC1_SIGNALING	*11/90*	*198/4153*	*0.0033215*	*0.0285653*	*0.0251738*	*NAMPT/HSPE1/PGK1/TFRC/UFM1/HSPD1/ALDOA/AURKA/HPRT1/PSME3/SORD*
TNFA_SIGNALING_VIA_NFKB	*9/90*	*200/4153*	*0.0279594*	*0.2003756*	*0.1765856*	*CXCL1/CXCL3/NAMPT/MYC/RHOB/PHLDA2/TGIF1/CCL20/ETS2*
Patient: *S6_ Rec*						
HYPOXIA	*5/24*	*195/4153*	*0.0044378*	*0.1302267*	*0.1282368*	*TGFBI/ERRFI1/PPP1R15A/TPI1/GAPDH*
CHOLESTEROL_HOMEOSTASIS	*3/24*	*74/4153*	*0.0084017*	*0.1302267*	*0.1282368*	*ERRFI1/CXCL16/TNFRSF12A*

Supplementary Table S2: *Significant enrichment results (q<0.2) on the differentially expressed genes between the CMS2 tumors of the different patients.* ***GeneRatio*** *indicates the number of DEG belonging to the enriched category versus the total number of DEG for that patient.* ***BgRatio*** *describes the number of genes in the background belonging to the enriched category versus the total number of background genes.* ***geneID*** *details the DEG matching the enriched category.*

ID	Gene Ratio	BgRatio	pvalue	p.adjust	qvalue	geneID
Region: *Outer Tumor*						
EPITHELIAL MESENCHYMAL TRANSITION	*44/97*	*188/3726*	*0*	*0*	*0*	*COL6A2/SPARC/FBLN1/FN1/COL3A1/LUM/MGP/COL1A1/COL1A2/THY1/BGN/TAGLN/TIMP3/POSTN/SFRP4/VCAN/ACTA2/COL5A2/VIM/HTRA1/CCN1/DCN/FSTL1/GREM1/TPM2/COL4A1/CALD1/IGFBP4/FLNA/CTHRC1/MMP2/DPYSL3/COL4A2/COL6A3/TIMP1/MYL9/ELN/LGALS1/COL5A1/THBS2/IGFBP3/FBLN2/ITGB1/NID2*
COAGULATION	*17/97*	*102/3726*	*0*	*0*	*0*	*C3/SPARC/FN1/MMP11/APOC1/C1R/MMP9/TIMP3/CTSK/A2M/C1S/HTRA1/MMP2/TIMP1/SERPING1/PECAM1/CTSB*
MYOGENESIS	*15/97*	*148/3726*	*0.0000044*	*0.0000561*	*0.0000513*	*COL6A2/SPARC/COL3A1/COL1A1/TAGLN/IGFBP7/TPM2/AEBP1/COL15A1/COL4A2/COL6A3/LSP1/IGFBP3/WWTR1/ITGB1*
ANGIOGENESIS	*7/97*	*30/3726*	*0.0000081*	*0.0000773*	*0.0000706*	*COL3A1/LUM/POSTN/VCAN/COL5A2/FSTL1/TIMP1*
COMPLEMENT	*13/97*	*167/3726*	*0.0003201*	*0.0024328*	*0.0022239*	*C3/FN1/APOC1/C1R/TIMP2/C1S/COL4A2/TIMP1/SERPING1/PRCP/ANXA5/CTSB/CTSD*
Region: *Intermediary Tumor*						
No significant results						
Region: *Inner Tumor*						
HYPOXIA	*19/102*	*177/3726*	*0.0000002*	*0.0000083*	*0.0000067*	*ATF3/P4HA1/VEGFA/LDHA/BHLHE40/SLC2A1/P4HA2/DDIT4/ENO1/NDRG1/ALDOA/JUN/PGK1/TPI1/MIF/ALDOC/IER3/GAPDH/SULT2B1*
CHOLESTEROL HOMEOSTASIS	*10/102*	*70/3726*	*0.0000153*	*0.0002581*	*0.0002081*	*SCD/ATF3/LDLR/FASN/ALDOC/ACTG1/ACSS2/CYP51A1/S100A11/LGALS3*
MTORC1 SIGNALING	*17/102*	*196/3726*	*0.0000165*	*0.0002581*	*0.0002081*	*INSIG1/SCD/P4HA1/LDHA/BHLHE40/SLC2A1/LDLR/DDIT4/ENO1/BTG2/ALDOA/UCHL5/PGK1/TPI1/GAPDH/CYP51A1/SERP1*
GLYCOLYSIS	*16/102*	*183/3726*	*0.0000273*	*0.0003207*	*0.0002586*	*P4HA1/VEGFA/LDHA/P4HA2/DDIT4/ENO1/TFF3/PKM/ELF3/SDHC/ALDOA/PGK1/TPI1/MIF/IER3/CLDN3*
TNFA SIGNALING VIA NFKB	*16/102*	*191/3726*	*0.0000465*	*0.0004370*	*0.0003523*	*CCL20/ATF3/AREG/VEGFA/EGR3/BHLHE40/LDLR/BTG2/EGR1/JUN/SNN/ZC3H12A/IER3/TNFAIP2/BIRC3/IFIT2*
ESTROGEN RESPONSE EARLY	*13/102*	*183/3726*	*0.0012558*	*0.0098369*	*0.0079312*	*AREG/EGR3/BHLHE40/SLC2A1/TFF3/FASN/ELF3/TFF1/KCNK5/SULT2B1/SLC22A5/MUC1/PEX11A*

Supplementary Table S3: *Significant enrichment results (p-value <0.01) on the differentially expressed genes between the different anatomical regions of the CMS2 tumor in the S2_Col_R_Rep1 sample.* ***GeneRatio*** *indicates the number of DEG belonging to the enriched category versus the total number of DEG for that patient.* ***BgRatio*** *describes the number of genes in the background belonging to the enriched category versus the total number of background genes.* ***geneID*** *details the DEG matching the enriched category.*

ID	*Gene Ratio*	*BgRatio*	*pvalue*	*p.adjust*	*qvalue*	*geneID*
Region: *Sub-Cluster 1*						
TNFA SIGNALING VIA NFKB	*49/410*	*200/4346*	*0*	*0*	*0*	*TRIB1/CXCL1/AREG/PLAUR/NFKBIE/RHOB/KYNU/CXCL3/KLF4/GADD45B/MXD1/MYC/JAG1/FOS/BCL3/LDLR/JUNB/JUN/ATF3/LIF/MCL1/ZFP36/BHLHE40/IRF1/CEBPD/CCNL1/ZC3H12A/KLF10/IER3/CXCL2/IER2/B4GALT1/RNF19B/CDKN1A/ID2/DUSP2/PHLDA2/KLF6/NFE2L2/EDN1/LAMB3/TAP1/DUSP1/NFKBIA/BTG1/TGIF1/VEGFA/BTG2/NR4A1*
CHOLESTEROL HOMEOSTASIS	*21/410*	*74/4346*	*0.0000024*	*0.0000585*	*0.0000427*	*PLAUR/FDFT1/PLSCR1/ANTXR2/SQLE/ACTG1/CLU/FABP5/S100A11/FDPS/JAG1/HMGCS1/LDLR/MAL2/ATF3/HMGCR/LGALS3/ERRFI1/NSDHL/CYP51A1/TMEM97*
P53 PATHWAY	*37/410*	*196/4346*	*0.0000224*	*0.0003660*	*0.0002673*	*GPX2/TOB1/TM4SF1/ITGB4/HINT1/IRAK1/PROCR/RAP2B/EPHA2/KLF4/MXD1/SDC1/NDRG1/TXNIP/FOS/PRMT2/JUN/EPS8L2/ATF3/LIF/STEAP3/IER3/RNF19B/CDKN1A/SFN/SLC7A11/PLXNB2/AK1/CTSD/TAP1/VAMP8/SEC61A1/BTG1/CEBPA/DDIT4/BTG2/ZFP36L1*
UV RESPONSE UP	*29/410*	*156/4346*	*0.0002339*	*0.0028652*	*0.0020927*	*GGH/DGAT1/ATP6V1C1/GRINA/OLFM1/EPCAM/RHOB/STK25/IGFBP2/POLR2H/FOS/DNAJA1/DNAJB1/JUNB/ATF3/IRF1/FKBP4/FURIN/CXCL2/HLA-F/SPR/WIZ/TAP1/TUBA4A/GLS/NFKBIA/BTG1/BTG2/NR4A1*
GLYCOLYSIS	*34/410*	*198/4346*	*0.0003366*	*0.0032983*	*0.0024090*	*PPP2CB/IDH1/HSPA5/AK3/SDC1/QSOX1/EGFR/MET/GPC4/ELF3/CHPF/HDLBP/FKBP4/MDH1/B3GNT3/IER3/CHPF2/B4GALT1/IL13RA1/ENO1/HK2/RBCK1/DSC2/NSDHL/SLC16A3/GCLC/TGFBI/AURKA/SOX9/STMN1/CLDN3/PYGB/VEGFA/DDIT4*
TGF BETA SIGNALING	*13/410*	*54/4346*	*0.0011700*	*0.0090233*	*0.0065906*	*ID1/SLC20A1/SPTBN1/FNTA/JUNB/FKBP1A/KLF10/FURIN/ID2/THBS1/ID3/SKIL/TGIF1*
INTERFERON ALPHA RESPONSE	*19/410*	*96/4346*	*0.0012890*	*0.0090233*	*0.0065906*	*LY6E/HLA-C/LGALS3BP/PLSCR1/IFI27/PROCR/IFI35/GBP4/TXNIP/PARP14/PSME2/OGFR/IRF1/LAP3/PARP9/ADAR/CD47/TAP1/IL4R*
HYPOXIA	*32/410*	*200/4346*	*0.0017387*	*0.0106494*	*0.0077783*	*ANXA2/GAA/PLAUR/JMJD6/HSPA5/NDRG1/GRHPR/FOS/EGFR/GPC4/JUN/ATF3/ZFP36/BHLHE40/HDLBP/IER3/ENO1/CDKN1A/IDS/HK2/SLC2A1/ERRFI1/PFKL/KLF6/TGFBI/DUSP1/ILVBL/BTG1/PLAC8/VEGFA/DDIT4/SELENBP1*
MTORC1 SIGNALING	*31/410*	*198/4346*	*0.0028992*	*0.0157847*	*0.0115291*	*IFRD1/ACSL3/SQLE/IDH1/HSPE1/HSPA5/HSPD1/SORD/HMGCS1/LDLR/GSR/GLA/BHLHE40/SLC1A5/CCT6A/PSME3/HMGCR/ENO1/CDKN1A/HK2/SLC2A1/SLC7A11/PFKL/CYP51A1/ACLY/TMEM97/GCLC/TUBA4A/AURKA/DDIT4/BTG2*
APOPTOSIS	*26/410*	*159/4346*	*0.0034054*	*0.0166862*	*0.0121876*	*SLC20A1/TSPO/PEA15/RHOB/HSPB1*

						/CLU/GADD45B/BCAP31/TXNIP/DNAJA1/GSR/JUN/ATF3/MCL1/IRF1/TIMP1/IER3/CDKN1A/LGALS3/PMAIP1/SPTAN1/ERBB2/TAP1/CDC25B/IFNGR1/BTG2
IL2 STAT5 SIGNALING	*30/410*	*199/4346*	*0.0059433*	*0.0264748*	*0.0193371*	*PLEC/MYO1C/ITGAV/ITGA6/CAPG/PLSCR1/RHOB/GADD45B/MXD1/GBP4/MYC/NDRG1/NFKBIZ/CDCP1/CTSZ/AHCY/ARL4A/PRNP/LIF/BHLHE40/SLC1A5/HUWE1/FURIN/ST3GAL4/HK2/TNFRSF21/ANXA4/KLF6/IL4R/IFNGR1*
**Region: *Sub-Cluster 2***						
MYC TARGETS V1	*17/88*	*196/4346*	*0.0000002*	*0.0000101*	*0.0000093*	*HNRNPA1/PA2G4/TFDP1/RRM1/HPRT1/DEK/NDUFAB1/CLNS1A/NAP1L1/SLC25A3/LDHA/SYNCRIP/NPM1/RNPS1/SNRPD2/PCNA/COX5A*
OXIDATIVE PHOSPHORYLATION	*16/88*	*200/4346*	*0.0000017*	*0.0000359*	*0.0000333*	*COX6A1/ATP5MG/CS/NDUFAB1/COX4I1/SLC25A5/ATP5F1B/SLC25A3/LDHA/NDUFC2/MRPL11/UQCRC2/COX5A/ATP5F1C/LDHB/FDX1*
E2F TARGETS	*12/88*	*198/4346*	*0.0005546*	*0.0077642*	*0.0071998*	*PA2G4/HMGB2/DEK/NAP1L1/SMC1A/HMGA1/DCTPP1/SYNCRIP/MCM3/TMPO/PCNA/TP53*
**Region: *Sub-Cluster 3***						
COAGULATION	*12/51*	*138/4346*	*0*	*0.0000013*	*0.0000011*	*CFB/MMP3/C2/FN1/MMP1/C3/C1R/SERPINA1/PLAU/CTSK/MMP2/A2M*
KRAS SIGNALING UP	*8/51*	*199/4346*	*0.0019551*	*0.0235473*	*0.0198293*	*CFB/TMEM176A/PIGR/TMEM176B/CCL20/MAP7/IGFBP3/PLAU*
COMPLEMENT	*8/51*	*200/4346*	*0.0020183*	*0.0235473*	*0.0198293*	*CFB/C2/FN1/C3/MMP12/C1R/S100A9/SERPINA1*
INTERFERON ALPHA RESPONSE	*5/51*	*96/4346*	*0.0049834*	*0.0436049*	*0.0367199*	*IFITM3/IFITM2/NCOA7/IFI30/CD74*
INTERFERON GAMMA RESPONSE	*7/51*	*198/4346*	*0.0077083*	*0.0474365*	*0.0399466*	*CFB/SOD2/IFITM3/IFITM2/C1R/IFI30/CD74*
EPITHELIAL MESENCHYMAL TRANSITION	*7/51*	*200/4346*	*0.0081320*	*0.0474365*	*0.0399466*	*MMP3/FN1/MMP1/TNC/IGFBP3/CXCL8/MMP2*

Supplementary Table S4: *Significant enrichment results (p-value <0.01) on the differentially expressed genes between the different sub-clustered regions of the CMS2 tumor in the S5_Rec_Rep1 sample.* ***GeneRatio*** *indicates the number of DEG belonging to the enriched category versus the total number of DEG for that patient.* ***BgRatio*** *describes the number of genes in the background belonging to the enriched category versus the total number of background genes.* ***geneID*** *details the DEG matching the enriched category.*

Pathologist's spot categorization	
**Category**	**Categorization criteria**
non neoplastic epithelium	>90% non neoplastic epithelial cells
submucosa	>90% submucosa
epithelium&submucosa	epithelium and submucosa: mixed spots 10-90% epithelium, 10-90% submucosa
tumor	>90% neoplastic epithelial cells
stroma_fibroblastic_IC low	fibroblast rich, collagen poor stroma, IC <10%, low nuclear density
stroma_fibroblastic_IC med	20- 50% IC, moderate nuclear density, HE: myofibroblast-like morphology
stroma_fibroblastic_IC high	51- 80% IC, high nuclear density
stroma_desmoplastic_IC low	fibroblast poor, collagen rich stroma, IC <10%, low nuclear density
stroma_desmoplastic_IC med to high	IC >10%, low to moderate nuclear density
tumor&stroma_IC med to high	mixed spots, <90% tumor, <90% stroma, IC in stroma 20-90%
tumor&stroma_IC low	mixed spots, <90% tumor, <90% stroma, IC in stroma <10%
IC aggregate_submucosa	follicular immune cell aggregates>90% IC
IC aggregate_stroma or muscularis	follicular immune cell aggregates>90% IC
muscularis_IC med to high	smooth muscle cells, IC content 20-90%
exclude	spots of poor tissue quality or not classifiable

Supplementary Table S5: *Pathologist's spot categorization and categorization criteria. Each spot was manually assigned to a category in the HE stained OCT tissue section the Loupe Browser. IC=immune cells*

Semiquantitative pathologist's grading and assessment of the deconvolution results						
Patient	***CMS1***	***CMS2***	***CMS3***	***CMS4***	***Selected immune features***	***Selected stroma features***
S1_ Cec A551763	++	++	*(+)*	-	+ *CD8 T cells*<br>+ *CD19/CD20 B-Cells*	++ *Myofibroblasts*
S2_ Col_ R A595688	-	+++	*	*(+)*	+ *SPP1-positive MA*<br>+ *Pro-inflammatory MA*<br>+ *CD4 T-cells*<br>+ *Treg*<br>+ *IgG Plasma*	++ *Myofibroblasts (perilobular)*<br>+ *Stroma 1 (non-neoplastic EP)*<br>++ *Stroma 2 (intralobular)*<br>+ *Stroma 3 (perilobular)*
S3_ Col_ R A416371	+++	++	*(+)*	*(+)*	+ *SPP1-positive MA*<br>+++ *CD8 T cells*<br>+++ *CD19/CD20 B-cells*<br>+ *NK cells*	*(+) Myofibroblasts*<br>*80% of stroma without signature*
S4_ Col_ Sig A120838	-	+++	-	*(+)*	+ *SPP+ MA*	++ *Myofibroblasts*<br>*(+) Stroma 2 (intralobular)*<br>+ *Stroma 3*<br>*50% of the stroma without signature*
S5_ Rec A121573	*(+)*	+++	*	*(+)*	*Immune poor*	*(+) Myofibroblasts*<br>+ *Stroma 1 (non-neoplastic EP)*<br>*(+) Stroma 2 (non-neoplastic EP and neoplastic)*
S6_ Rec A938797	*(+)*	+++	*	*(+)*	+ *SPP1-positive MA*<br>+ *Mast cells*<br>+ *CD4, CD8, T-reg, T-foll helper*<br>+ *CD19/20 B-cells*<br>+ *IgG plasma cells*	+++ *Myofibroblasts*<br>+ *Stroma 1 (non-neoplastic EP and neoplastic)*<br>++ *Stroma 2*<br>++ *Stroma 3*
S7_ Rec/ Sig A798015	**	**	*	**	+ *Mast cells*<br>+ *IgG plasma cells*	++ *Myofibroblasts (non-neoplastic CT)*<br>+ *Stroma 2 (non-neoplastic EP)*<br>++ *Stroma 3 (non-neoplastic CT)*

Supplementary Table S6: Semiquantitative grading of our set of CRC samples according to the cell type abundance of the different CMS: *Selected immune and stromal features are also specified for each sample. MA= macrophages; R=right, EP=epithelium, CT=connective tissue*

(-) Highest percentage of cells in tumor/stroma tissue-associated spots <0.1;

(+) Percentage of cells per spot 0.2-1 in 10-30% of tumor/stroma tissue-associated spots;

+ Percentage of cells per spot 0.2-1 in 31-50% of tumor/stroma tissue-associated spots;

++ Percentage of cells per spot 1-5 in 50-100% of tumor tissue-associated spots;

+++ Percentage of cells per spot <5 in 50-100% of tumor tissue-associated spots;

**CMS3 signature (percentage of cells per spot 1-5) in the non-neoplastic mucosa;*

***Low signature (percentage of cells per spot 0.2-1) in the non-neoplastic mucosa/connective tissue.*

3. Supplementary Note 1

Comparison of deconvolution results using the scRNA-seq data from the Korean or the Belgian cohort

In the main manuscript, we presented the results of the Cell2Location[1] deconvolution approach when using as reference the cell type annotations from a scRNAseq dataset derived from a Korean cohort of 23 patients. This Korean dataset proceeds from the study by Lee et al.[2], in which an additional scRNAseq dataset from a Belgian cohort of 6 patients was independently analyzed and annotated. Both datasets present some relevant differences, such as an enrichment of fibroblasts and myeloid cells in the Belgian dataset. Besides, a few cell types were only identified in one of the datasets. This is the case of the tuft cells, which were only detected in the Belgian dataset. Consequently and in line with the original publication, we performed the deconvolution in parallel with each dataset. In this supplementary note, we compared the results yielded by both procedures.

We first compared the deconvolution results across major cell types (Fig. 1). Overall the cell type proportions present similar trends across our set of ST samples when using the references derived from both datasets. However, there are some noticeable and relevant differences. Excluding the sample containing only non neoplastic tissue, there is a predominant increase in the proportions of tumor cells mapped into our samples when using the Belgian cohort. For some samples, the intensification of tumorigenic signals seems to be mainly at the expense of the normal epithelial cells. This is for instance the case in the S5_Rec sample. In other samples, such as in S3_Col_R, there is a marked decrease in the predicted abundance of B cells.

In order to get a better understanding of these discrepancies, we explored the proportions at the cell subtype level. We first focused on the different CMS tumor cell populations (Fig. 2). Overall, the results are highly comparable using either of the two reference datasets. The samples referred to as CMS2 tumors in the main manuscript also display a clear predominance of this tumor cell type when using the Belgian reference. Concretely, the percentages of CMS2 tumor

cells as compared to the total number of tumor cells in these patients were: S2_Col_R (89%), S4_Col_Sig (92%), S5_Rec (86%) and S6_Rec (88%). In addition, the S1_Cec (30% and 68%) and S3_Col_R (45% and 51%) patients maintain a mixed proportion of CMS1 and CMS2 tumor cells. Interestingly, for the Belgian reference, the samples labeled as CMS2 tumors present a more prominent CMS1 signal, whereas the mixed CMS1-CMS2 tumors display a slight decrease in the proportion of CMS1 cell abundances. To get further insights on these differences, we explored the tumor cell abundance spatial mappings per spot in our samples (Fig. 3-9). As expected from the global tumor proportions, CMS1 cells displayed the most prominent differences between both references. In the samples labeled as CMS2 tumors, the number of predicted CMS1 cells was very limited and did not display a clear spatial distribution when using the Korean reference. However, using the Belgian reference conducted to stronger CMS1 signals overlapping to a considerable degree with the anatomical regions where the presence of CMS2 cells was predicted. In the CMS1-CMS2 mixed tumors, the predicted spatial location of CMS1 cells matched between references. Across our set of samples, the predicted anatomical location of CMS2, CMS3 and CMS4 cells matched to a large extent for both used references. There were nevertheless differences in their estimated abundance, mainly for CMS2, which presented an increased number of cells predicted per spot when using the Belgian reference. This growth in the number of predicted CMS2 tumor cells can account for the global larger proportions of tumor cells detected per sample (see Fig.1).

We subsequently compared the deconvolution results provided by the different references for immune cells. The estimated proportions per sample were highly comparable for T- (Fig. 10) and B-cells (Fig. 11). Despite some discrepancies in the abundance of $CD8^+$ T cells in some samples, such as in *S3_Col*, their predicted anatomical locations overlapped using either reference dataset (Fig. 12). The comparison between the proportions of myeloid cells was not so straightforward due to differences at the annotations level between both reference datasets (Fig 13). The main difference lies on the $SPP1^+$ macrophages which were split into $SPP1^+$A and $SPP1^+$B macrophages in the annotations of the scRNA-seq dataset derived from the Belgian cohort. Interestingly, $SPP1^+$A and $SPP1^+$B macrophages were mapped to separate anatomical locations in the tumor proximity (Fig 14). Finally, we contrasted the results for the main stromal cell types (Fig 15). The overall results are highly consistent between both reference datasets with some minor but relevant differences. For instance, in the S1_Cec_Rep1 sample, a very limited content of Stromal 1 cells was detected when using the Korean dataset as compared to

the results using the Belgian dataset (Fig 16). Of note, the latter results matched with the regions annotated as stromal by the pathologist.

In summary, there was a large degree of similarity between the deconvolution results using either of the two references. In the context of our study, the most remarkable discrepancies arose from the spatial mapping of CMS1 tumor cells across our set of samples. We hypothesized that the annotations from the Korean dataset allowed the deconvolution to better distinguish between the signatures of CMS1 and CMS2 tumor cells. In samples S2_Col_R, S4_Col_Sig, S5_Rec and S6_Rec, the morphological features and the immune cold landscape of their tumors were suggestive of CMS2 carcinomas. These facts led to our choice of using in the main manuscript the deconvolution results obtained when using as reference the cell type annotations from the Korean cohort. Moreover, the number of individuals (23 versus 6) and annotated cells (65,362 versus 27,971) was larger in the Korean dataset than in the Belgian one, consequently reflecting a more comprehensive and diverse dataset for deconvolution purposes. On the other hand, some stromal populations derived from the Belgian dataset appear to better map their expected anatomical regions in a couple of our ST samples. We presume that the genetic background can have an influence on these results as our samples from German origin are expected to be more similar to the Belgian reference.

References

1. Kleshchevnikov, V. *et al.* Cell2location maps fine-grained cell types in spatial transcriptomics. *Nat. Biotechnol.* **40**, 661–671 (2022).
2. Lee, H.-O. *et al.* Lineage-dependent gene expression programs influence the immune landscape of colorectal cancer. *Nat. Genet.* **52**, 594–603 (2020).

Figures

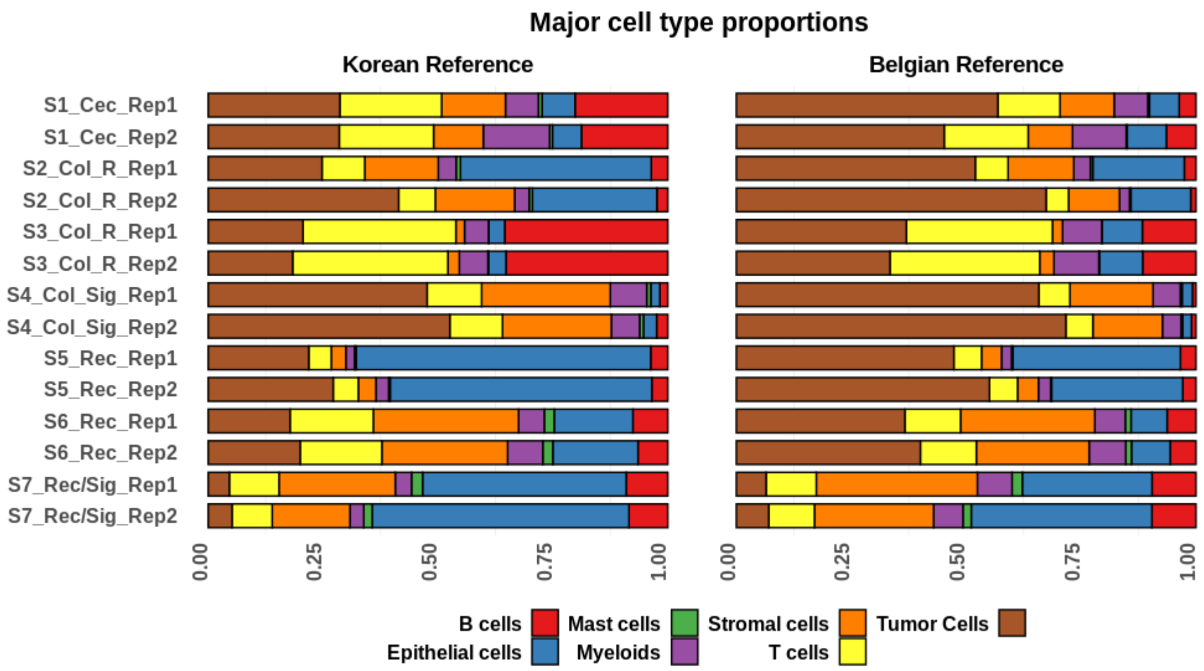

Figure 1: *Proportions of major cell classes per sample as estimated by the results of the deconvolution approach when using as reference the scRNA-seq dataset derived from either the Korean or the Belgian cohort.*

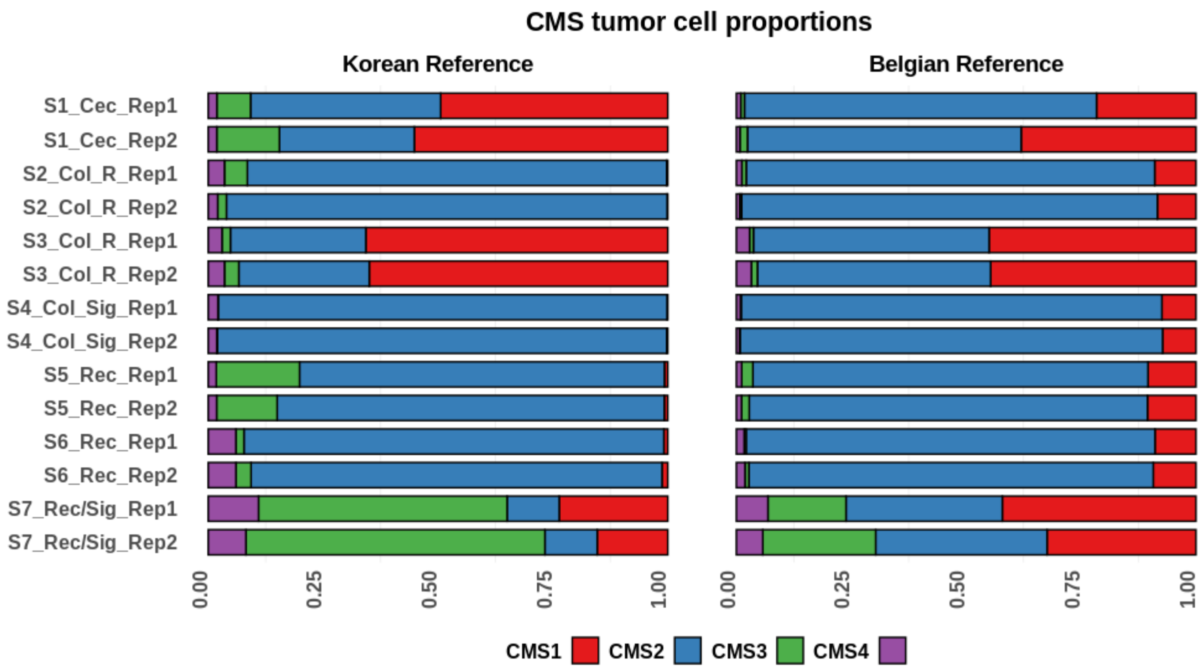

Figure 2: *Proportions of CMS tumor cells per sample as estimated by the results of the deconvolution approach when using as reference the scRNA-seq dataset derived from either the Korean or the Belgian cohort.*

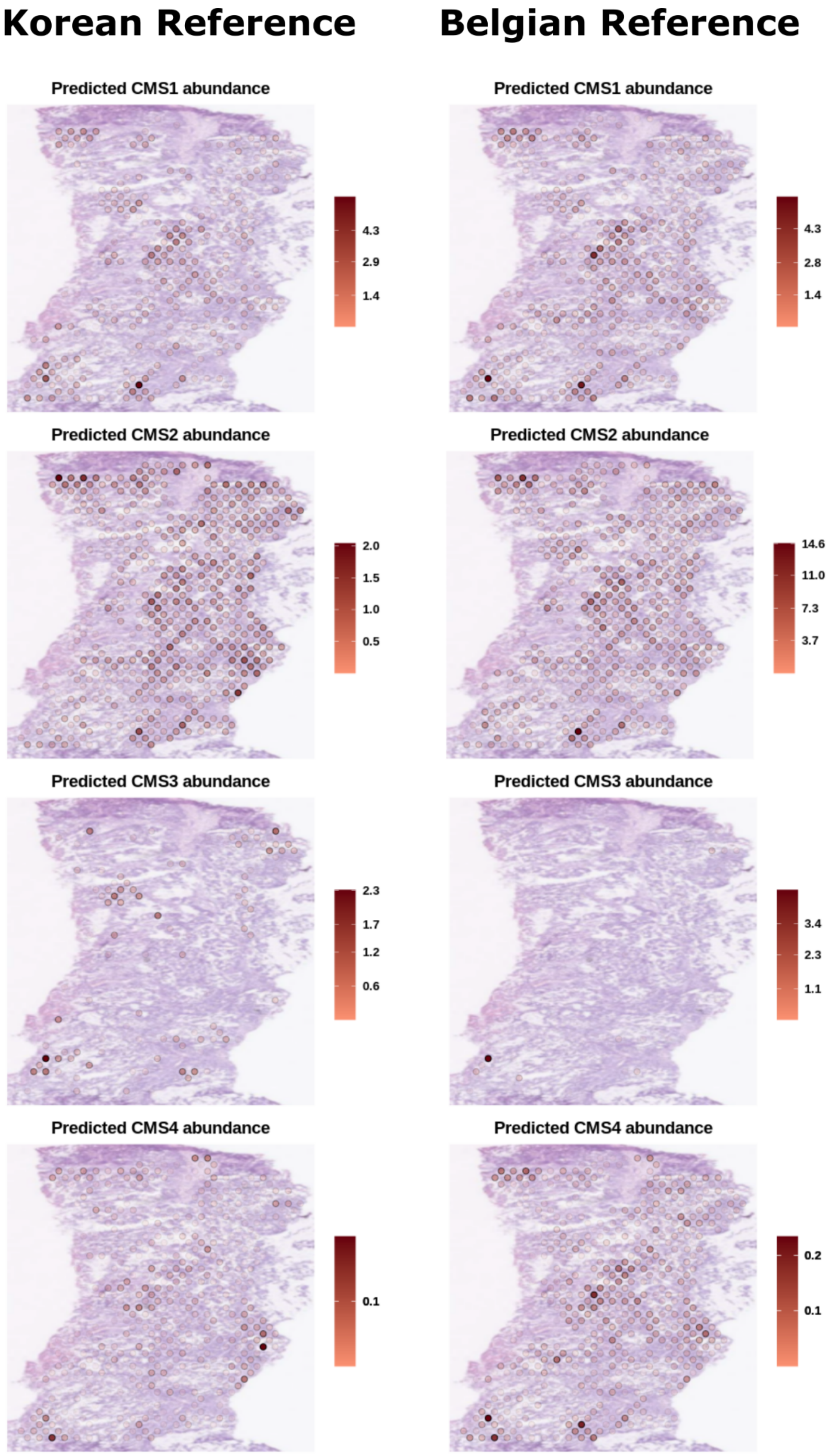

Figure 3: *Spatial mapping of the different CMS tumor cell abundances as predicted by the deconvolution when using as reference either the Korean (left panels) or the Belgian (right panels) datasets in the S1_Cec_Rep1 sample.*

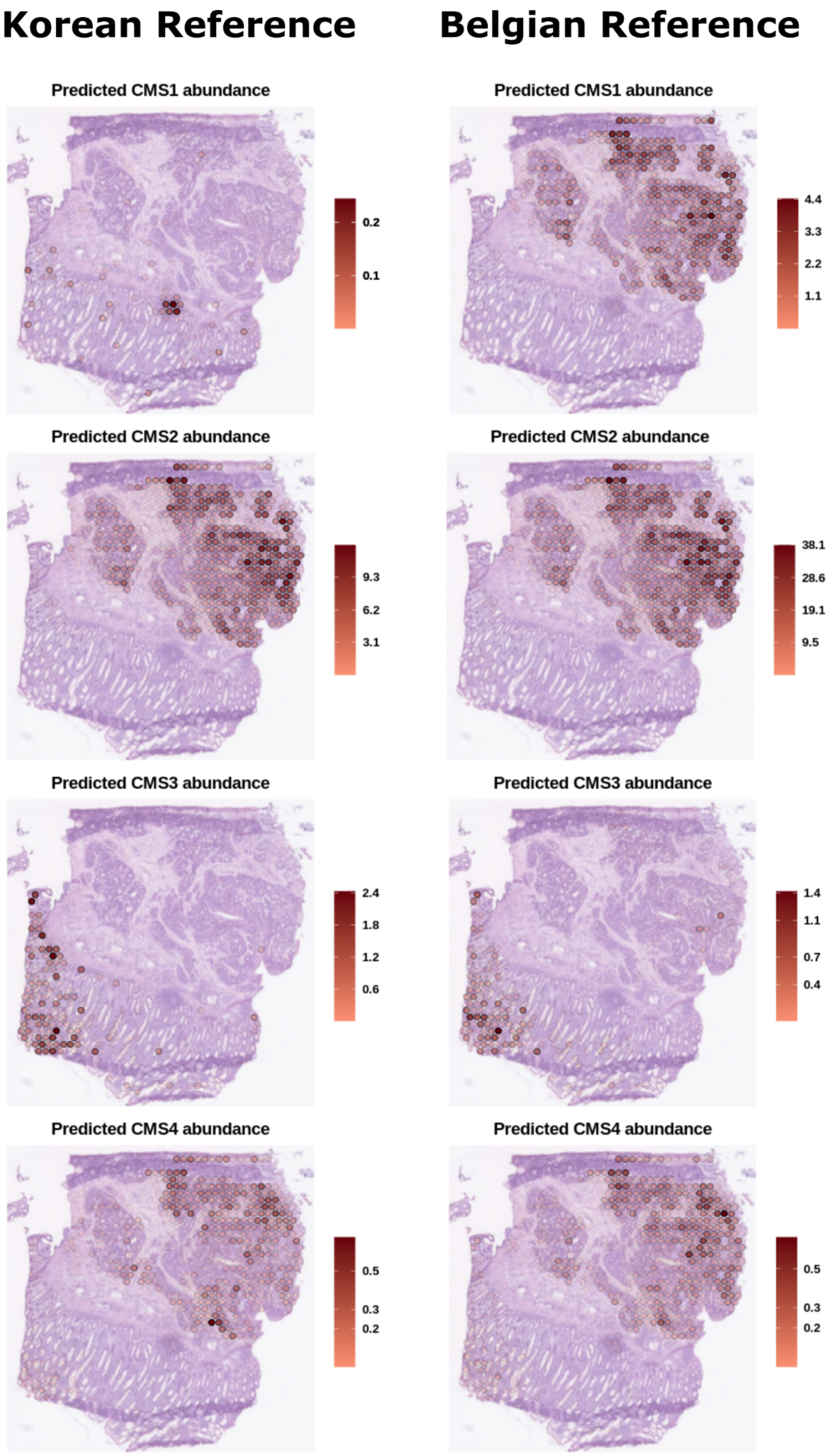

Figure 4: *Spatial mapping of the different CMS tumor cell abundances as predicted by the deconvolution when using as reference either the Korean (left panels) or the Belgian (right panels) datasets in the S2_Col_R_Rep1 sample.*

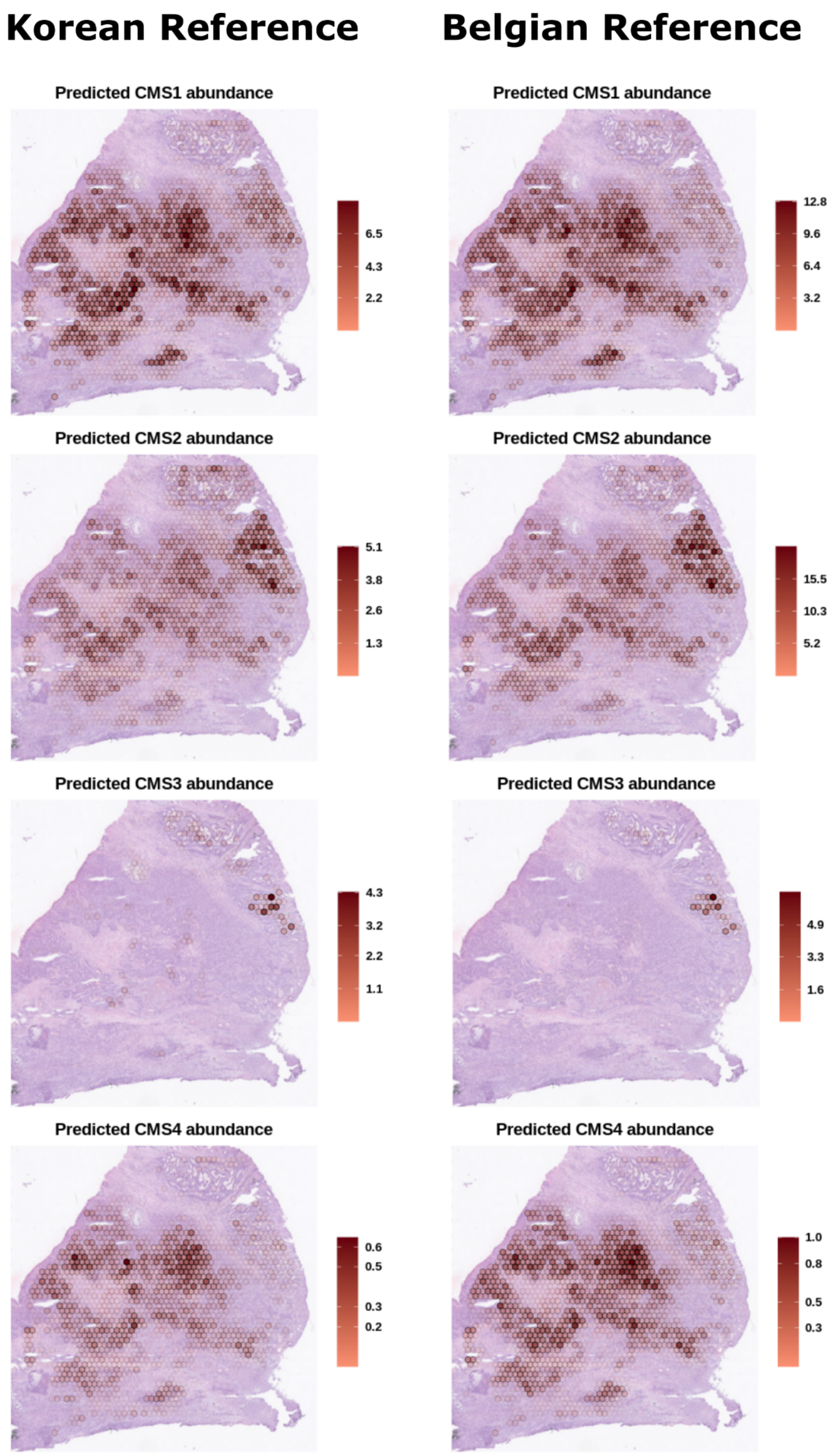

Figure 5: *Spatial mapping of the different CMS tumor cell abundances as predicted by the deconvolution when using as reference either the Korean (left panels) or the Belgian (right panels) datasets in the S3_Col_Rep1 sample.*

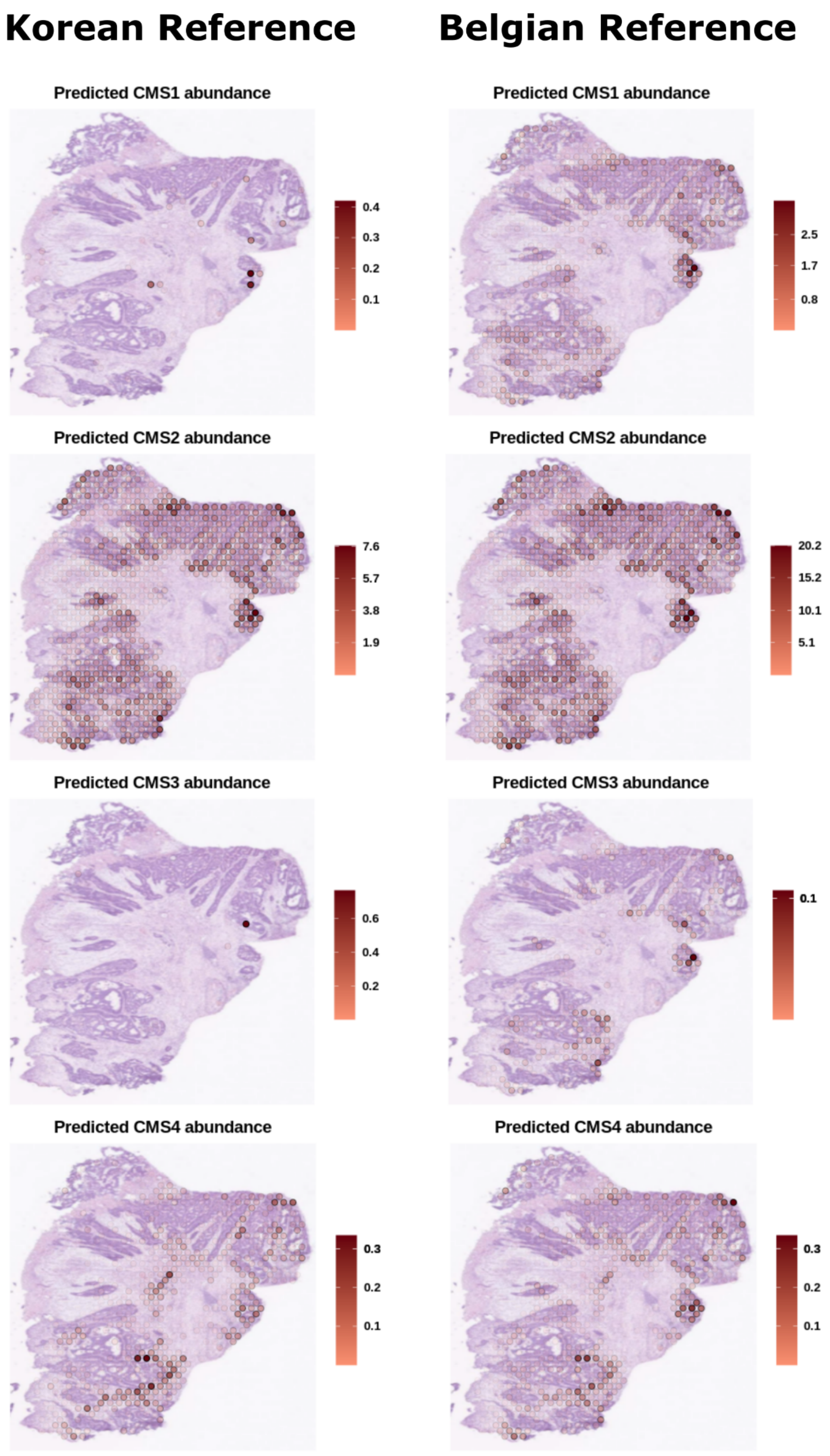

Figure 6: *Spatial mapping of the different CMS tumor cell abundances as predicted by the deconvolution when using as reference either the Korean (left panels) or the Belgian (right panels) datasets in the S4_Col_Sig_Rep2 sample.*

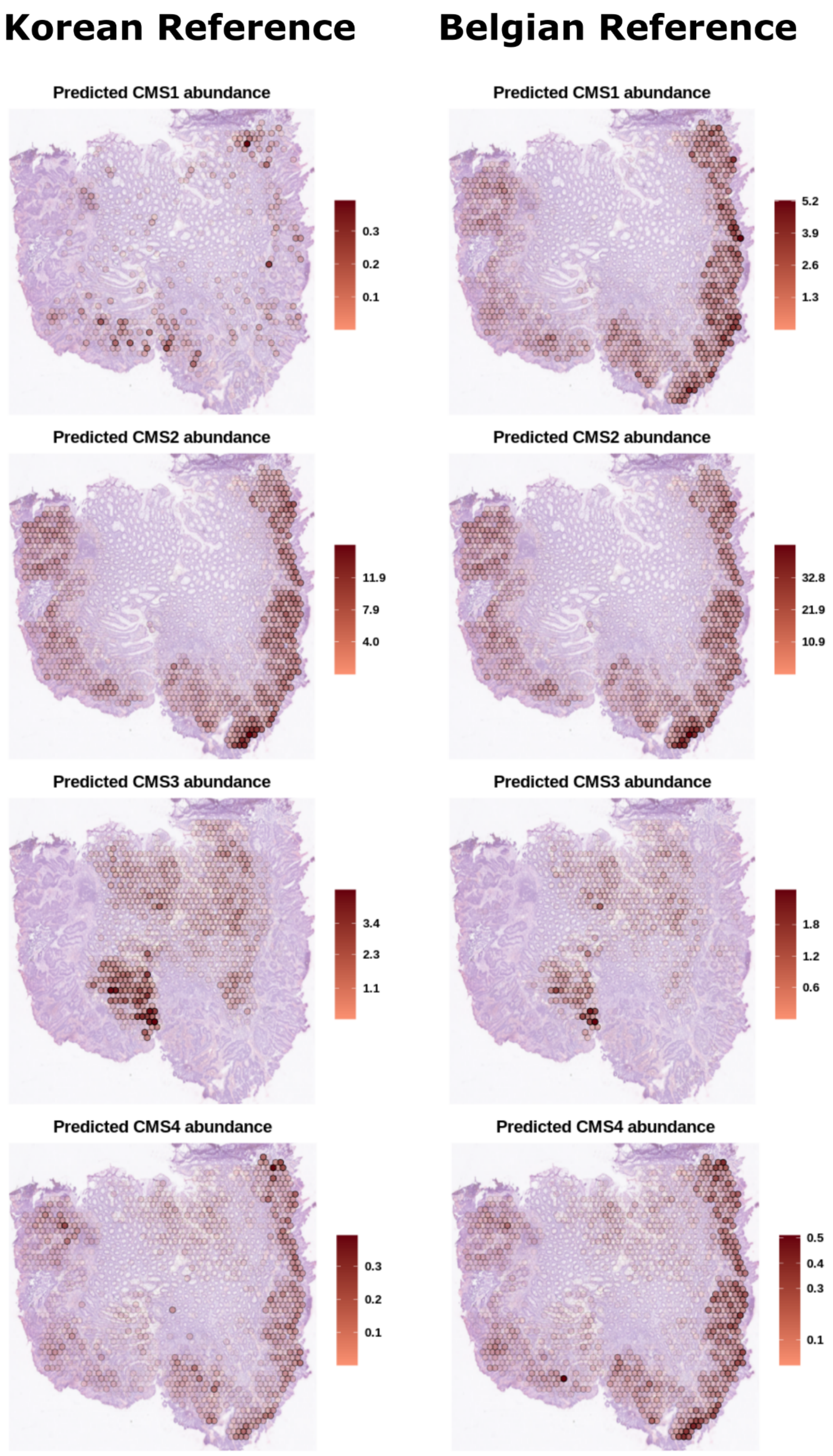

Figure 7: *Spatial mapping of the different CMS tumor cell abundances as predicted by the deconvolution when using as reference either the Korean (left panels) or the Belgian (right panels) datasets in the S5_Rec_Rep1 sample.*

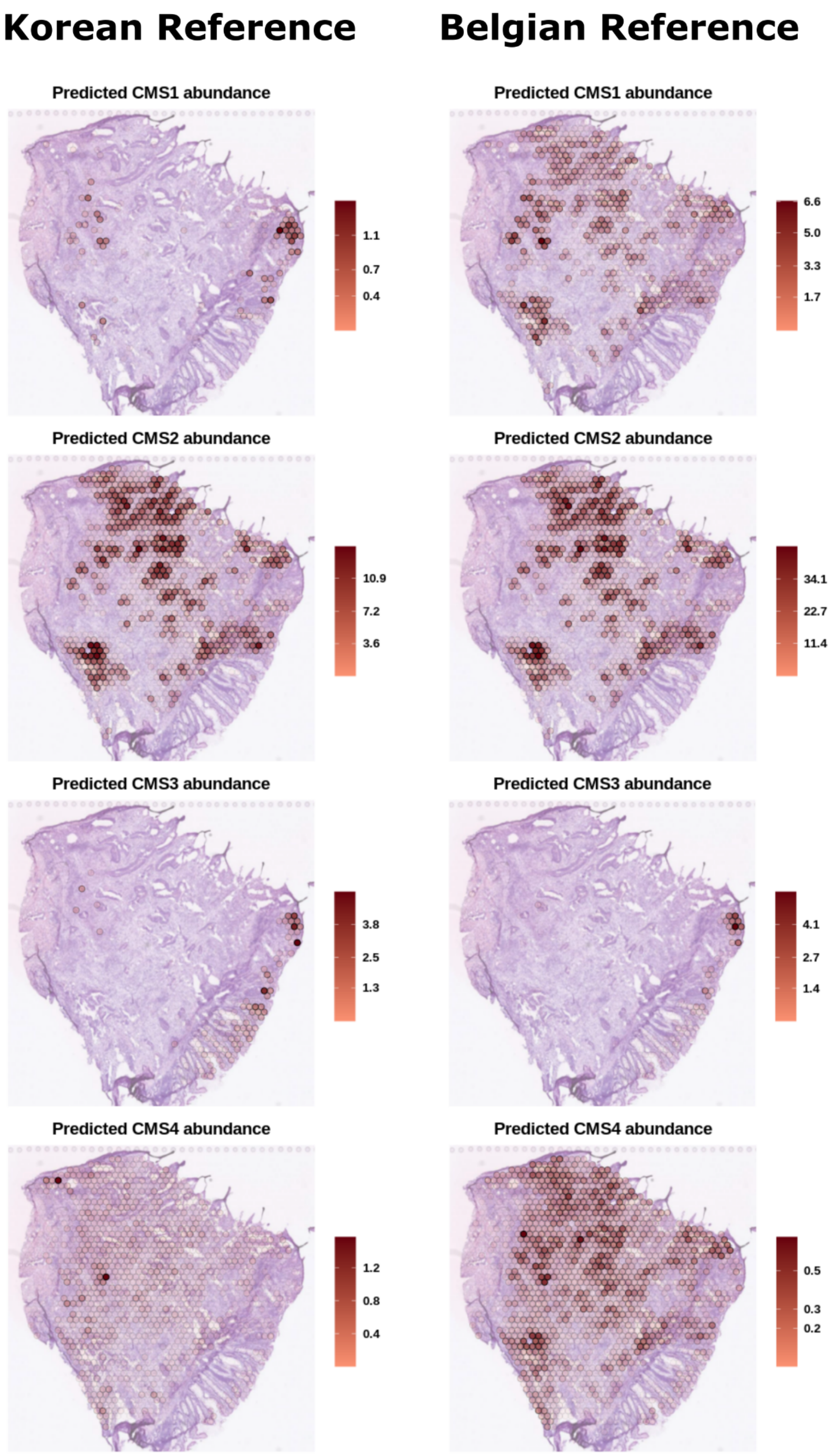

Figure 8: *Spatial mapping of the different CMS tumor cell abundances as predicted by the deconvolution when using as reference either the Korean (left panels) or the Belgian (right panels) datasets in the S6_Rec_Rep2 sample.*

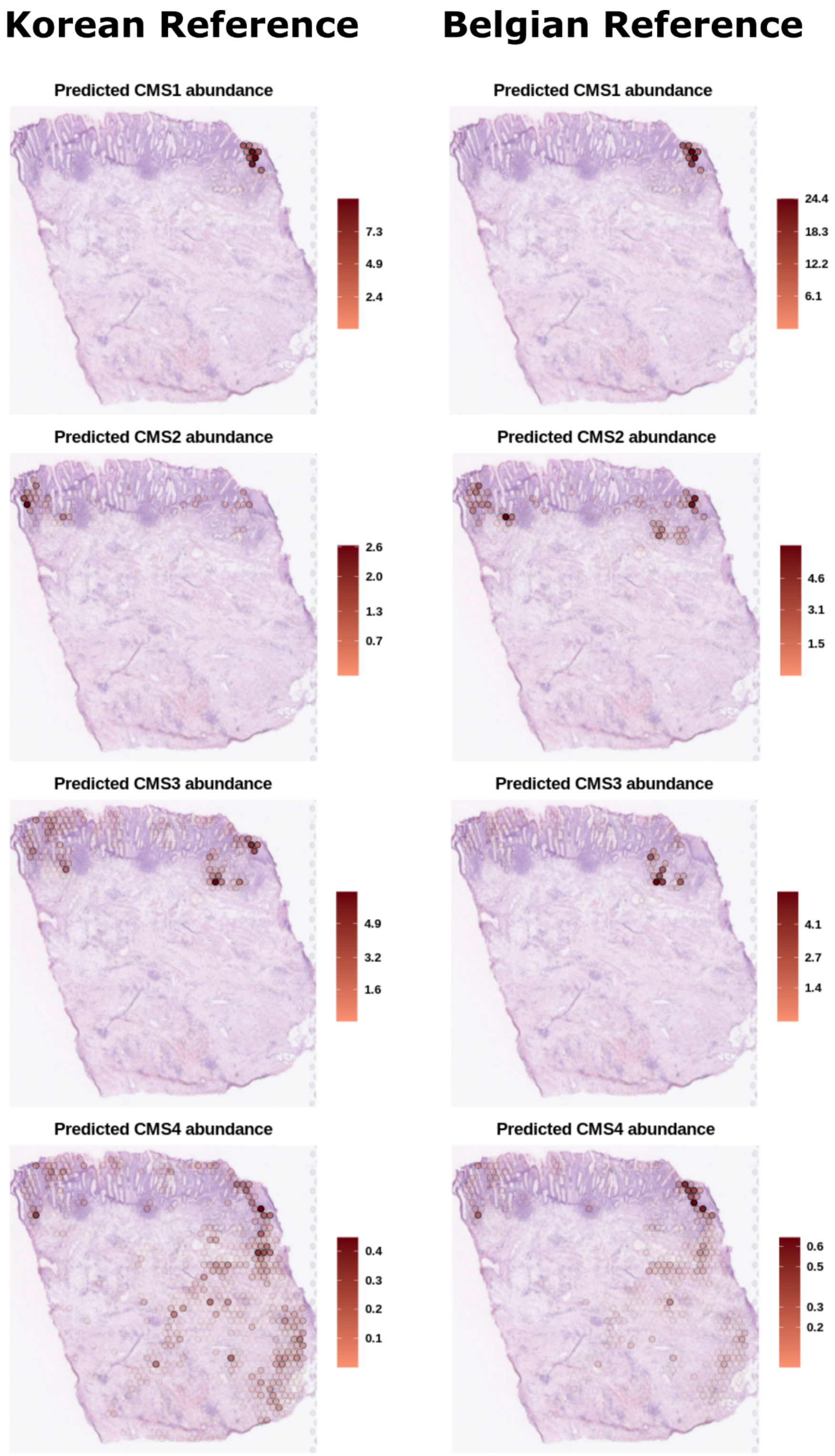

Figure 9: *Spatial mapping of the different CMS tumor cell abundances as predicted by the deconvolution when using as reference either the Korean (left panels) or the Belgian (right panels) datasets in the S7_Rec/Sig_Rep1 sample.*

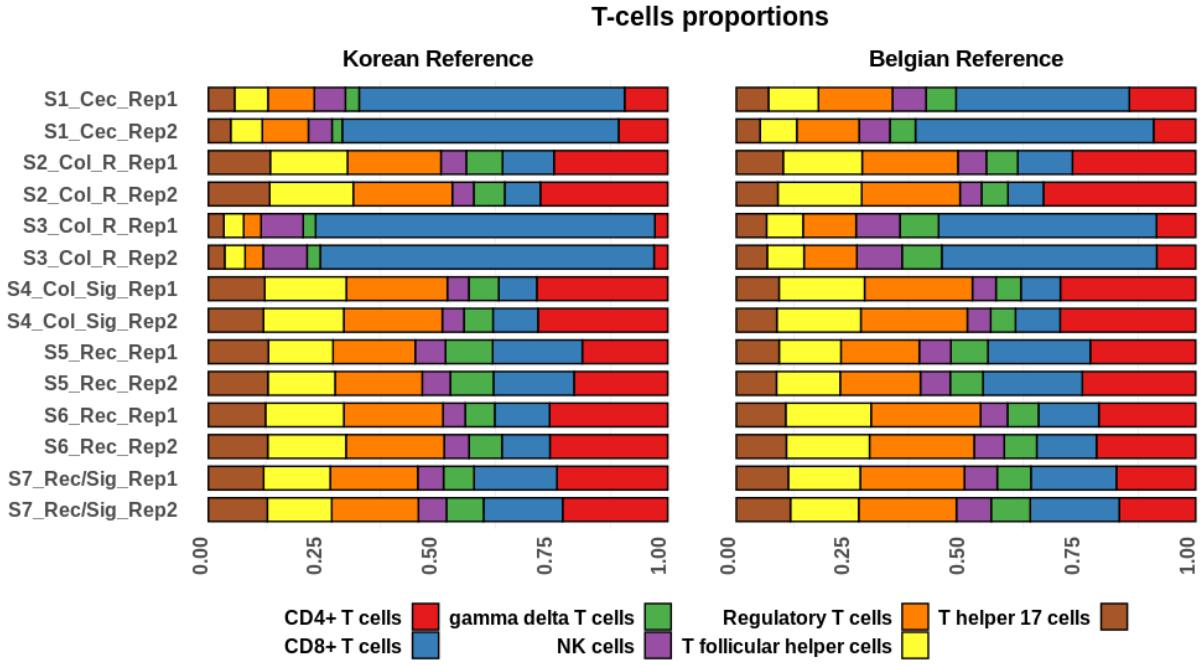

Figure 10: *Proportions of T-cells subtypes per sample as estimated by the results of the deconvolution approach when using as reference the scRNA-seq dataset derived from either the Korean or the Belgian cohort.*

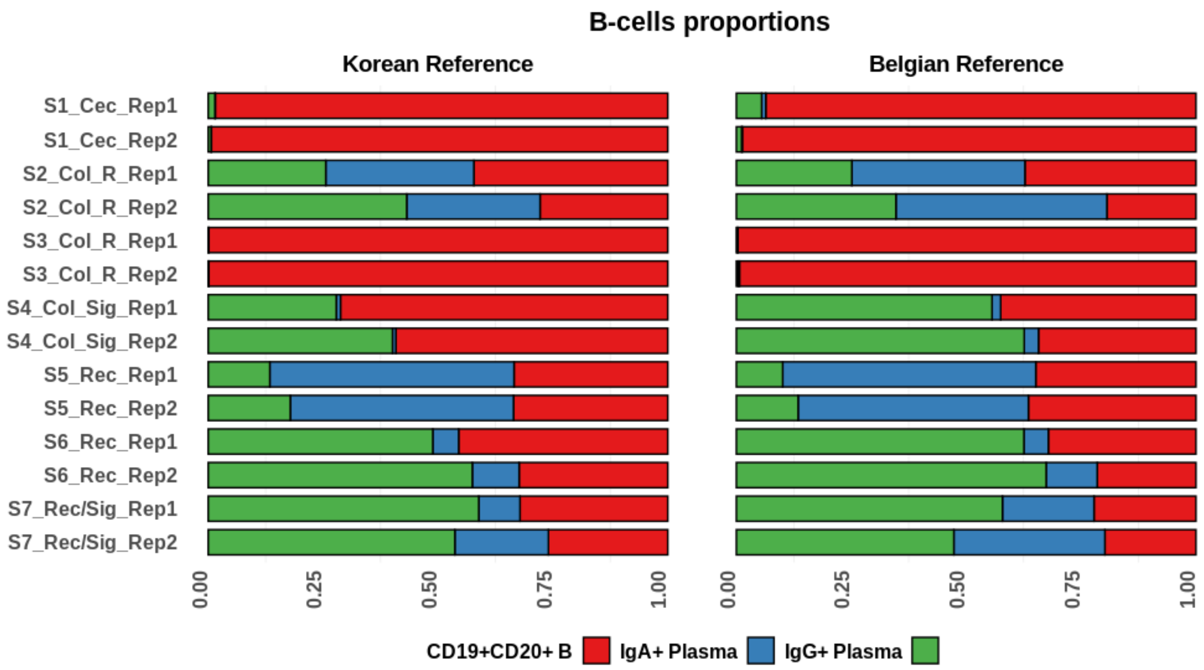

Figure 11: *Proportions of B-cells subtypes per sample as estimated by the results of the deconvolution approach when using as reference the scRNA-seq dataset derived from either the Korean or the Belgian cohort.*

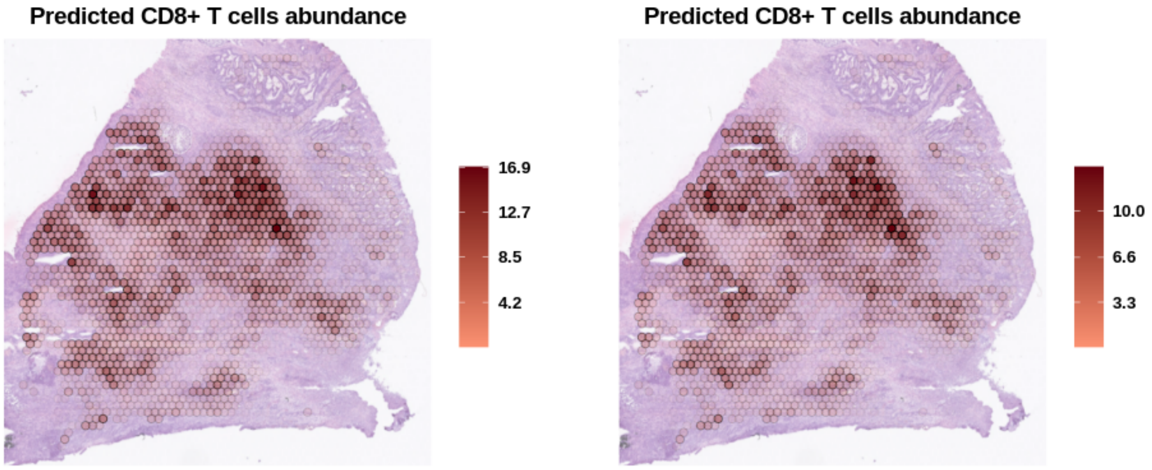

Figure 12: *Spatial mapping of the CD8+ T cells abundance as predicted by the deconvolution when using as reference either the Korean (left panel) or the Belgian (right panel) datasets in the S3_Col_Rep1 sample.*

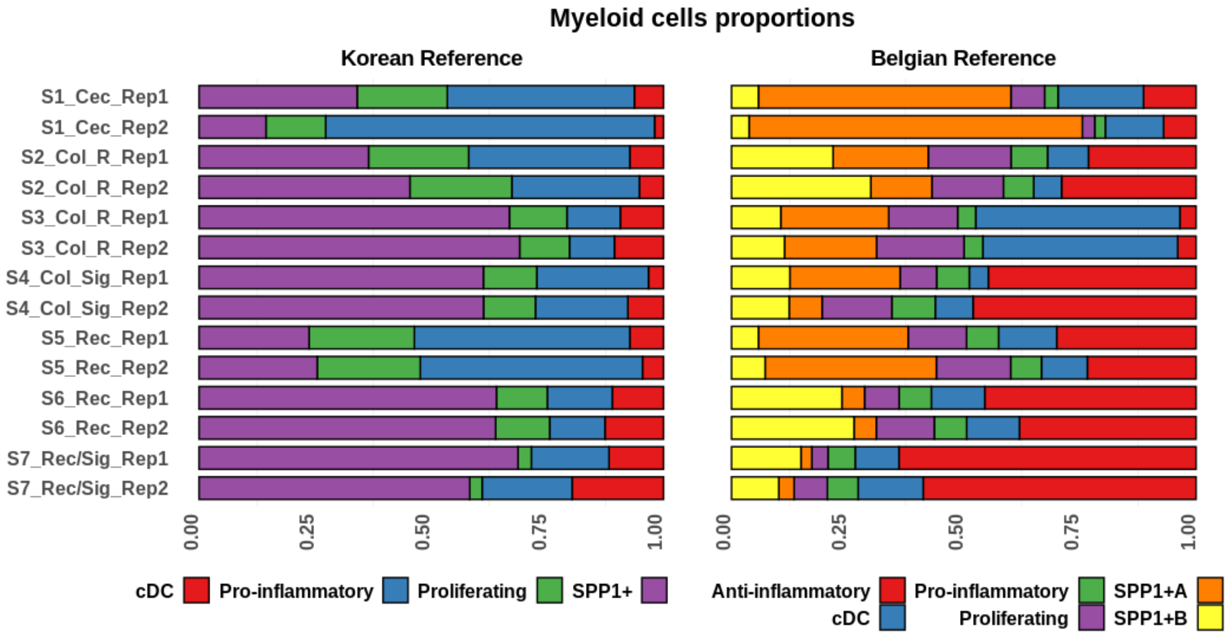

Figure 13: *Proportions of myeloid cell subtypes per sample as estimated by the results of the deconvolution approach when using as reference the scRNA-seq dataset derived from either the Korean or the Belgian cohort.*

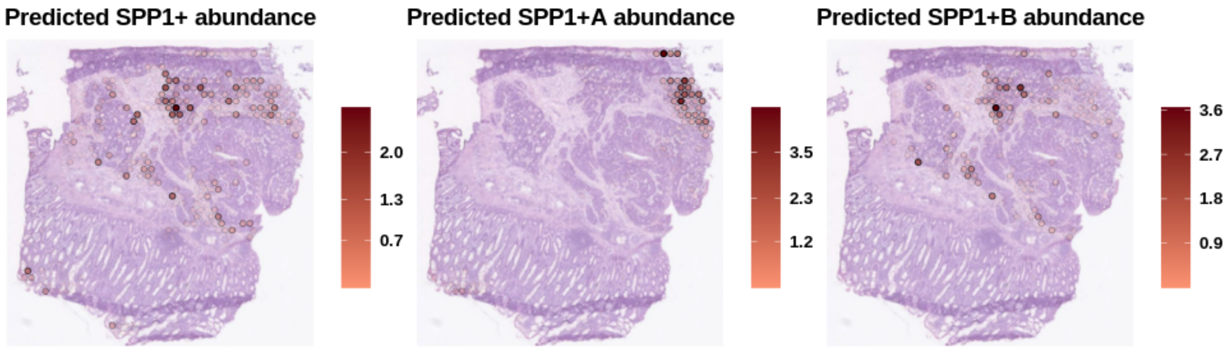

Figure 14: *Spatial mapping of the cell abundance of SPP1+ macrophages as predicted by the deconvolution when using as reference either the Korean (left panel) or the Belgian (central and right panels) datasets in the S2_Col_R_Rep1 sample.*

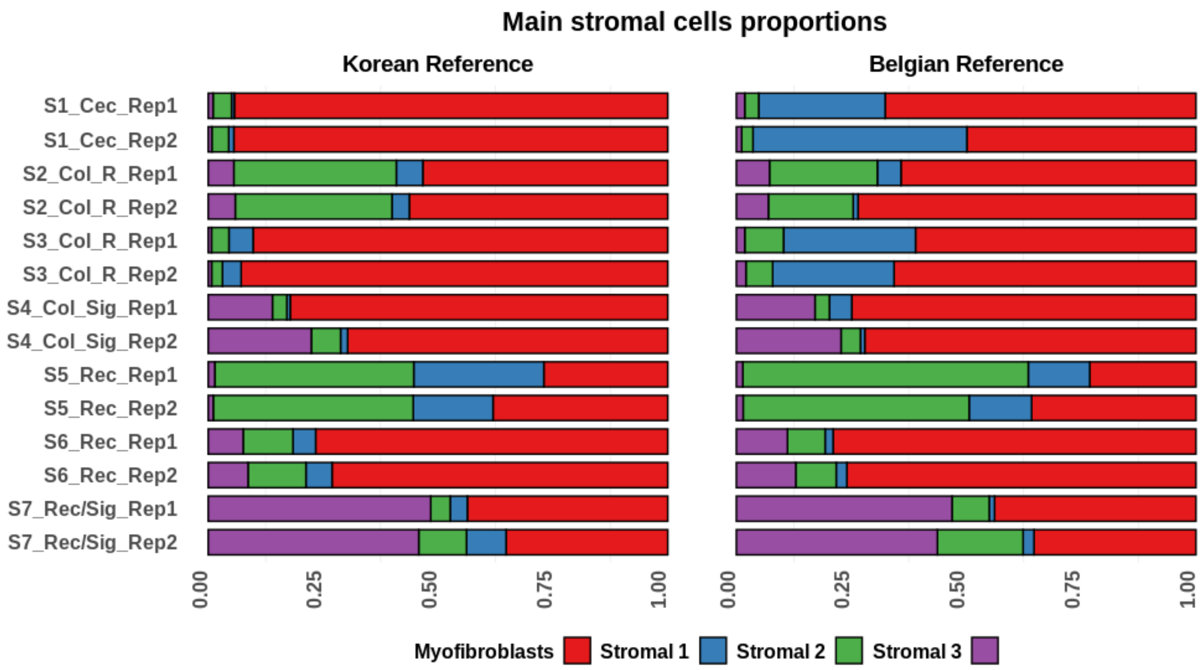

Figure 15: *Proportions of the main stromal cell subtypes per sample as estimated by the results of the deconvolution approach when using as reference the scRNA-seq dataset derived from either the Korean or the Belgian cohort.*

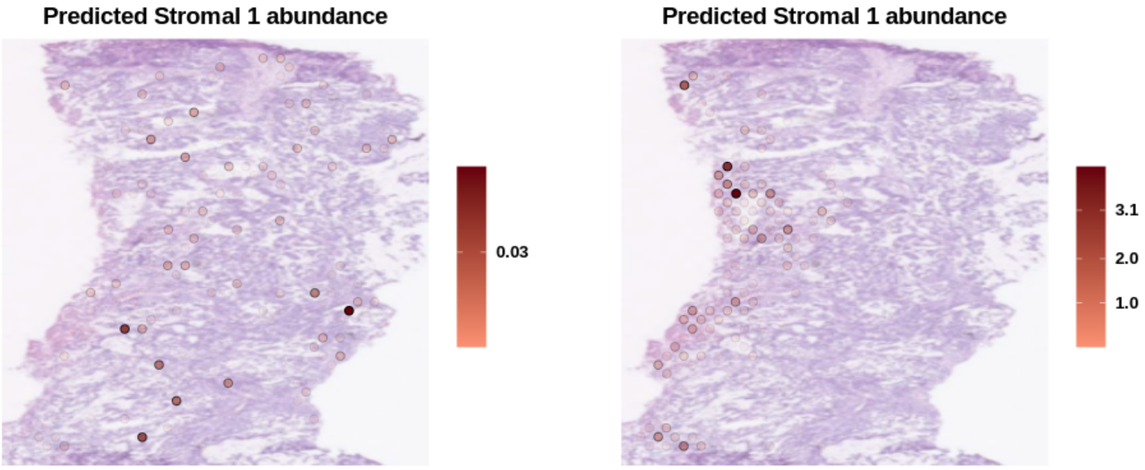

Figure 16: *Spatial mapping of the cell abundance of Stromal 1 cells as predicted by the deconvolution when using as reference either the Korean (left panel) or the Belgian (right panel) datasets in the S1_Cec_Rep1 sample.*
