# Supplementary material for: Profiling the heterogeneity of colorectal cancer consensus molecular subtypes using spatial transcriptomics
Source: NPJ Precis Oncol. 2024 Jan 10;8:10. doi: 10.1038/s41698-023-00488-4 (PMC10781769; doi:10.1038/s41698-023-00488-4)
Supplement: Supplementary file 2 — Reporting Summary [file 41698_2023_488_MOESM2_ESM.pdf]

nature portfolio

Corresponding author(s): Alberto Valdeolivas Urbelz

Last updated by author(s): Jul 14, 2023

Reporting Summary

Nature Portfolio wishes to improve the reproducibility of the work that we publish. This form provides structure for consistency and transparency in reporting. For further information on Nature Portfolio policies, see our Editorial Policies and the Editorial Policy Checklist.

Statistics

For all statistical analyses, confirm that the following items are present in the figure legend, table legend, main text, or Methods section.

n/a	Confirmed	
☐	☒	The exact sample size (*n*) for each experimental group/condition, given as a discrete number and unit of measurement
☐	☒	A statement on whether measurements were taken from distinct samples or whether the same sample was measured repeatedly
☐	☒	The statistical test(s) used AND whether they are one- or two-sided<br>*Only common tests should be described solely by name; describe more complex techniques in the Methods section.*
☒	☐	A description of all covariates tested
☐	☒	A description of any assumptions or corrections, such as tests of normality and adjustment for multiple comparisons
☐	☒	A full description of the statistical parameters including central tendency (e.g. means) or other basic estimates (e.g. regression coefficient) AND variation (e.g. standard deviation) or associated estimates of uncertainty (e.g. confidence intervals)
☐	☒	For null hypothesis testing, the test statistic (e.g. *F*, *t*, *r*) with confidence intervals, effect sizes, degrees of freedom and *P* value noted<br>*Give P values as exact values whenever suitable.*
☒	☐	For Bayesian analysis, information on the choice of priors and Markov chain Monte Carlo settings
☒	☐	For hierarchical and complex designs, identification of the appropriate level for tests and full reporting of outcomes
☐	☒	Estimates of effect sizes (e.g. Cohen's *d*, Pearson's *r*), indicating how they were calculated

Our web collection on statistics for biologists contains articles on many of the points above.

Software and code

Policy information about availability of computer code

Data collection	Custom code required to reproduce the analysis presented in the manuscript has been uploaded to: https://github.com/alberto-valdeolivas/ST_CRC_CMS. This includes all the required details about utilized software and specific versions.
Data analysis	Custom code required to reproduce the analysis presented in the manuscript has been uploaded to: https://github.com/alberto-valdeolivas/ST_CRC_CMS. This includes all the required details about utilized software and specific versions.

For manuscripts utilizing custom algorithms or software that are central to the research but not yet described in published literature, software must be made available to editors and reviewers. We strongly encourage code deposition in a community repository (e.g. GitHub). See the Nature Portfolio guidelines for submitting code & software for further information.

Data

Policy information about availability of data

All manuscripts must include a data availability statement. This statement should provide the following information, where applicable:

- Accession codes, unique identifiers, or web links for publicly available datasets
- A description of any restrictions on data availability
- For clinical datasets or third party data, please ensure that the statement adheres to our policy

The output of Space Ranger, including processed count data matrices and histological images, for the ST data generated in this study is available at https://doi.org/10.5281/zenodo.7551712. In addition, this repository also contains the spot categorization made by the pathologist. The processed scRNA-seq and

metadata used for the deconvolution and for further characterization of the cell communication processes are available via the GEO database under the accession codes GSE132465 and GSE1447356. The processed data from the external ST CRC dataset used to support our findings was downloaded from http://www.cancerdiversity.asia/scCRLM14.

Research involving human participants, their data, or biological material

Policy information about studies with human participants or human data. See also policy information about sex, gender (identity/presentation), and sexual orientation and race, ethnicity and racism.

Reporting on sex and gender	Our study includes data from 4 males and 3 human females.
Reporting on race, ethnicity, or other socially relevant groupings	6 out of the 7 participants were identified as Europeans. This information was not disclosed for the remaining participant.
Population characteristics	Age:<br>- 1 participant was in the range of 50-59.<br>- 1 participant was in the range of 60-69.<br>- 4 participants were in the range of 70-79.<br>- 1 participant was in the range of 80-89.
Recruitment	Human CRC tissues (<8 months storage) and annotated data were obtained and experimental procedures were performed within the framework of the non profit foundation HTCR, including the informed patient's consent50.
Ethics oversight	the non profit foundation HTCR

Note that full information on the approval of the study protocol must also be provided in the manuscript.

Field-specific reporting

Please select the one below that is the best fit for your research. If you are not sure, read the appropriate sections before making your selection.

[X] Life sciences [ ] Behavioural & social sciences [ ] Ecological, evolutionary & environmental sciences

For a reference copy of the document with all sections, see nature.com/documents/nr-reporting-summary-flat.pdf

Life sciences study design

All studies must disclose on these points even when the disclosure is negative.

Sample size	We have a cohort of 7 patients (plus 1 detailed below whose samples were discarded due to low quality). Technical replicates were considered as detailed below. Therefore, the sample size of the analyzed data presented in this study is n=14. Our study is quite exploratory so we did not need a large sample size, but rather enough samples to generate reliable hypothesis that we be later on validated.
Data exclusions	We excluded one of the collected samples due to low quality RNA integrity.
Replication	For every patient, consecutive tissue sections were taken to consider technical replicates.
Randomization	We randomized the position of samples and replicates across the 10x VISIUM sections.
Blinding	None

Reporting for specific materials, systems and methods

We require information from authors about some types of materials, experimental systems and methods used in many studies. Here, indicate whether each material, system or method listed is relevant to your study. If you are not sure if a list item applies to your research, read the appropriate section before selecting a response.

Materials & experimental systems

n/a	Involved in the study
☒	☐ Antibodies
☒	☐ Eukaryotic cell lines
☒	☐ Palaeontology and archaeology
☒	☐ Animals and other organisms
☒	☐ Clinical data
☒	☐ Dual use research of concern
☒	☐ Plants

Methods

n/a	Involved in the study
☒	☐ ChIP-seq
☒	☐ Flow cytometry
☒	☐ MRI-based neuroimaging
